# Supplementary material for: Disrupting metformin adaptation of liver cancer cells by targeting the TOMM34/ATP5B axis
Source: EMBO Mol Med. 2022 Nov 2;14(12):e16082. doi: 10.15252/emmm.202216082 (PMC9728056; doi:10.15252/emmm.202216082)
Supplement: Supplementary file 1 — Appendix [file EMMM-14-e16082-s006.pdf]

Appendix

Disrupting metformin adaptation of liver cancer cells by targeting the TOMM34/ATP5B axis

Ping Jin[1†], Jingwen Jiang[1†], Li Zhou[1], Zhao Huang[1], Siyuan Qin[1], Hai-Ning Chen[2], Liyuan Peng[1], Zhe Zhang[1], Bowen Li[1], Maochao Luo[1], Tingting Zhang[1], Hui Ming[3], Ning Ding[4], Lei Li[4], Na Xie[3], Wei Gao[5], Wei Zhang[6], Edouard C. Nice[7], Yuquan Wei[8], Canhua Huang[1*]

Table of Contents

Appendix Figures S1 – S5 with their legends

Appendix Tables S1

Appendix Fig. S1. TOMM34 promotes cancer cell growth under various metabolic stress conditions.

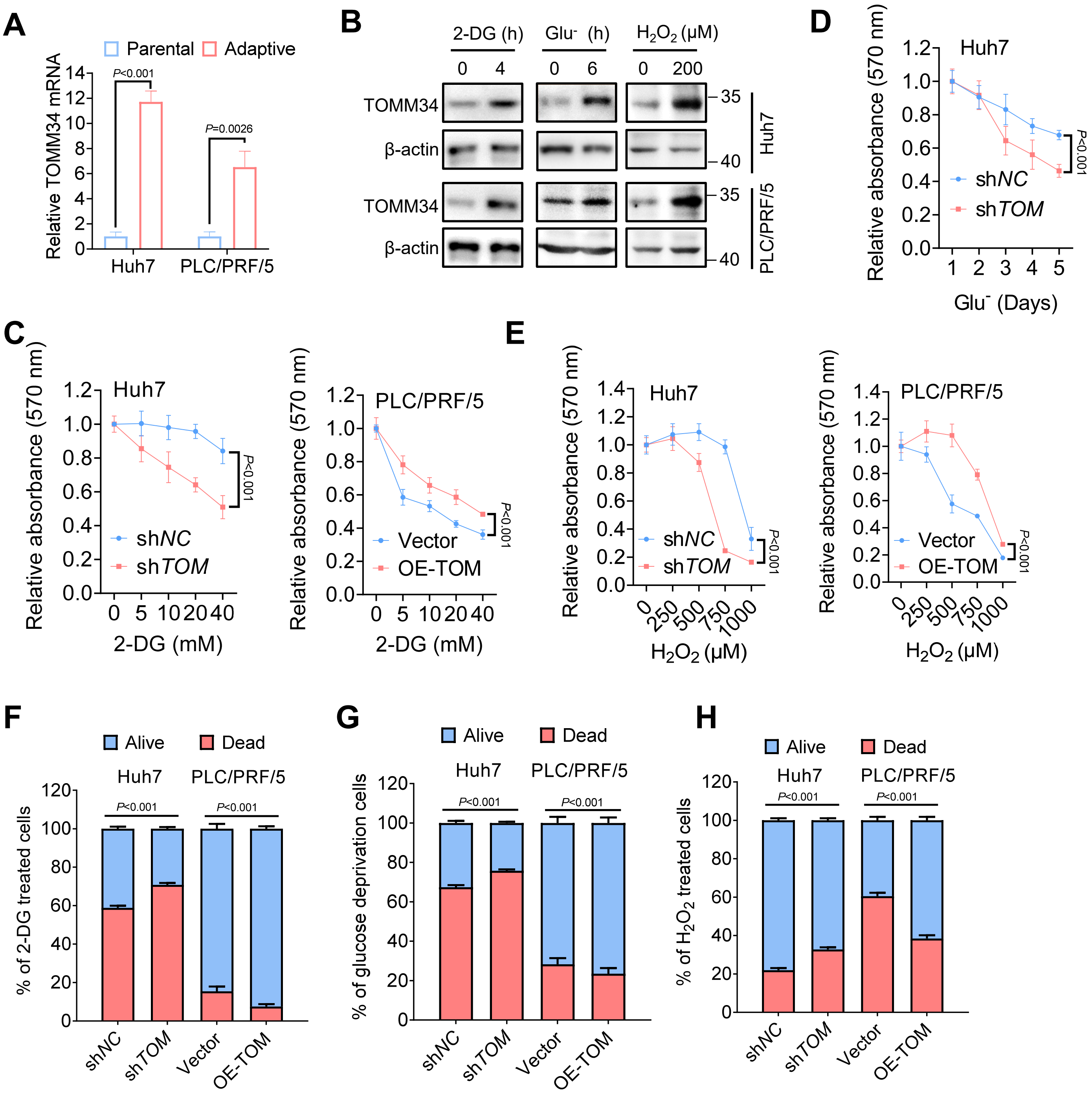

Appendix Fig. S1. TOMM34 promotes cancer cell growth under various metabolic stress conditions.

(**A**) qPCR analysis showing the mRNA levels of TOMM34 in parental or metformin adaptive HCC cells. (n=3 technical replicates, Two-way ANOVA).

(**B**) Western blot analysis showing effects of metabolic stress (5 mM 2-DG, glucose deprivation or 200 μM H_2O_2 treatment) on TOMM34 expression.

(**C-E**) MTT assay indicating the effects of TOMM34 on HCC cells viability under metabolic stress for indicated days (D) or 48 hours (C, E) (sh*TOM*: sh*TOMM34*, TOMM34 knockdown cells; OE-TOM: OE-TOMM34, TOMM34-overexpressed cells). (n=4 biological replicates, Two-way ANOVA).

(**F-H**) Trypan blue assay showing the effects of TOMM34 on maintaining cancer cell survival upon metabolic stress (sh*TOM*: sh*TOMM34*, TOMM34 knockdown cells; OE-TOM: OE-TOMM34, TOMM34-overexpressed cells). (n=4 biological replicates, two-way ANOVA).

Data information: Data are presented as means ± S.D.

Source data are available online for this figure.

Appendix Fig. S2. TOMM34 promotes metastasis during metformin adaptation.

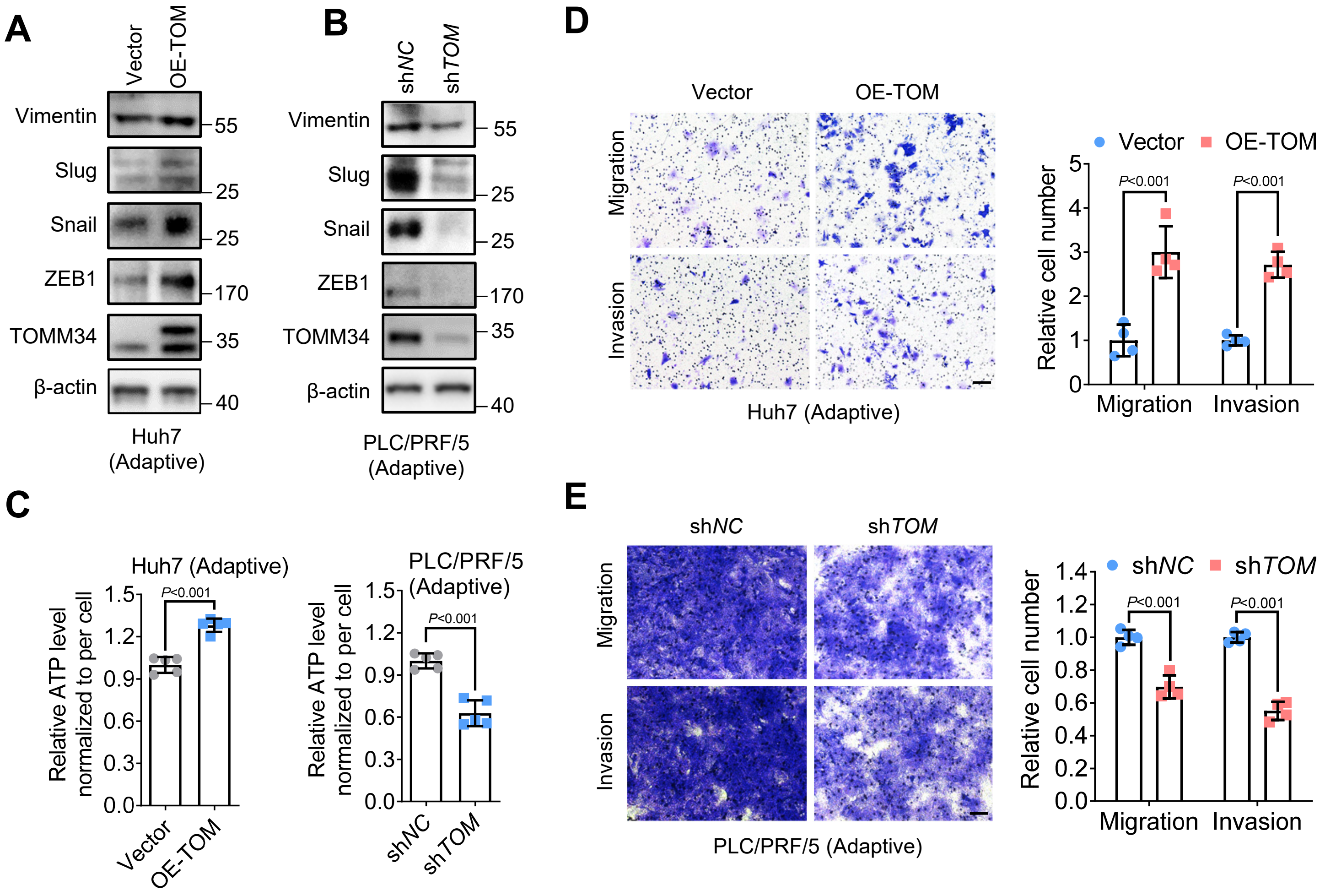

Appendix Fig. S2. TOMM34 promotes metastasis during metformin adaptation.

(**A-B**) Western blot showing the expression of EMT markers in adaptive HCC cells with or without the knockdown or overexpression of TOMM34 (sh*TOM*: sh*TOMM34*, TOMM34 knockdown cells; OE-TOM: OE-TOMM34, TOMM34-overexpressed cells).

(**C**) Cellular ATP level of adaptive HCC cells with or without knockdown or overexpression of TOMM34 (sh*TOM*: sh*TOMM34*, TOMM34 knockdown cells; OE-TOM: OE-TOMM34, TOMM34-overexpressed cells). (n=5 biological replicates, Student's t-test).

(**D-E**) Transwell assays showing the migration and invasion of adaptive HCC cells with or without knockdown or overexpression of TOMM34 (2×10^4 adaptive Huh7 cells, 1×10^5 adaptive PLC/PRF/5 cells; sh*TOM*: sh*TOMM34*, TOMM34 knockdown cells; OE-TOM: OE-TOMM34, TOMM34-overexpressed cells). Scale bars, 100 µm. (n=4 biological replicates, Two-way ANOVA).

Appendix Fig. S3. TOMM34 facilitates mitochondrial function in HCC cells.

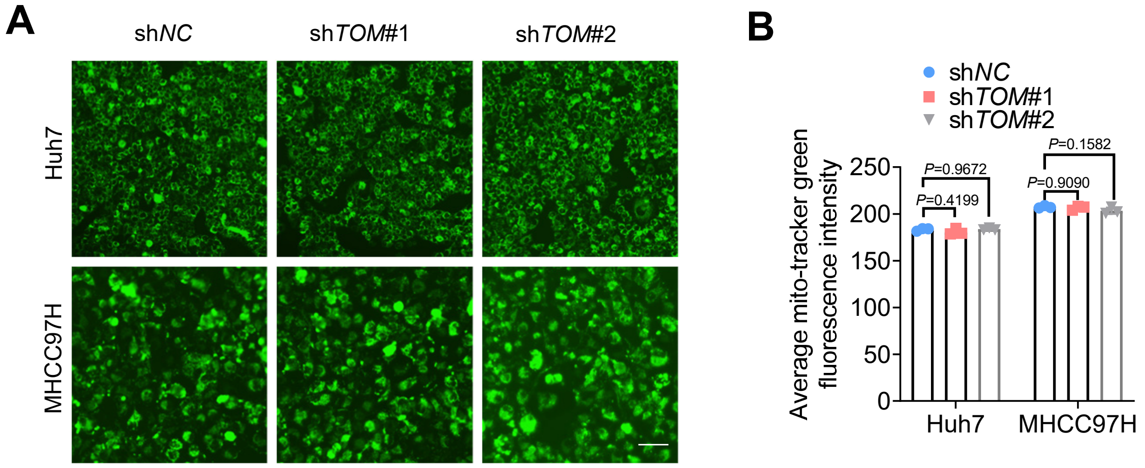

Appendix Fig. S3. TOMM34 facilitates mitochondrial function in HCC cells.

(**A-B**) Mito-Tracker green assay showing mitochondrial numbers in indicated cells (sh*TOM*: sh*TOMM34*, TOMM34 knockdown cells). (n=3 biological replicates, Two-way ANOVA). Scale bars, 100 μm.

Appendix Fig. S4. ATP5B overexpression is correlated with HCC progression and poor prognosis.

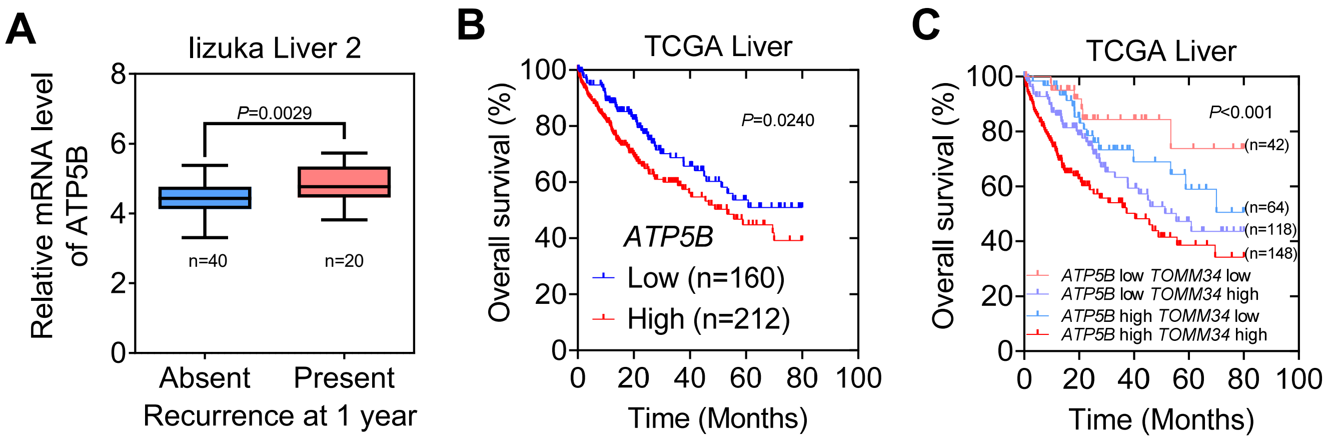

Appendix Fig. S4. ATP5B overexpression is correlated with HCC progression and poor prognosis.

(**A**) ATP5B mRNA levels in HCC patients with or without recurrence at one year according to TCGA data set Iizuka Liver. (https://www.thelancet.com/journals/lancet/article/PIIS0140-6736(03)12775-4/fulltext).

(**B**) HCC patients were stratified into two groups according to their TOMM34 mRNA level, followed by the survival analysis. Statistical difference was determined using log-rank (Mantel-Cox) test.

(**C**) HCC patients from TCGA dataset (https://portal.gdc.cancer.gov/projects/TCGA-LIHC) were stratified into four groups according to their TOMM34 and ATP5B mRNA level, followed by the survival analysis. Statistical difference was determined using log-rank (Mantel-Cox) test.

Appendix Fig. S5. Gboxin impairs TOMM34-mediated migration of HCC cells.

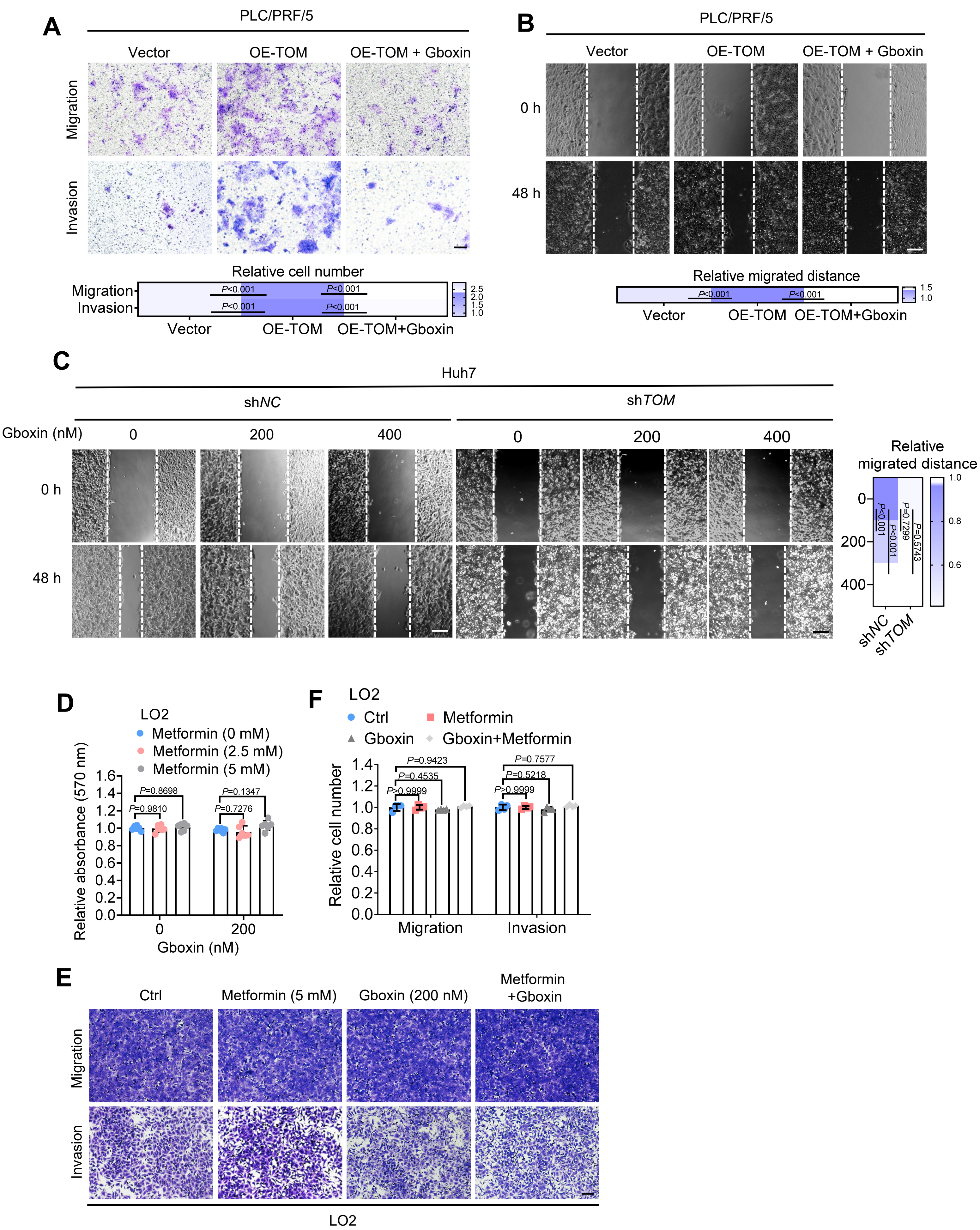

Appendix Fig. S5. Gboxin impairs TOMM34-mediated migration of HCC cells.

(**A**) Transwell assay showing the migration and invasion of PLC/PRF/5 cells treated with or without 200 nM Gboxin for 36 hours (1×10^5 cells; OE-TOM: OE-TOMM34, TOMM34-overexpressed cells). Scale bars, 100 µm. (n=3 biological replicates, Two-way ANOVA).

(**B**) Wound healing assay showing migration of PLC/PRF/5 cells overexpressing TOMM34 treated with or without 200 nM Gboxin for 48 hours (OE-TOM: OE-TOMM34, TOMM34-overexpressed cells). Scale bars, 200 µm. (n=3 technical replicates, Two-way ANOVA).

(**C**) Wound healing assay showing the migration of Huh7 sh*NC* and sh*TOM* (sh*TOMM34*) cells treated with or without Gboxin as indicated. Scale bars, 200 µm. (n=3 technical replicates, Two-way ANOVA).

(**D**) MTT assays showing the proliferation of liver non-cancerous cells LO2 treated with or without metformin or Gboxin at indicated concentration for 24 hours. (n=6 biological replicates, Two-way ANOVA).

(**E**-**F**) Transwell assays showing the migration and invasion of LO2 cells treated with or without metformin or Gboxin at indicated concentration for 24 hours (1×10^5 cells). Scale bars, 100 µm. (n=4 biological replicates, Two-way ANOVA).

Appendix table S1. Clinicopathologic characteristics of liver cancer patients.

Characteristic	Cohort (n)
Total cases	72
Age,yr (median,range)	52 (18-76)
Gender	
Female	15 (20.8%)
Male	57 (79.2%)
Grades	
I、 I-II	7 (9.7%)
II、II-III	38 (52.8%)
III	27 (37.5%)
Histologic type	
Hepatocellular carcinoma	65 (90.3%)
Cholangiocarcinoma	7 (9.7%)
