# Supplementary material for: Disrupting metformin adaptation of liver cancer cells by targeting the TOMM34/ATP5B axis
Source: EMBO Mol Med. 2022 Nov 2;14(12):e16082. doi: 10.15252/emmm.202216082 (PMC9728056; doi:10.15252/emmm.202216082)
Supplement: Supplementary file 2 — Expanded View Figures PDF [file EMMM-14-e16082-s007.pdf]

Expanded View Figures

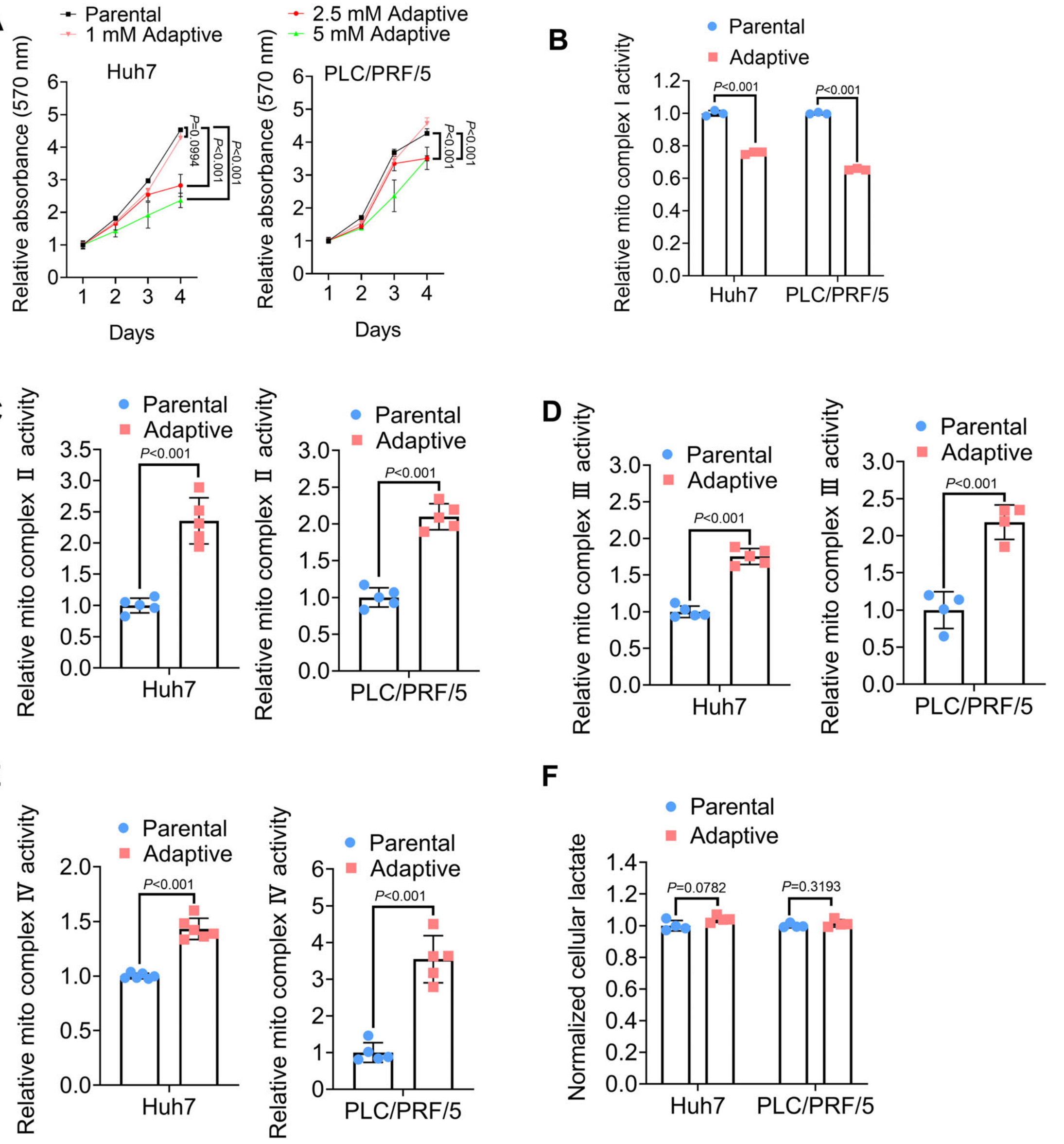

Figure EV1. Metformin-adaptive HCC cells exhibit hyperactivity of mitochondrial respiration.

A MTT assays showing the growth of parental HCC cells and metformin-adaptive HCC cells (obtained from long-term exposure to different concentrations of metformin). (n = 6 biological replicates, Two-way ANOVA).

B–E The activity of mitochondrial complex I, II, III and IV in parental and adaptive HCC cells was measured. (n = 3 technical replicates in B, n = 5 technical replicates in C, n = 5 or 4 technical replicates in D, n = 6 or 5 technical replicates in E, Student's t-test).

F Cellular lactate level of parental and adaptive HCC cells. (n = 4 biological replicates, Two-way ANOVA).

Data information: Data are presented as means ± SD.

 © 2022 The Authors

Figure EV2. TOMM34 upregulates the metastatic potential of HCC.

A The protein level of TOMM34 in PDX models with or without long-term metformin treatment was evaluated by immunohistochemistry. Scale bars, (Left) 1,000 μm; (Right) 25 μm. (n = 5 biological replicates in each group, Student's t-test).

B Schematic diagram for the HCC lung metastasis model in the orthotopic injection mouse. HE and IHC assays showing the histology and TOMM34 expression. Scale bars, 100 or 25 μm.

C, D Transwell assays showing migration and invasion ability of Huh7 and PLC/PRF/5 cells (5×10^4 Huh7 cells, 1×10^5 PLC/PRF/5 cells; sh*TOM*, sh*TOMM34*, TOMM34 knockdown cells; OE-TOM, OE-TOMM34, TOMM34-overexpressed cells). Scale bars, 100 μm. (n = 3 biological replicates, Two-way ANOVA).

E, F Wound healing assay showing migration of HCC cells. Scale bars, 200 μm. (n = 6 technical replicates, Student's t-test).

G Alterations in the morphology of HCC cells with or without the overexpression or knockdown of TOMM34 (sh*TOM*, sh*TOMM34*, TOMM34 knockdown cells; OE-TOM, OE-TOMM34, TOMM34-overexpressed cells). Scale bars, 100 μm.

H TOMM34 mRNA levels in HCC patients with or without metastasis according to TCGA data set GSE3500 (https://www.ncbi.nlm.nih.gov/geo/query/acc.cgi?acc=GSE3500). (One-way ANOVA). The boxplot represents the minimum value, the second quartile, the median value, the third quartile, and the maximum value.

I Overall survival of HCC patients from TCGA dataset (https://portal.gdc.cancer.gov/projects/TCGA-LIHC). Patients were stratified into High and Low groups according to their TOMM34 expression levels. Statistical difference was determined using log-rank (Mantel-Cox) test.

Data information: Data are presented as means ± SD.
Source data are available online for this figure.

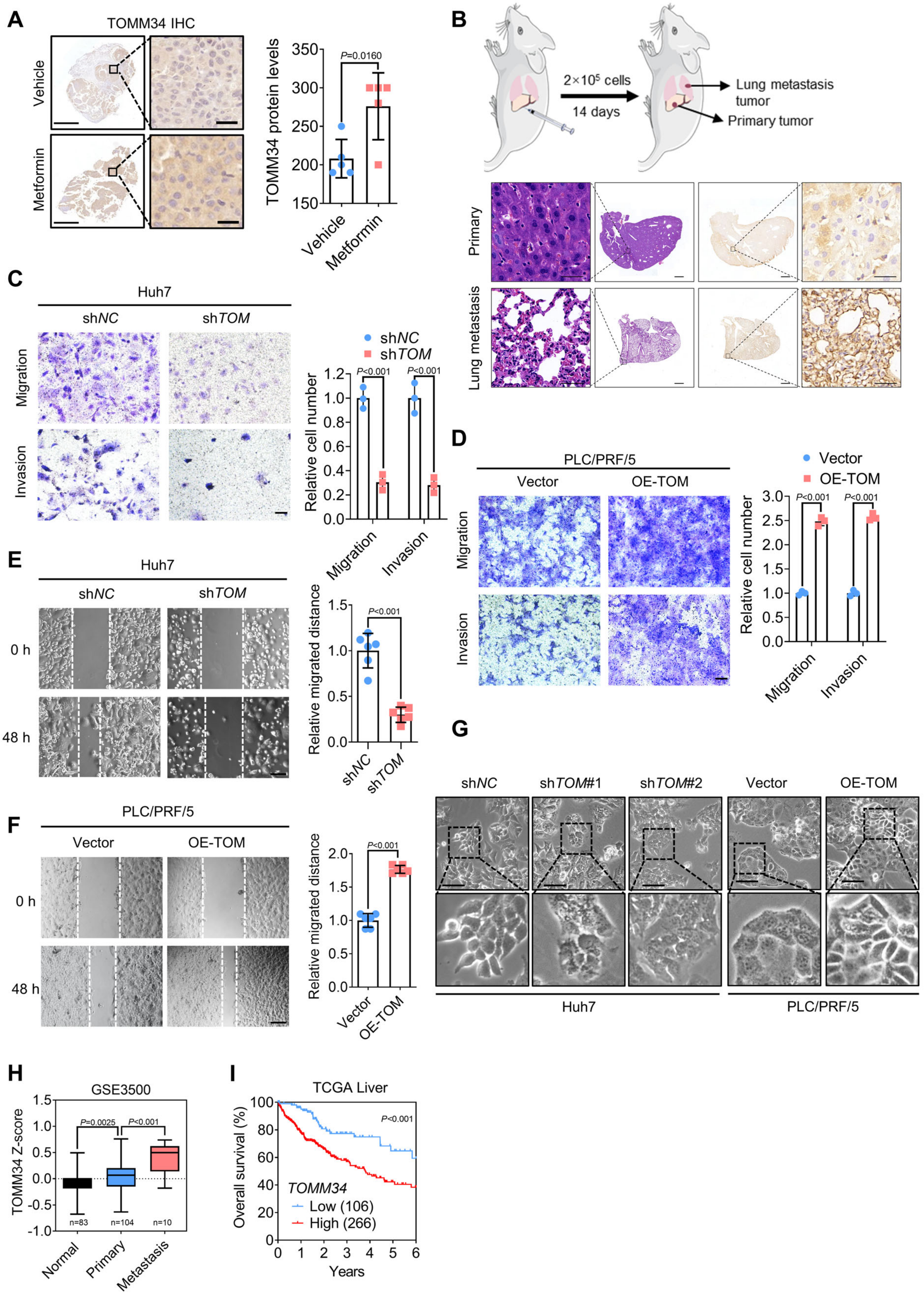

Figure EV2.

© 2022 The Authors

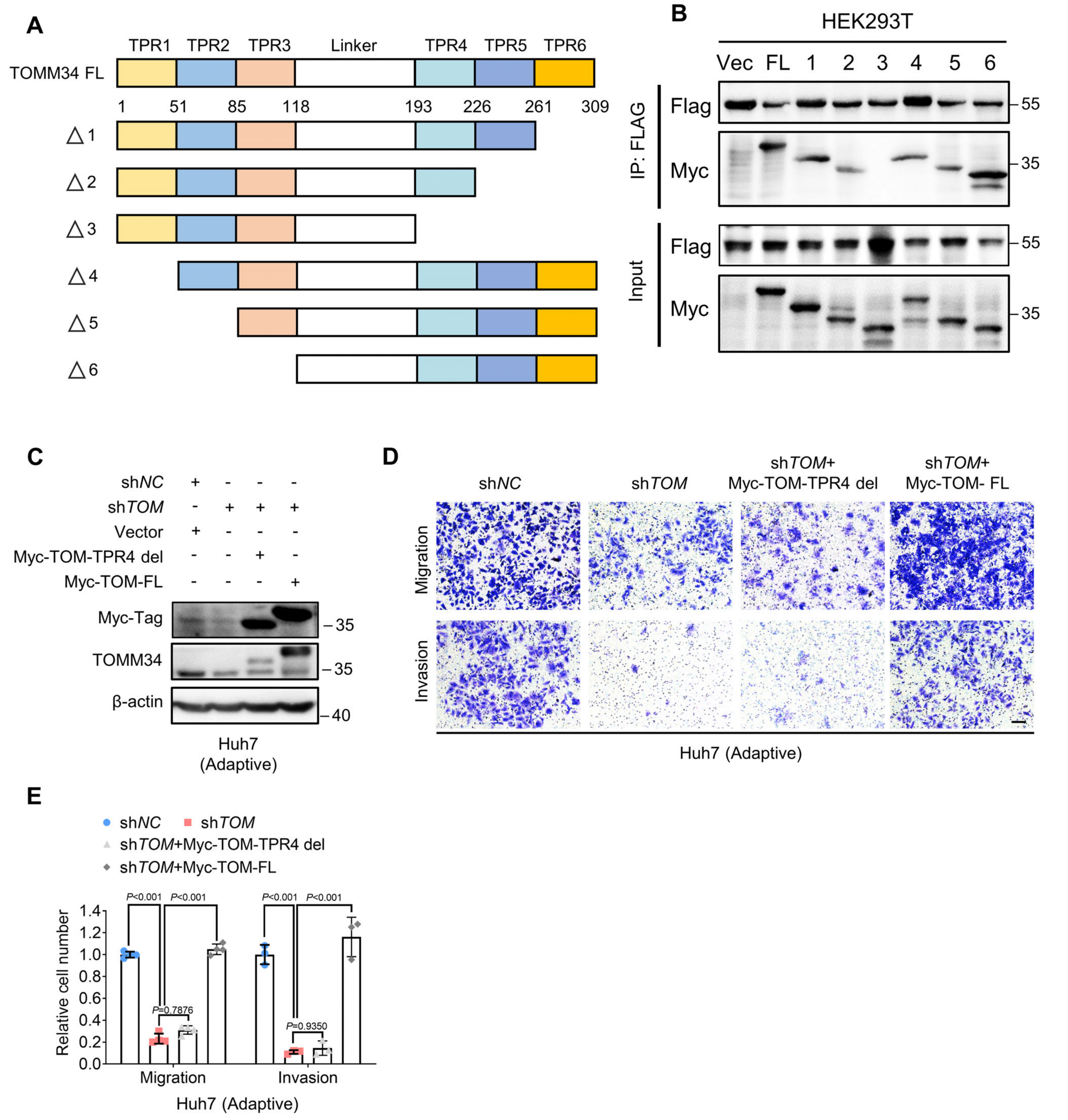

Figure EV3. TPR4 region is required for the interaction of TOMM34 with ATP5B.

A Schematic diagram showing wild-type TOMM34 (FL, full length, 1–309) and its truncations.

B HEK293T cells were transfected with the indicated plasmids, followed by co-IP assays and Western blot to examine their interactions with ATP5B (FL, full length, 1–309).

C Western blot showing the expression of full-length (Myc-TOM-FL) or TPR4-truncated TOMM34 (Myc-TOM-TPR4 del) in adaptive Huh7 sh*TOM* (sh*TOMM34*) cells.

D, E Transwell assays showing the migration and invasion of HCC cells expressing full-length (Myc-TOM-FL) or TPR4-truncated TOMM34 (Myc-TOM-TPR4 del) (5×10^4 cells). Scale bars, 100 μm. (n = 4 biological replicates for migration, n = 3 biological replicates for invasion, Two-way ANOVA).

Data information: Data are presented as means ± SD.
Source data are available online for this figure.

© 2022 The Authors

Figure EV4. ATP5B is essential for TOMM34-mediated metastasis.

A ATP levels in HCC cells were measured with or without silencing of ATP5B by siRNAs. ($n = 4$ biological replicates in Huh7, $n = 3$ biological replicates in PLC/PRF/5, One-way ANOVA).

B, C Western blot showing the expression of indicated proteins in HCC cells with or without ATP5B silencing (OE-TOM, OE-TOMM34, TOMM34-overexpressed cells).

D, E Transwell assays showing the migration and invasion of parental HCC cells with or without the silence of ATP5B (5×10^4 cells, OE-TOM, OE-TOMM34, TOMM34-overexpressed cells) Scale bars, 100 μm. ($n = 3$ biological replicates, Two-way ANOVA).

F, G Wound healing assay showing the migration of HCC cells transfected with si*Scramble* or si*ATP5B* (OE-TOM, OE-TOMM34, TOMM34-overexpressed cells) Scale bars, 200 μm. ($n = 3$ technical replicates, Two-way ANOVA).

Data information: Data are presented as means ± SD.
Source data are available online for this figure.

© 2022 The Authors

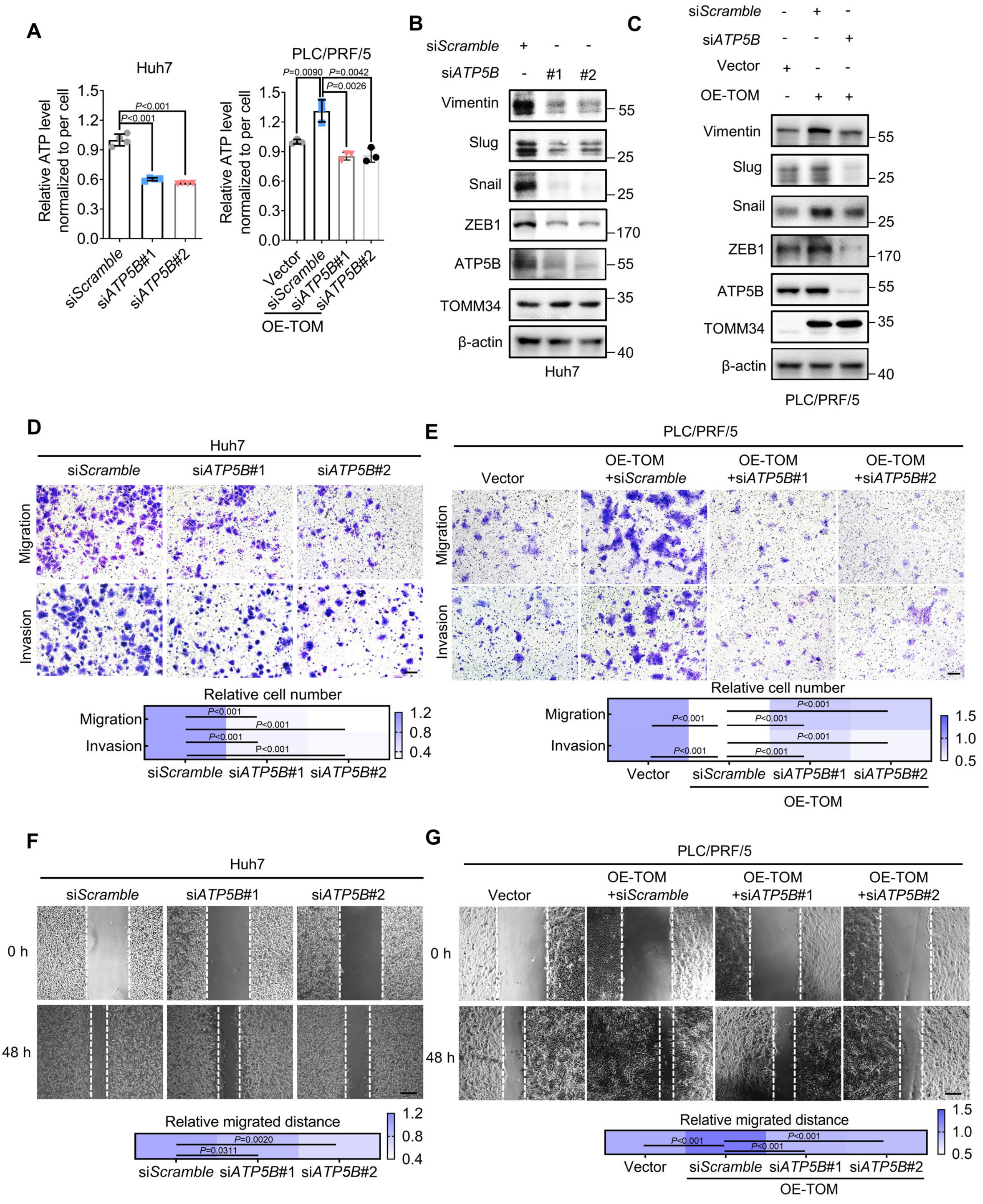
A
Huh7
PLC/PRF/5
Relative ATP level normalized to per cell
P<0.001
P<0.001
P=0.0090
P=0.0042
P=0.0026
siScramble
siATP5B#1
siATP5B#2
Vector
OE-TOM
B
siScramble
siATP5B
Vimentin
Slug
Snail
ZEB1
ATP5B
TOMM34
β-actin
Huh7
C
Vector
OE-TOM
PLC/PRF/5
D
Migration
Invasion
Relative cell number
E
OE-TOM +siScramble
OE-TOM +siATP5B#1
OE-TOM +siATP5B#2
F
0 h
48 h
Relative migrated distance
P=0.0020
P=0.0311
G

Figure EV4.

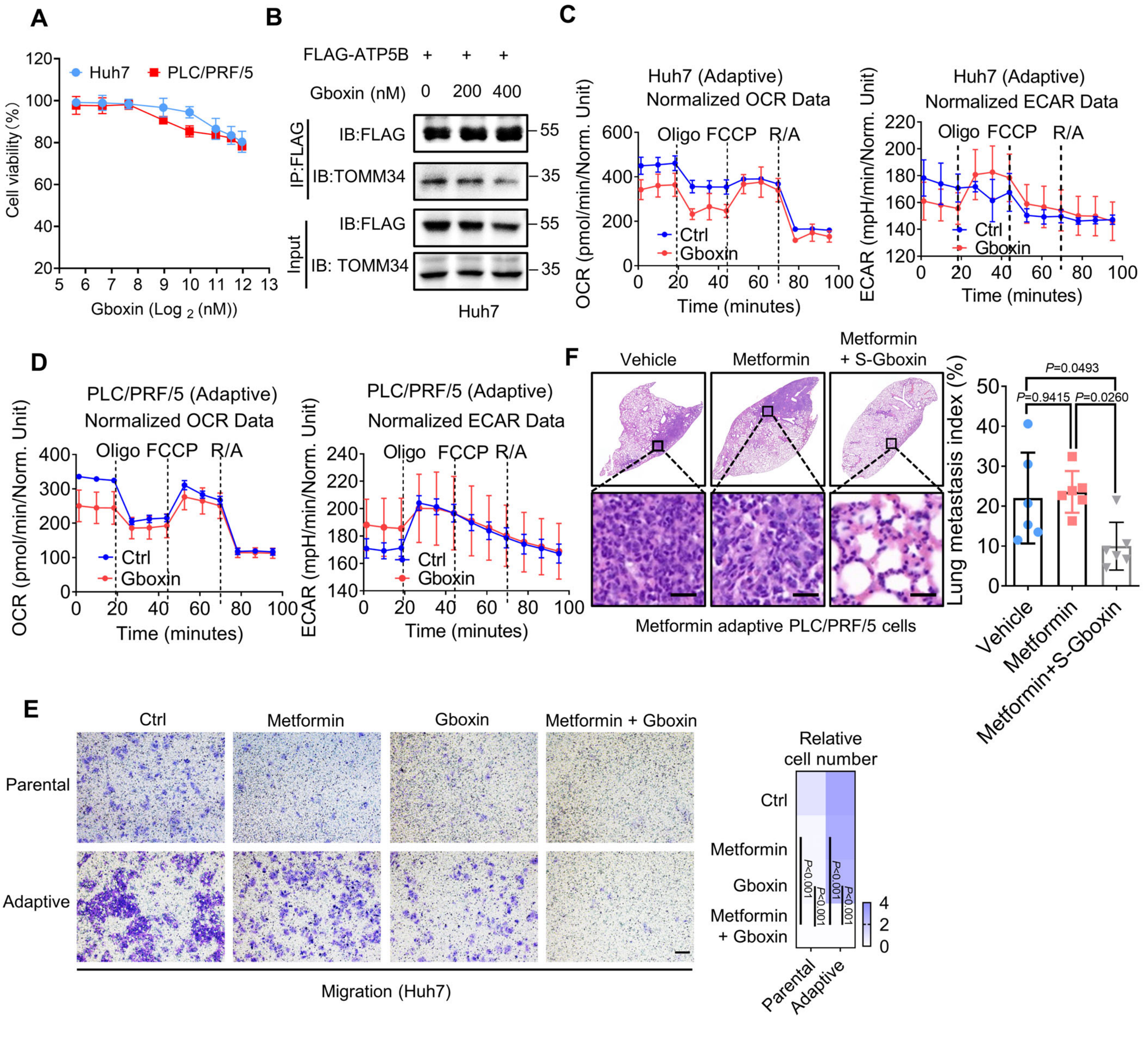

Figure EV5. Gboxin inhibits HCC metastasis by disturbing the interaction of TOMM34 and ATP5B.

A MTT assay showing the effects of Gboxin on the viability of HCC cells. (*n* = 6 biological replicates).

B Co-IP assay was performed to detect the interaction between TOMM34 and ATP5B treated with or without Gboxin for 24 h.

C, D The OCR and ECAR of indicated cells treated with or without Gboxin were measured using a Seahorse XF Analyzer (Oligo, oligomycin; FCCP, Carbonyl cyanide 4-(trifluoromethoxy)phenylhydrazone; R/A, rotenone/antimycin). (*n* = 3 biological replicates).

E Transwell assays showing the effect of combination treatment of Gboxin and metformin on the migration of parental and metformin-adaptive HCC cells (5×10^4 cells). Scale bars, 100 μm. (*n* = 3 biological replicates, Two-way ANOVA).

F A lung metastatic model showing migration of metformin-adaptive PLC/PRF/5 cells with or without Gboxin administration. Scale bars, 25 μm. (*n* = 6 mice for each group, Two-way ANOVA).

Data information: Data are presented as means ± SD.
Source data are available online for this figure.

 © 2022 The Authors
