# Supplementary figures and images for: Disrupting metformin adaptation of liver cancer cells by targeting the TOMM34/ATP5B axis
Source: EMBO Mol Med. 2022 Nov 2;14(12):e16082. doi: 10.15252/emmm.202216082 (PMC9728056; doi:10.15252/emmm.202216082)

Appendix Fig. S3

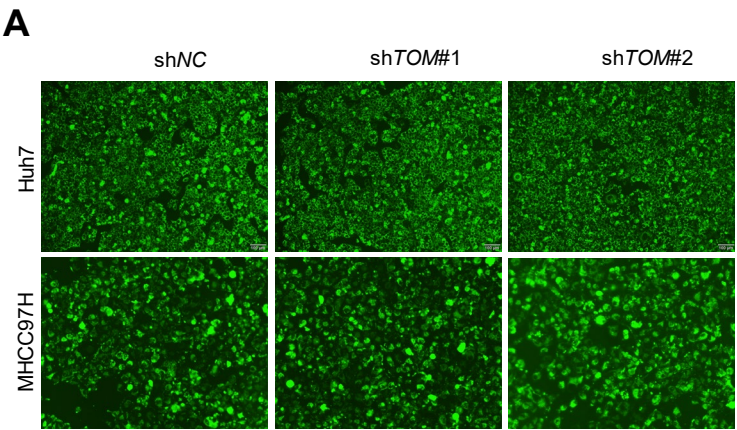

Supplement: Supplementary file 3 — Source Data for Expanded View and Appendix [file EMMM-14-e16082-s008.zip › EMM-2022-16082_Source Data for Expanded View and Appendix content/EMM-2022-1608_SourceDataFoAppendix Fig.S3A.pdf]

Appendix Fig. S1

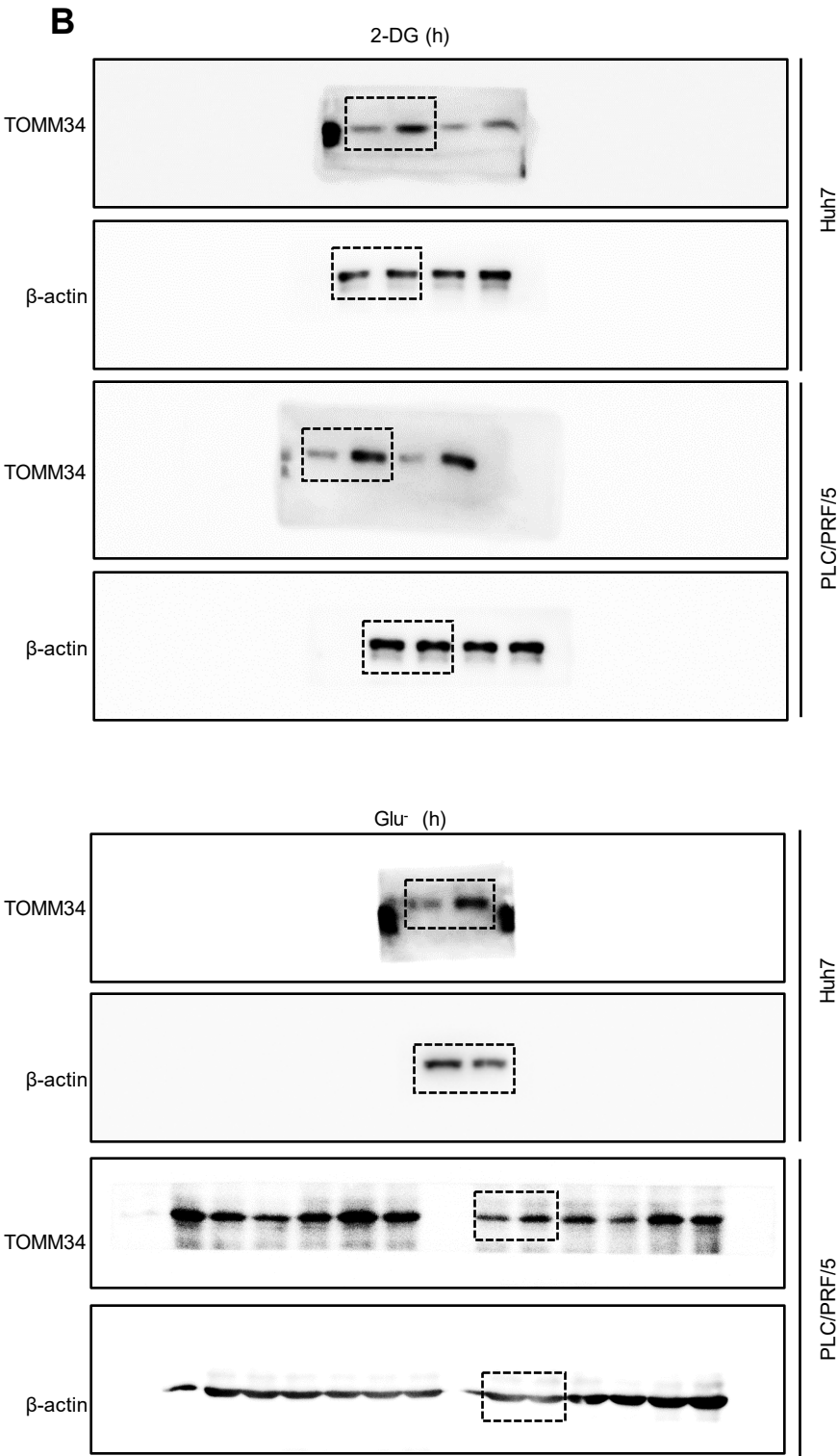

Appendix Fig. S1

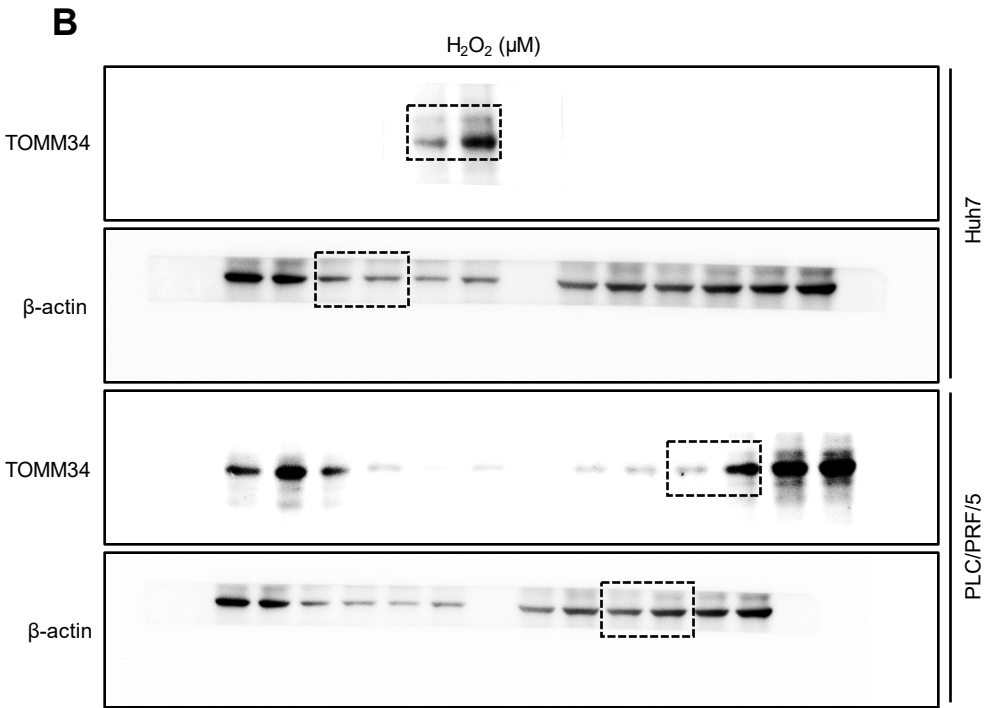

Supplement: Supplementary file 3 — Source Data for Expanded View and Appendix [file EMMM-14-e16082-s008.zip › EMM-2022-16082_Source Data for Expanded View and Appendix content/EMM-2022-1608_SourceDataForAppendix Fig.S1B.pdf]

Appendix Fig. S2

**A**

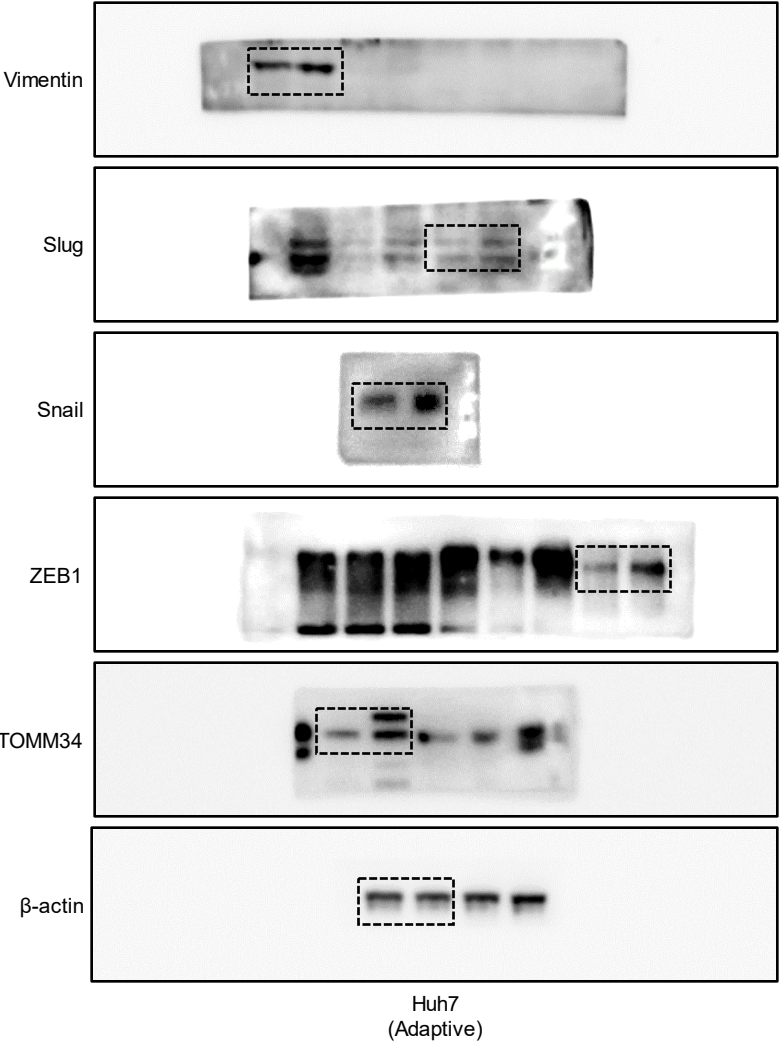

Appendix Fig. S2

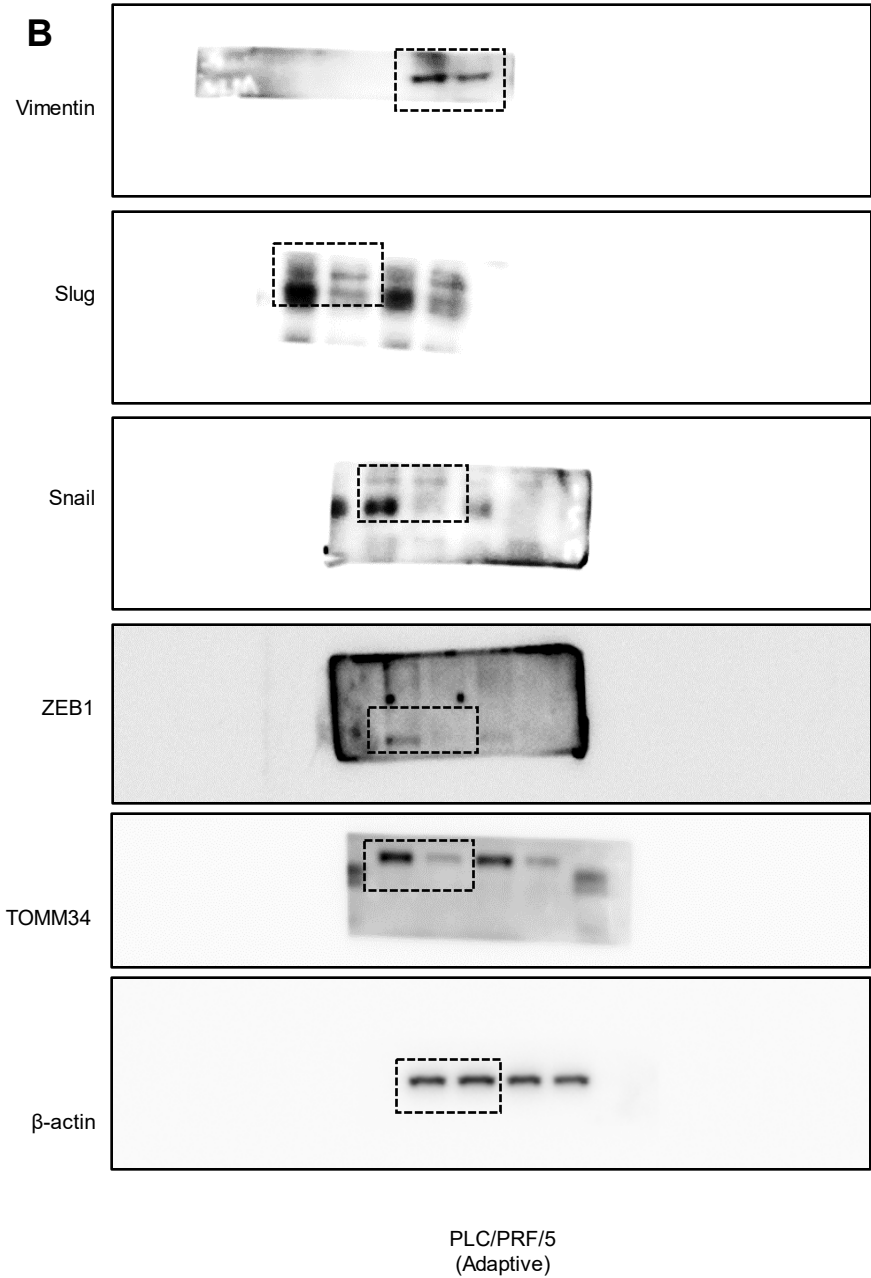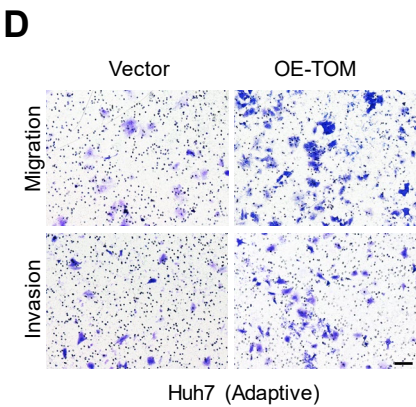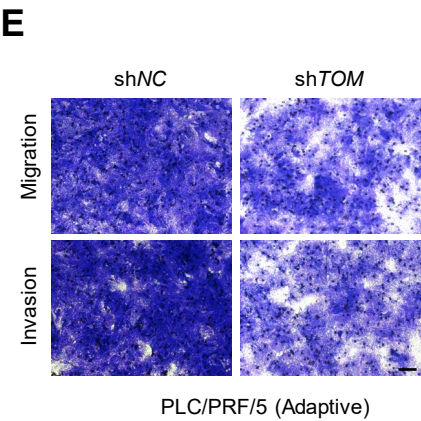

Supplement: Supplementary file 3 — Source Data for Expanded View and Appendix [file EMMM-14-e16082-s008.zip › EMM-2022-16082_Source Data for Expanded View and Appendix content/EMM-2022-1608_SourceDataForAppendix Fig.S2A-B,D-E.pdf]

Appendix Fig. S5

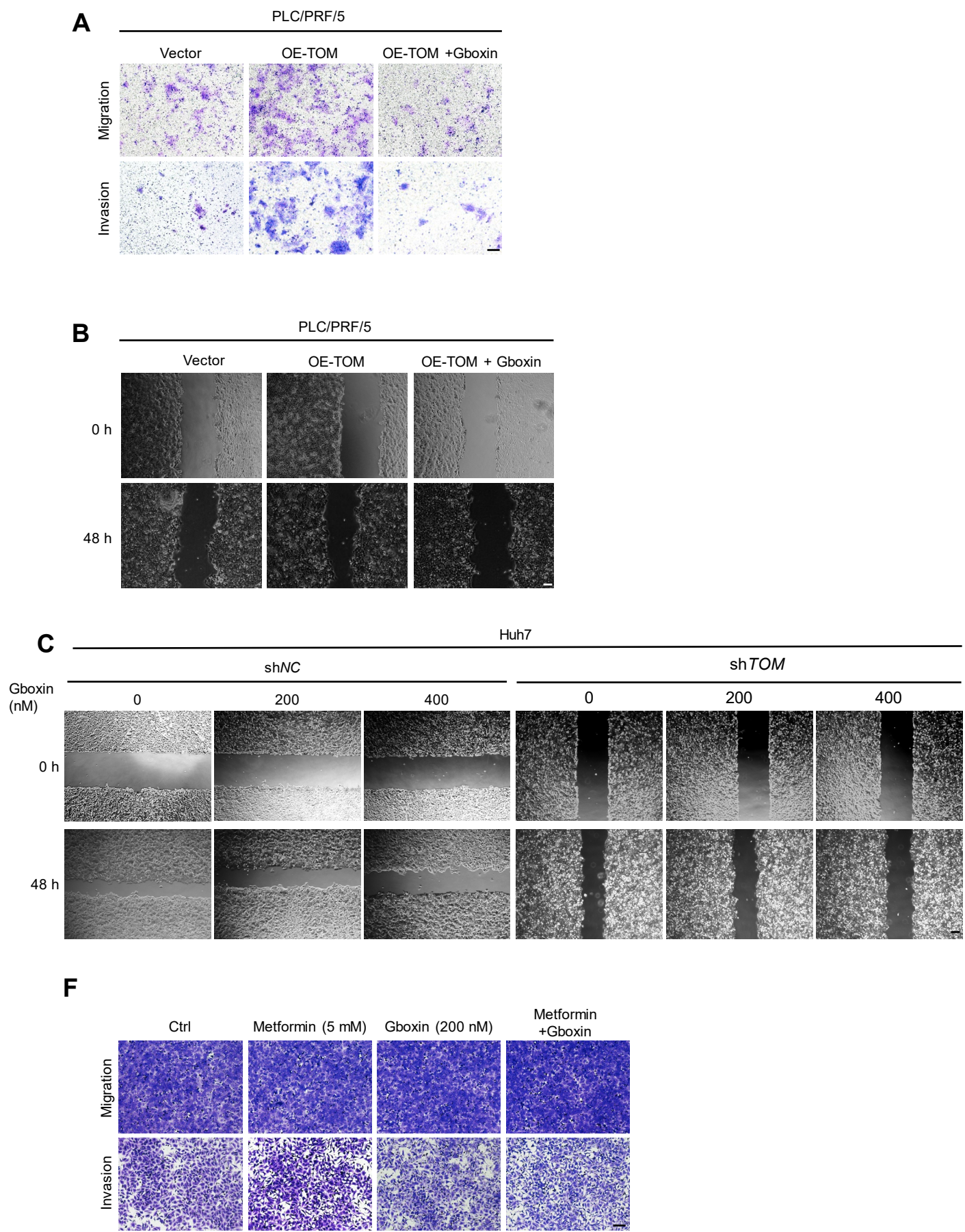

Supplement: Supplementary file 3 — Source Data for Expanded View and Appendix [file EMMM-14-e16082-s008.zip › EMM-2022-16082_Source Data for Expanded View and Appendix content/EMM-2022-1608_SourceDataForAppendix Fig.S5A-C,F.pdf]

Fig. EV2

**A**

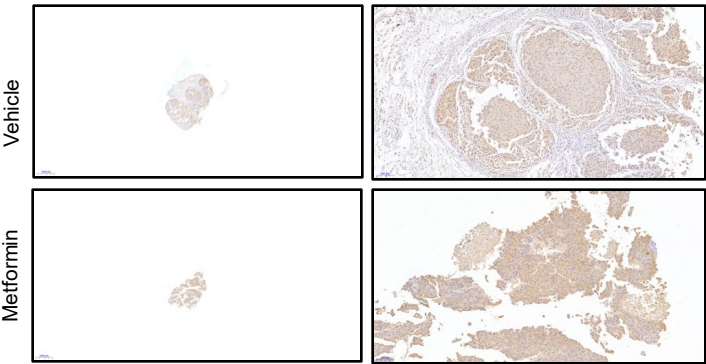

**B**

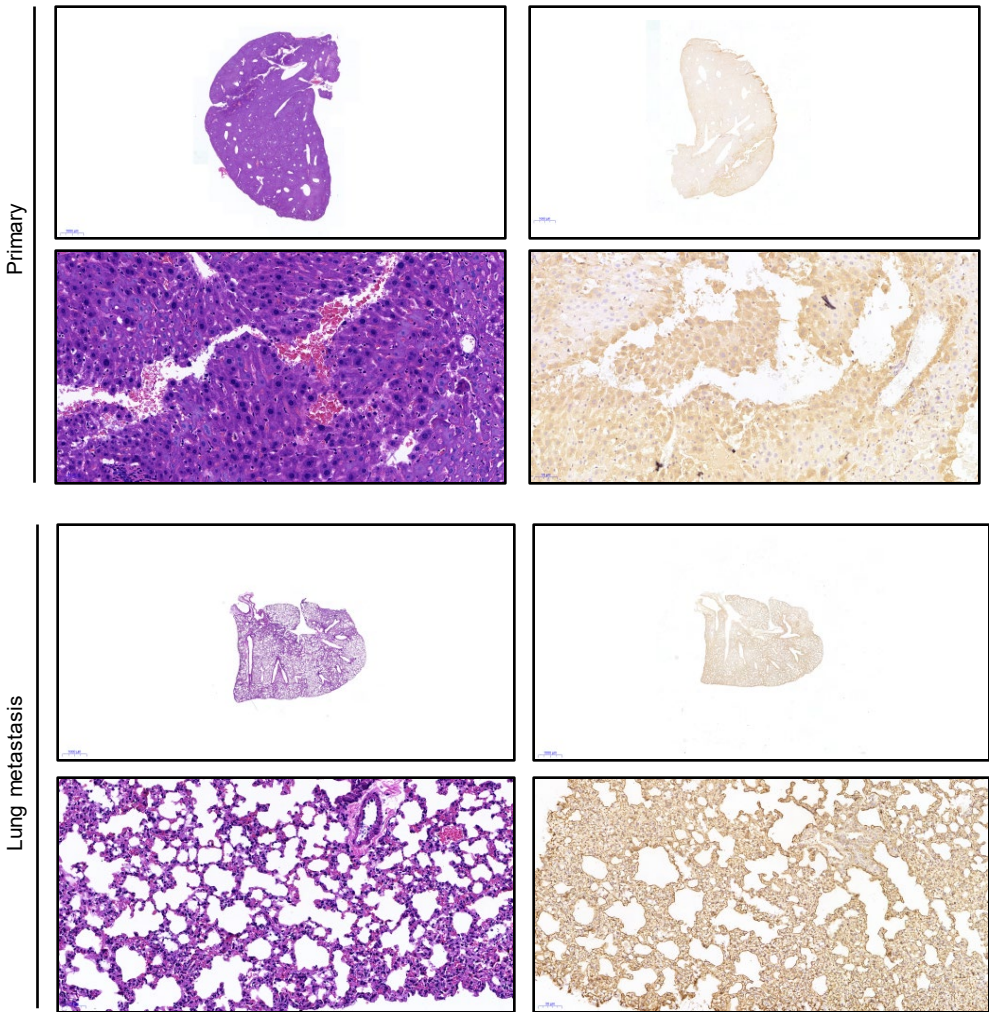

Fig. EV2

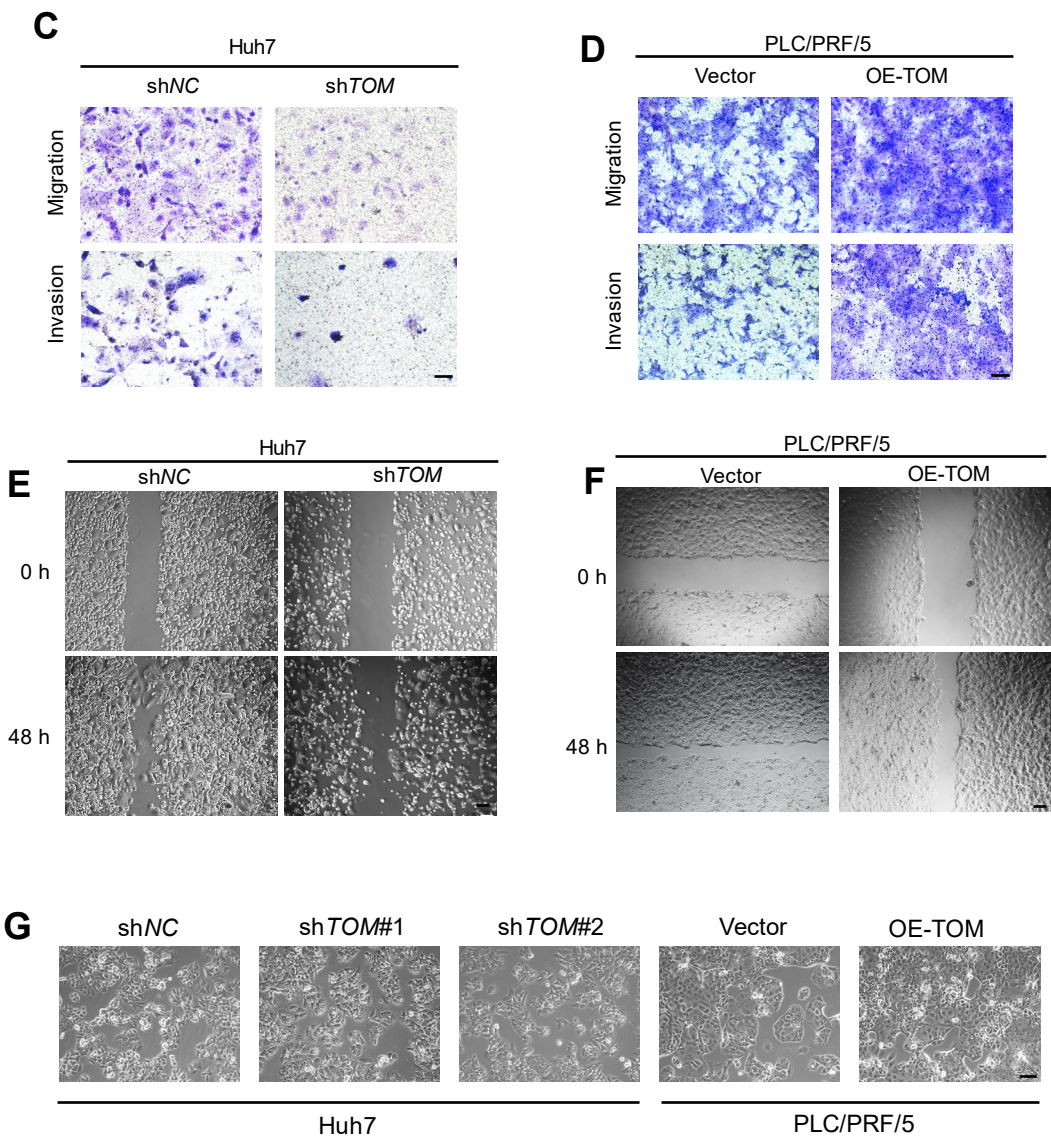

Supplement: Supplementary file 3 — Source Data for Expanded View and Appendix [file EMMM-14-e16082-s008.zip › EMM-2022-16082_Source Data for Expanded View and Appendix content/EMM-2022-1608_SourceDataForFig. EV2A-G.pdf]

Fig. EV3

**B**

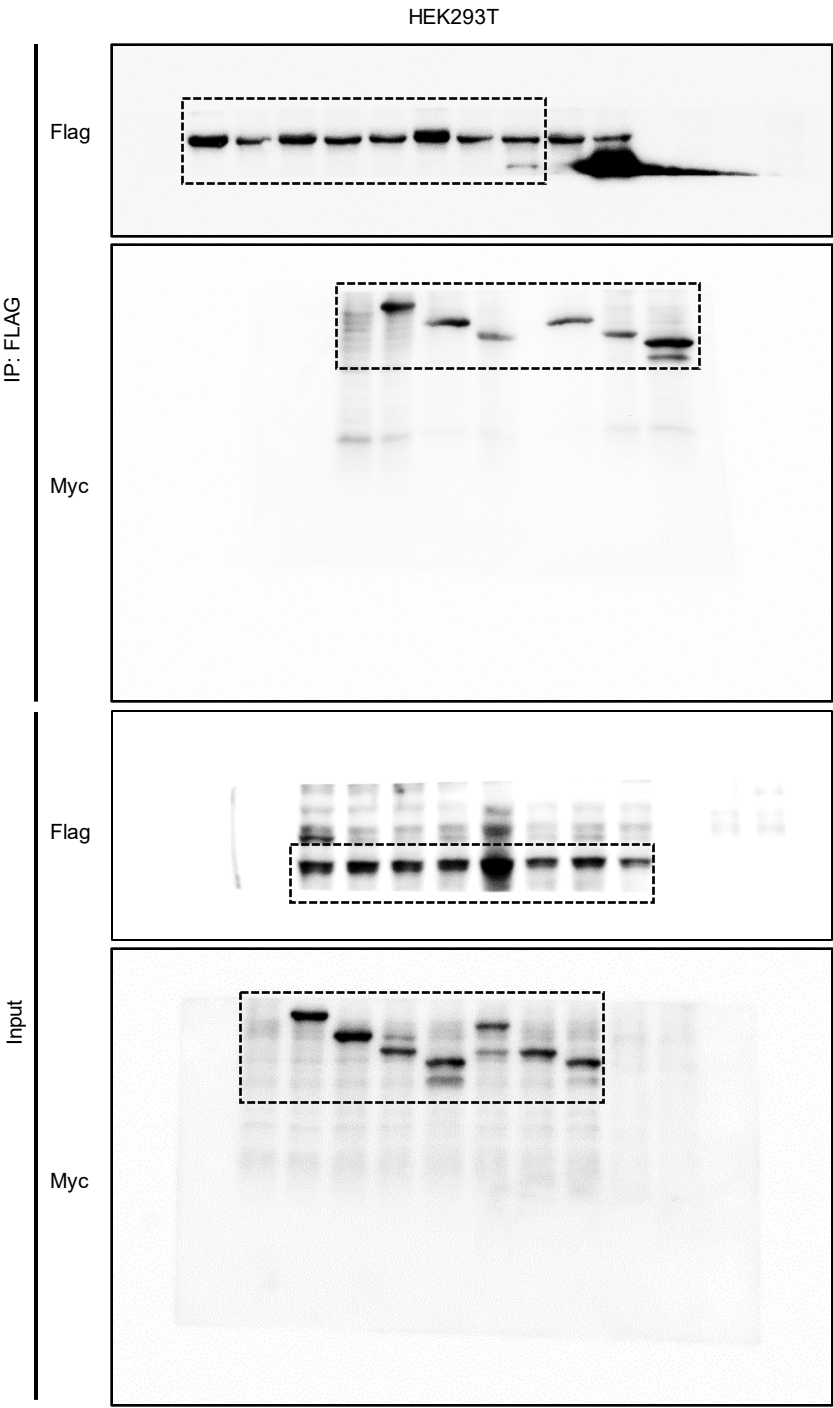

Fig. EV3

C

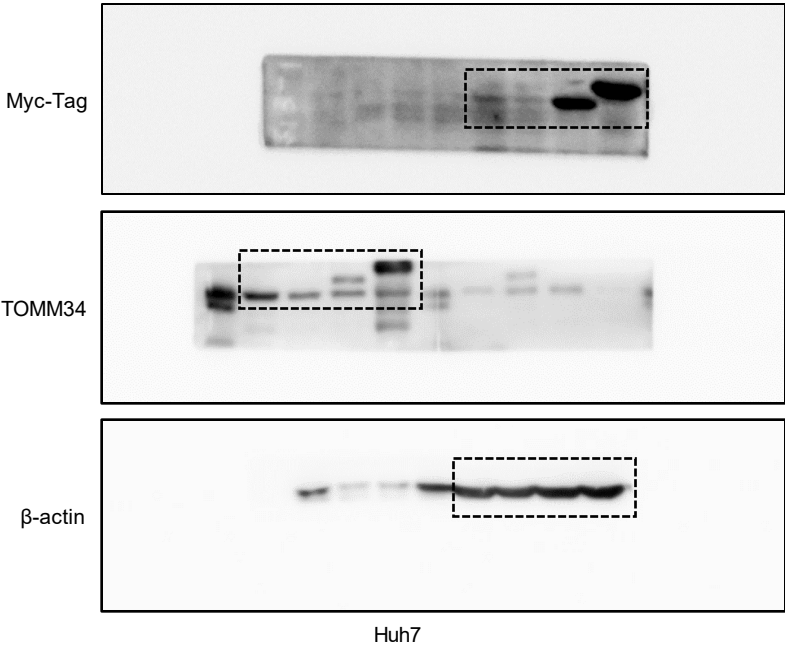

D

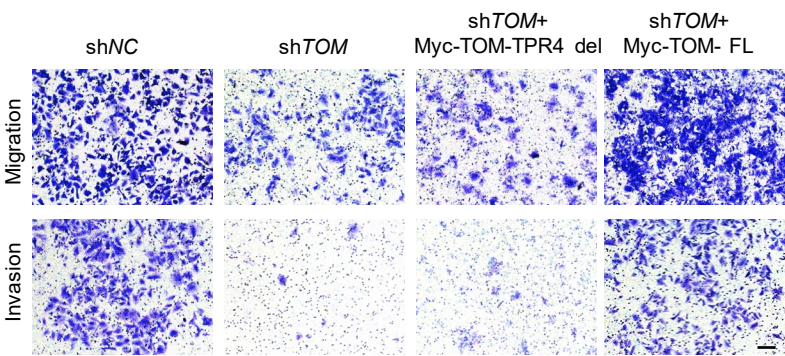

Supplement: Supplementary file 3 — Source Data for Expanded View and Appendix [file EMMM-14-e16082-s008.zip › EMM-2022-16082_Source Data for Expanded View and Appendix content/EMM-2022-1608_SourceDataForFig. EV3B-D.pdf]

Fig. EV4

**B**

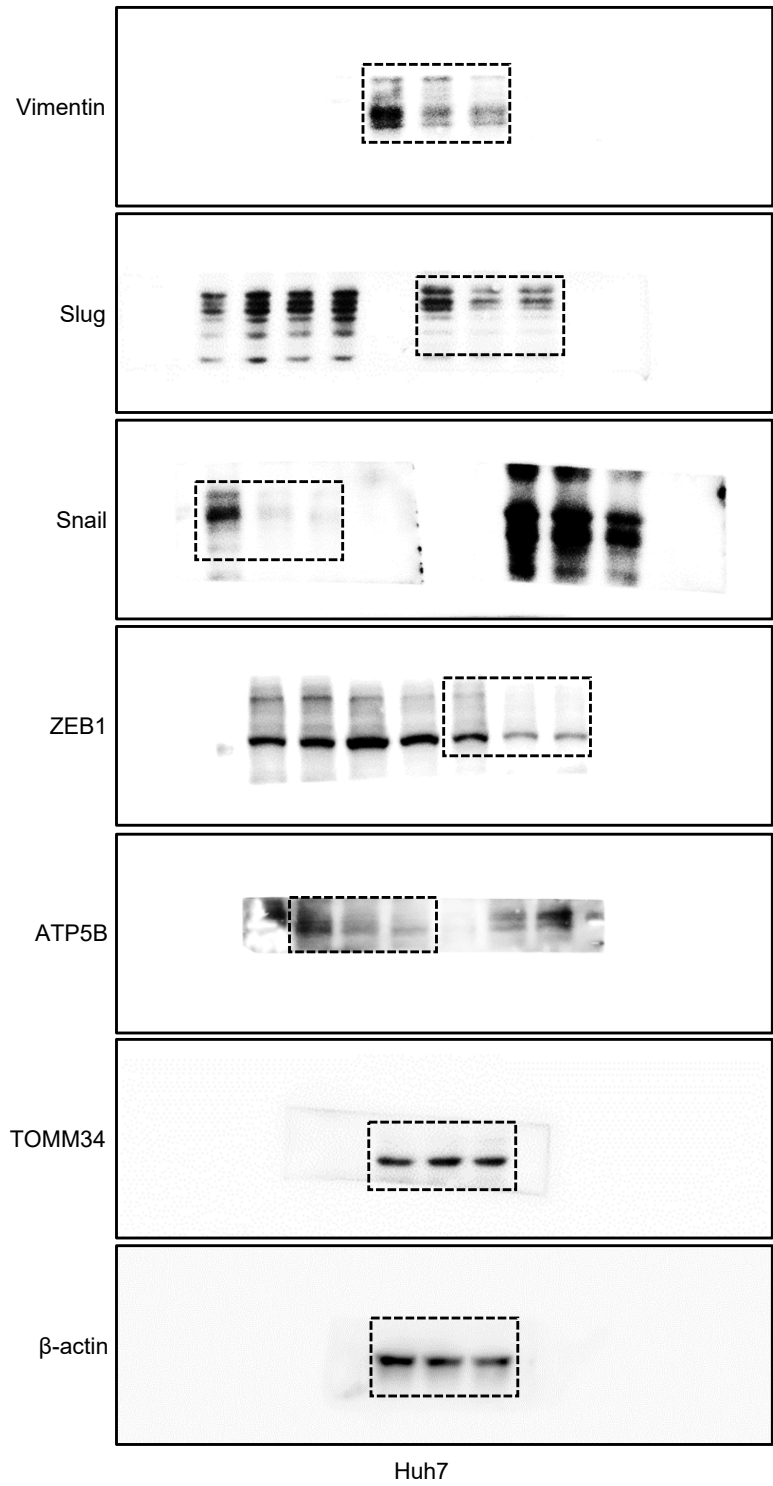

Fig. EV4

C

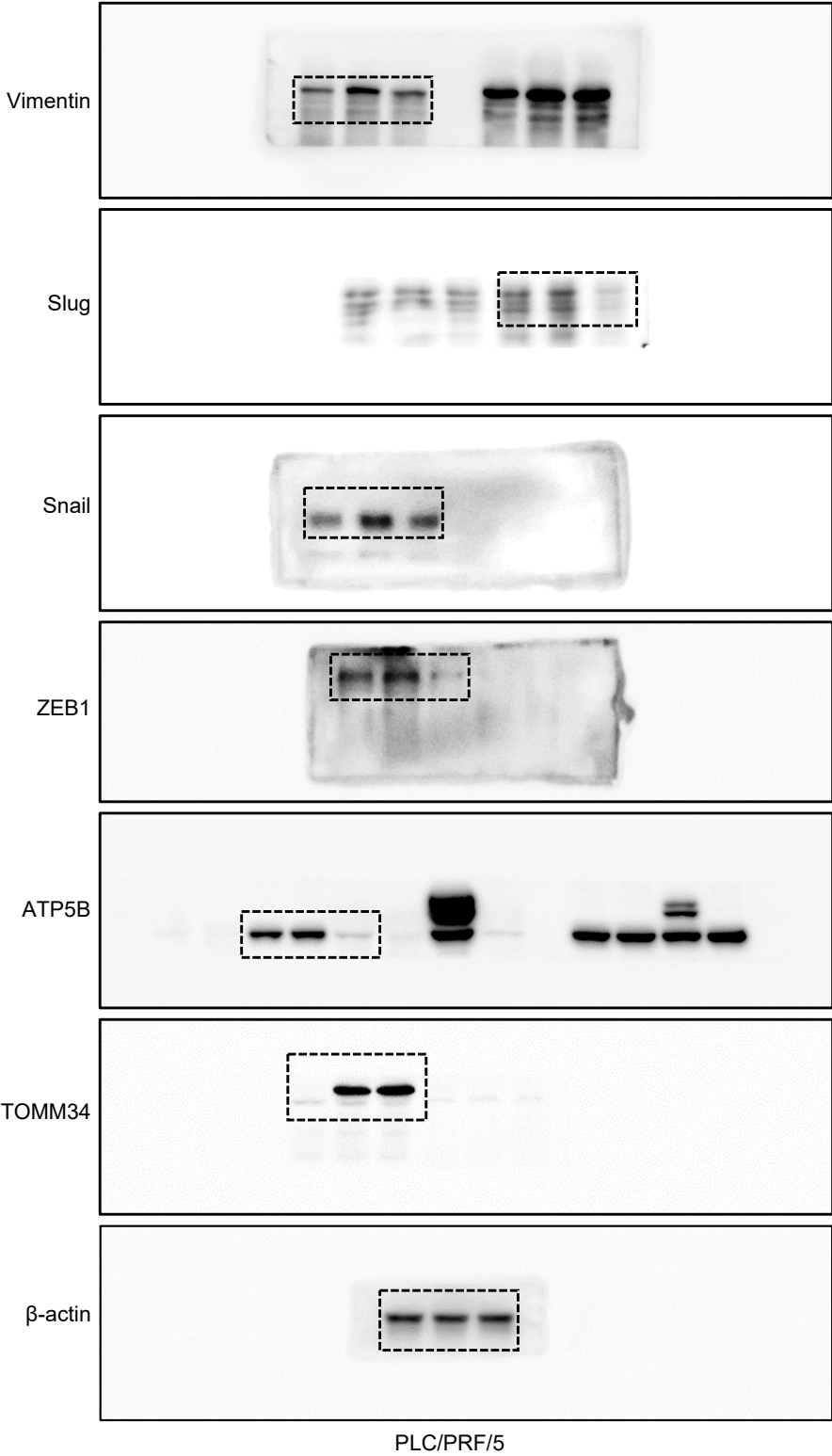

Fig. EV4

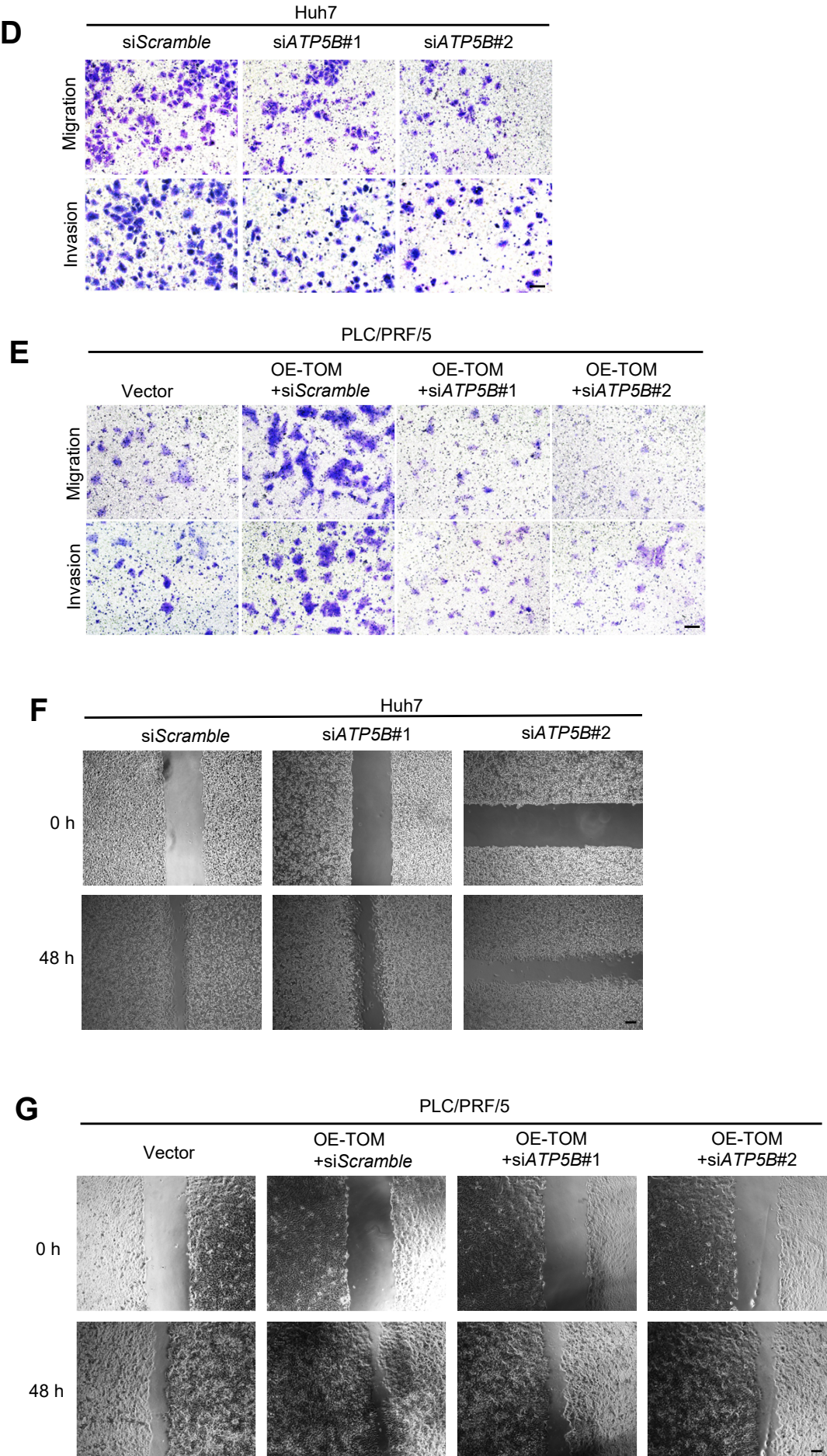

Supplement: Supplementary file 3 — Source Data for Expanded View and Appendix [file EMMM-14-e16082-s008.zip › EMM-2022-16082_Source Data for Expanded View and Appendix content/EMM-2022-1608_SourceDataForFig. EV4B-G.pdf]

Fig. EV5

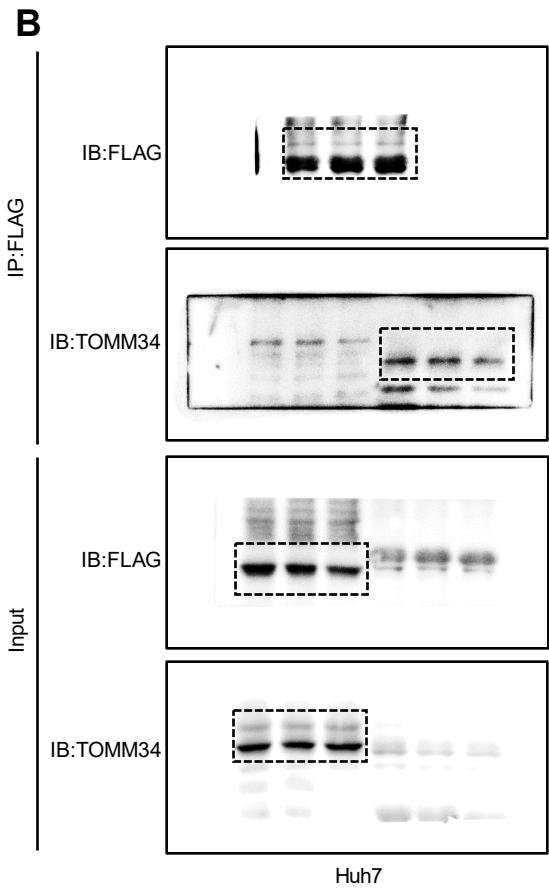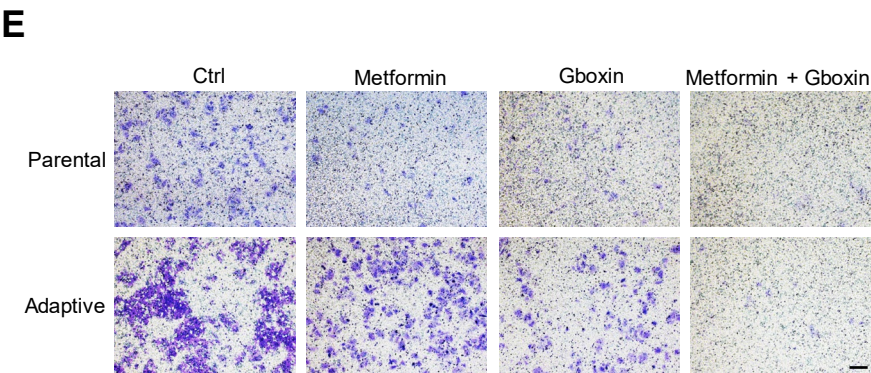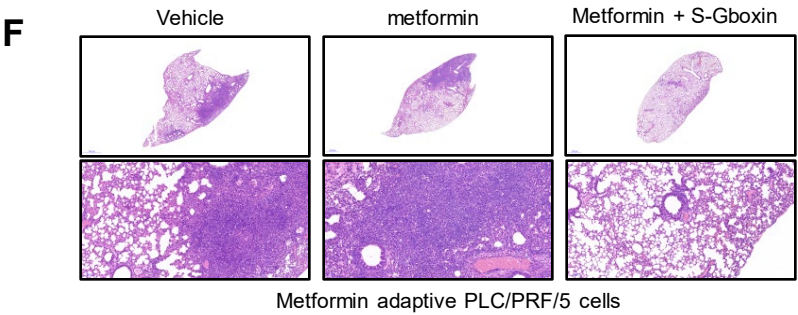

Supplement: Supplementary file 3 — Source Data for Expanded View and Appendix [file EMMM-14-e16082-s008.zip › EMM-2022-16082_Source Data for Expanded View and Appendix content/EMM-2022-1608_SourceDataForFig. EV5B,E-F.pdf]

Fig. 1

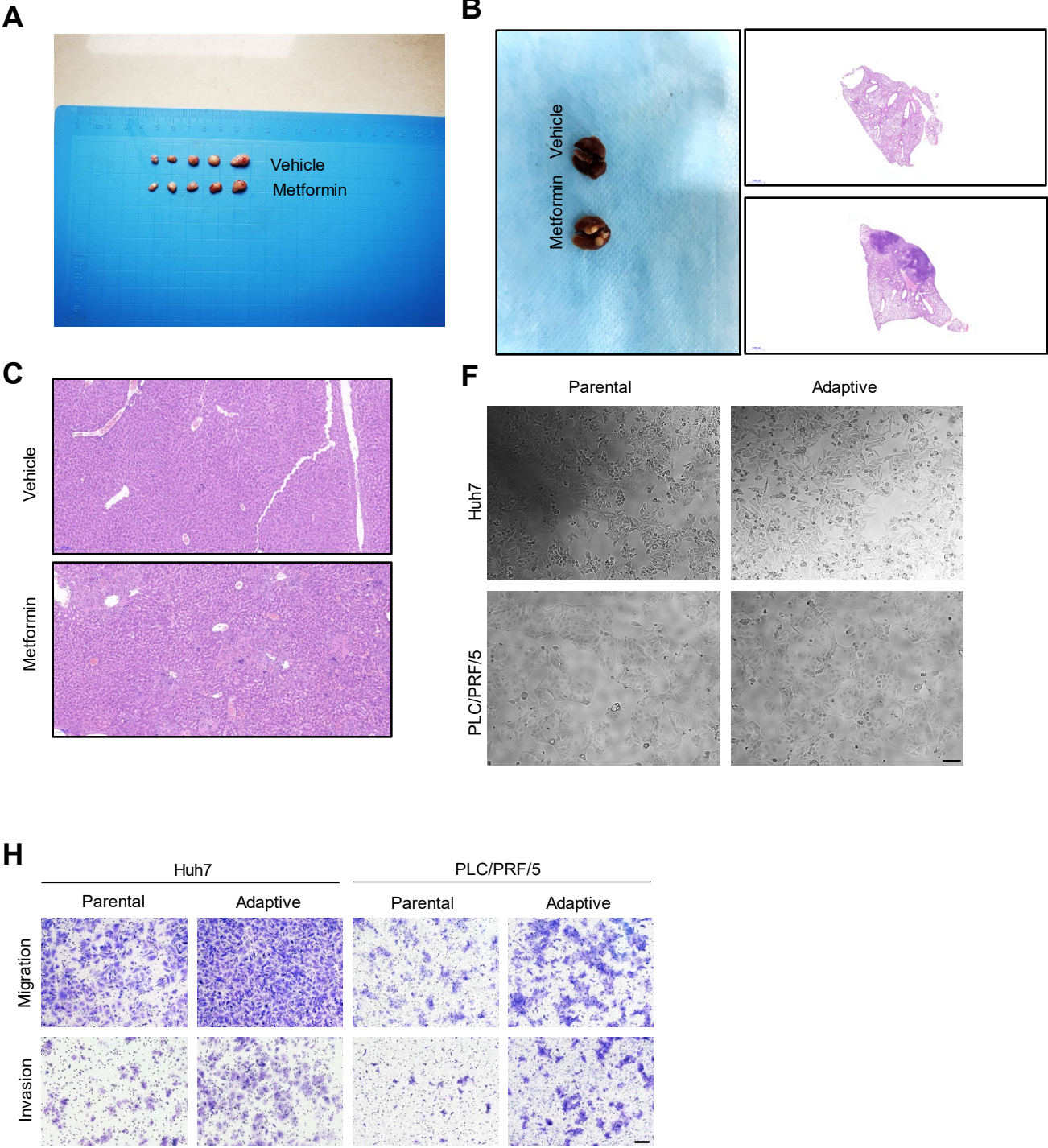

Supplement: Supplementary file 4 — Source Data for Figure 1 [file EMMM-14-e16082-s010.pdf]

Fig. 3

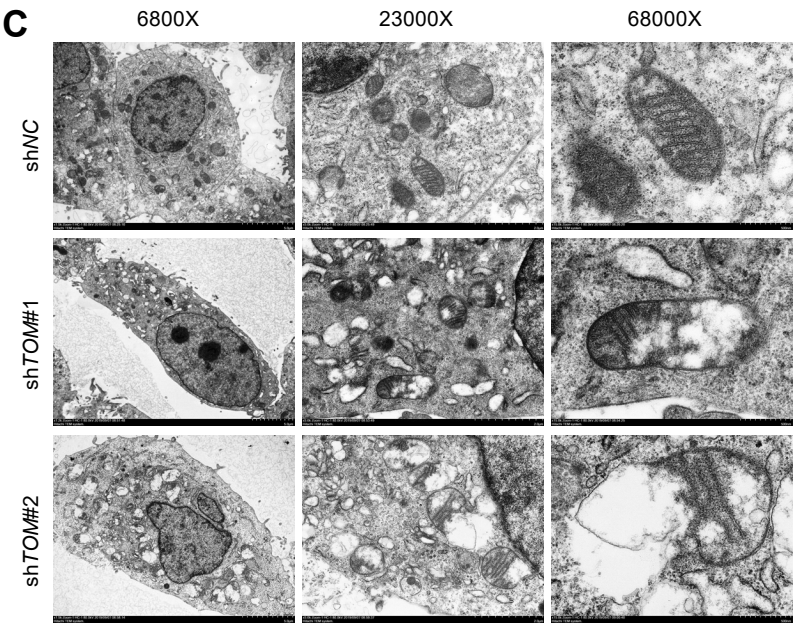

Fig. 3

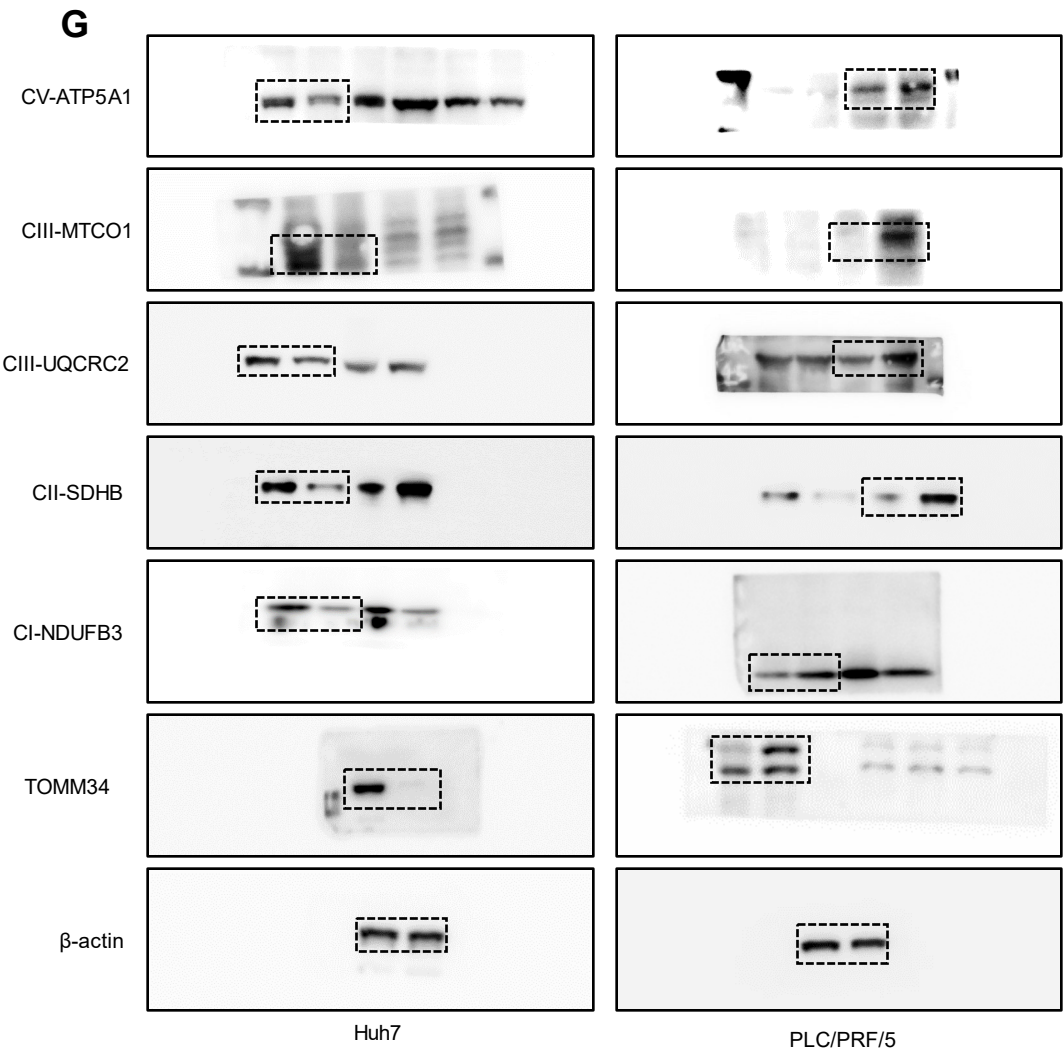

Supplement: Supplementary file 5 — Source Data for Figure 3 [file EMMM-14-e16082-s001.zip › EMM-2022-16082_SourceDataForFig.3/EMM-2022-16082_SourceDataForFig.3C,G.pdf]

Fig. 4

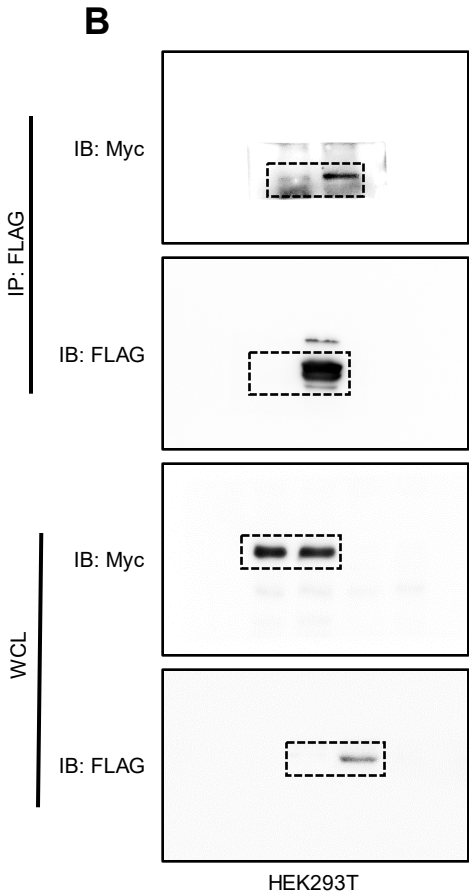

Fig. 4

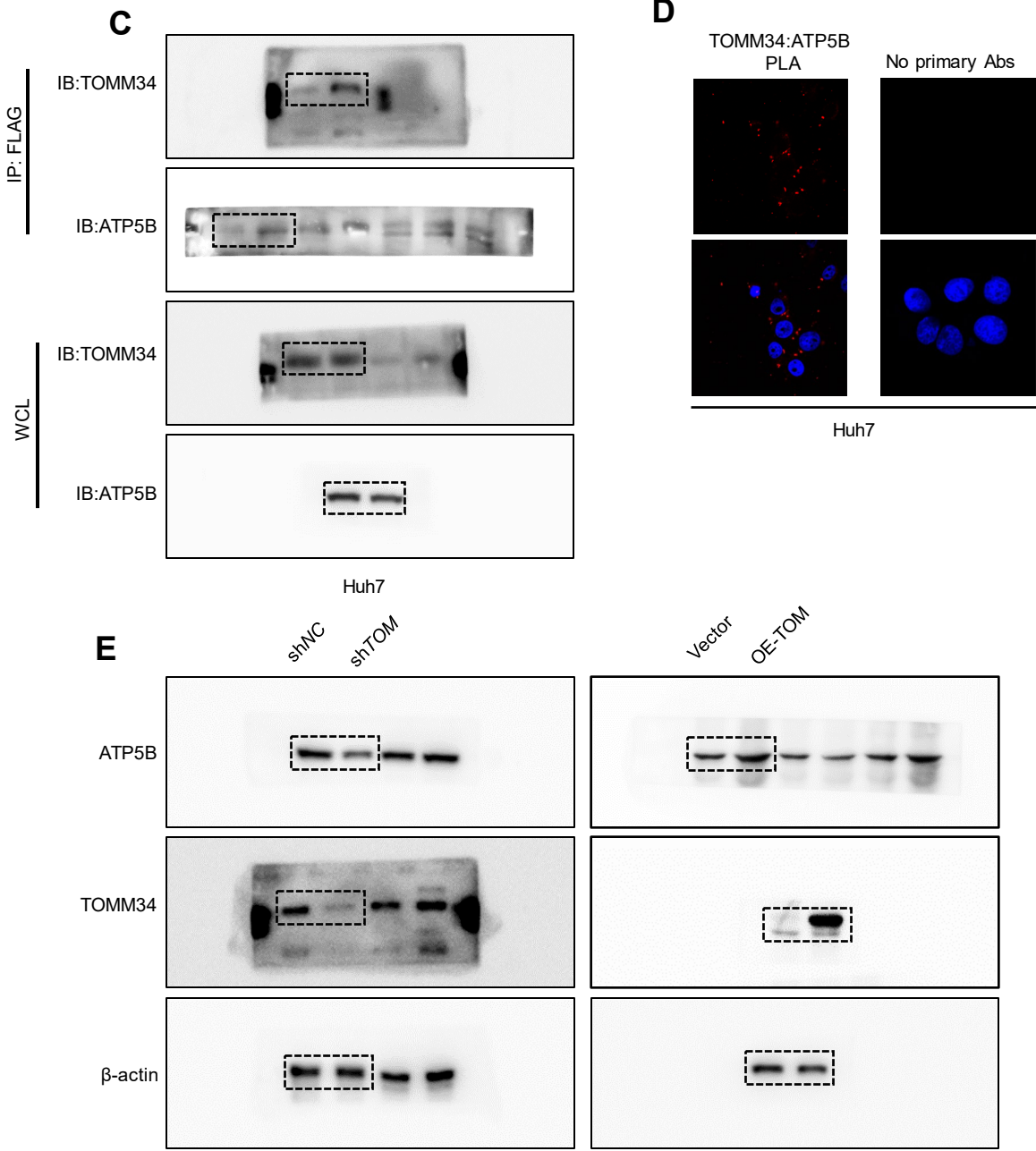

Fig. 4

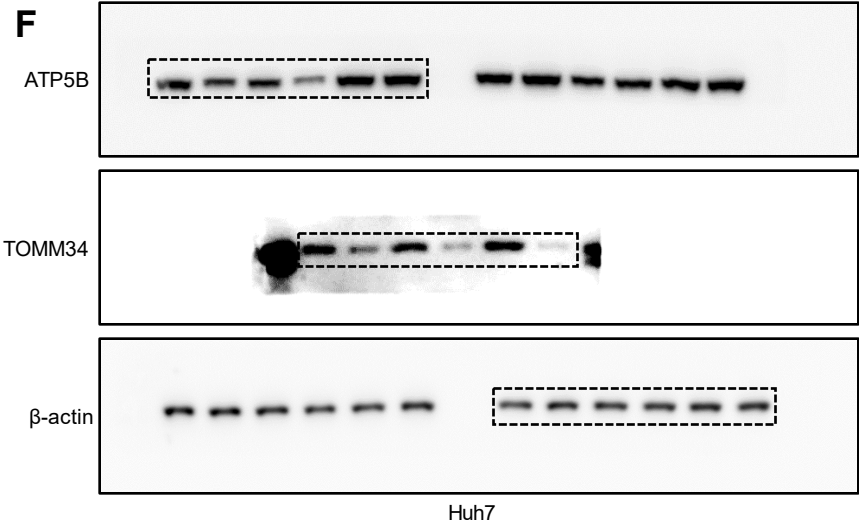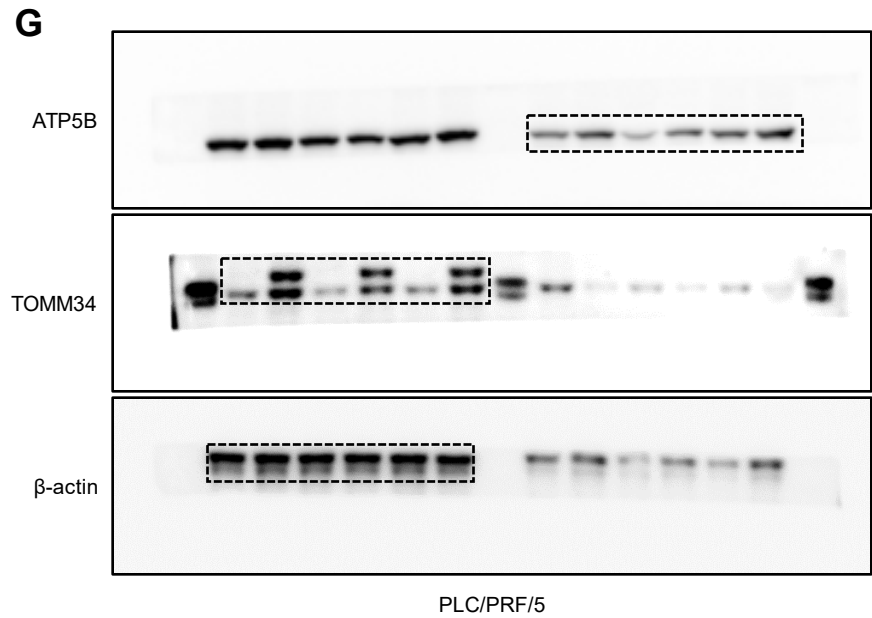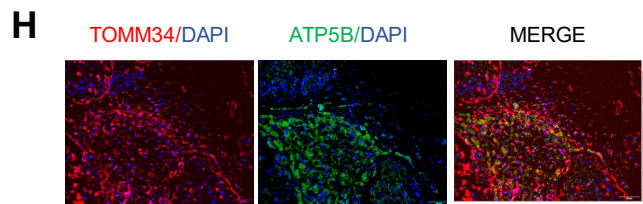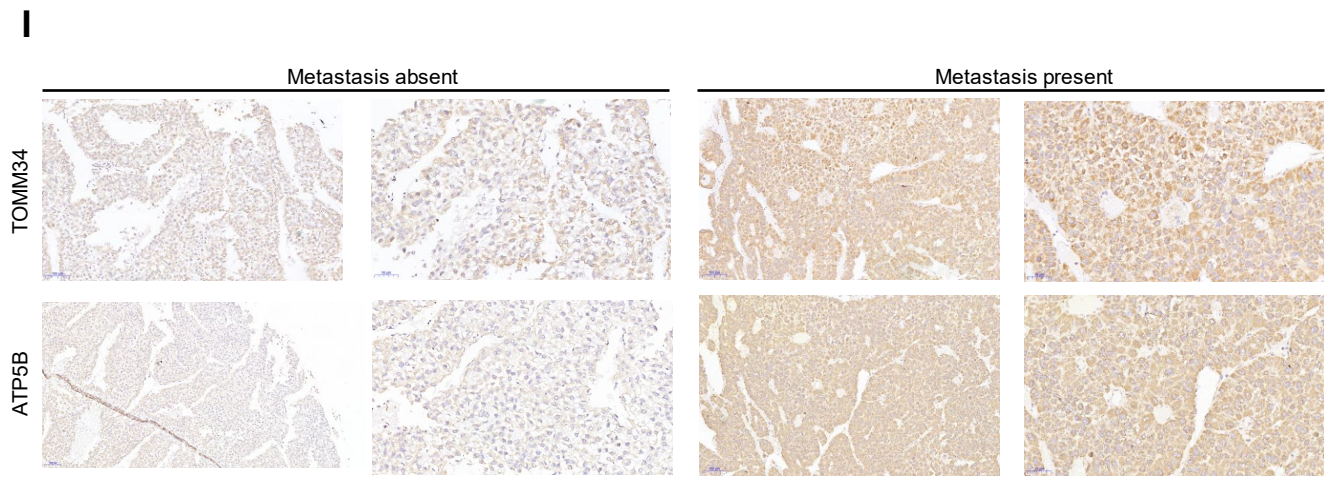

Supplement: Supplementary file 6 — Source Data for Figure 4 [file EMMM-14-e16082-s005.zip › EMM-2022-16082_SourceDataForFig.4/EMM-2022-16082_SourceDataForFig.4B-I.pdf]

Fig. 5

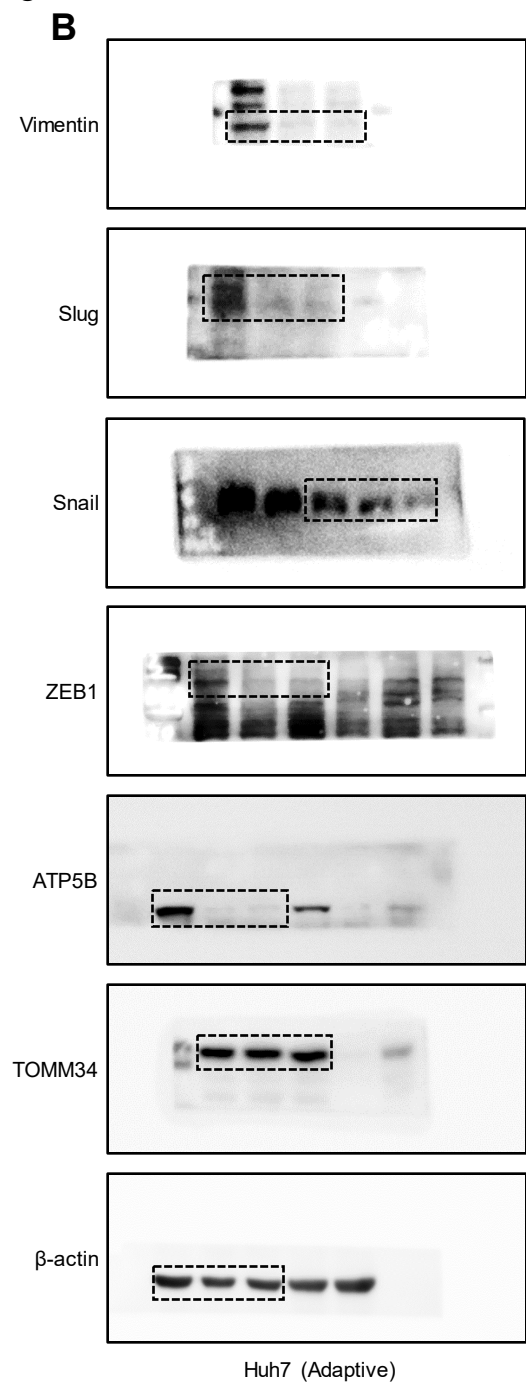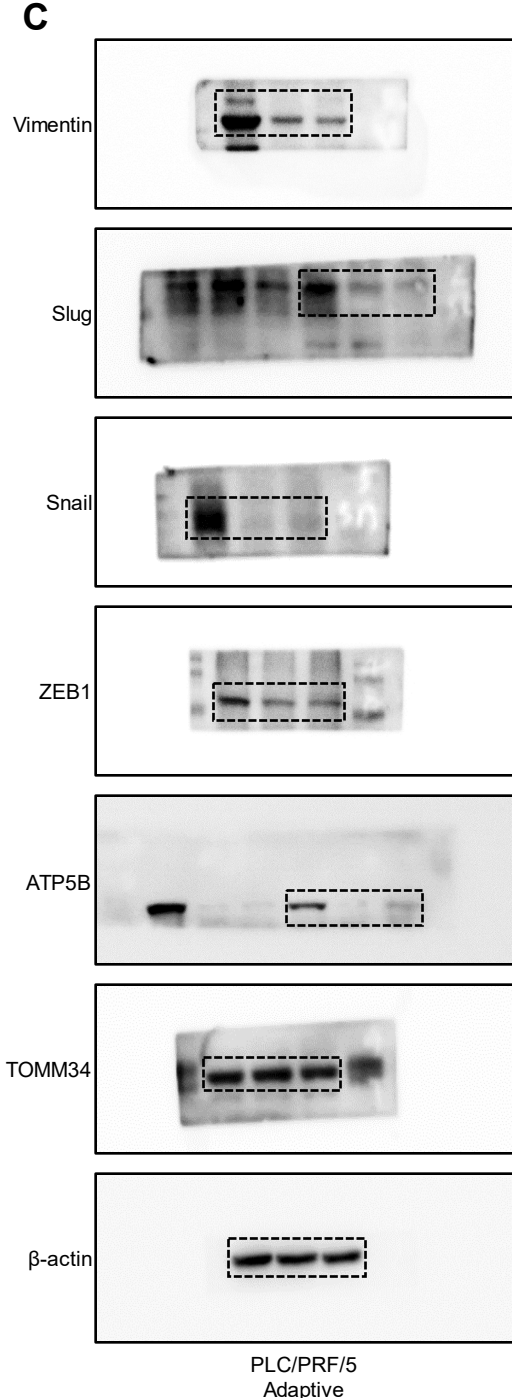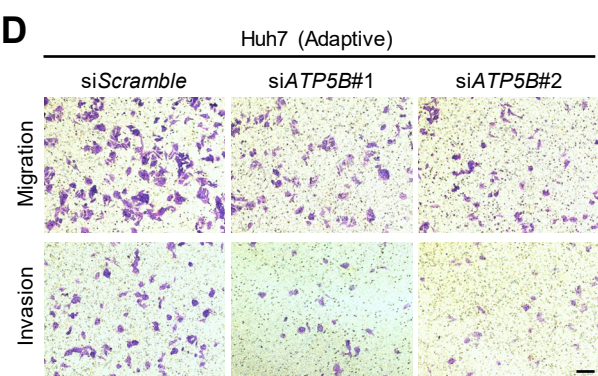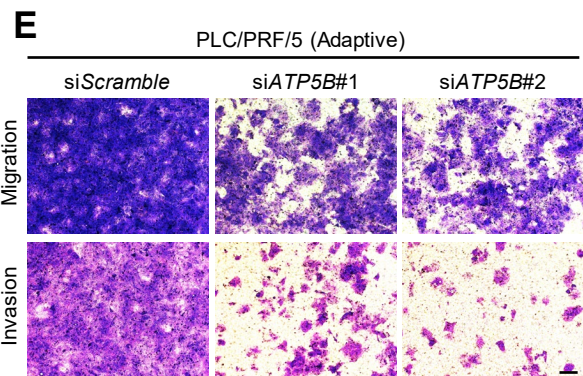

Fig. 5

**G**

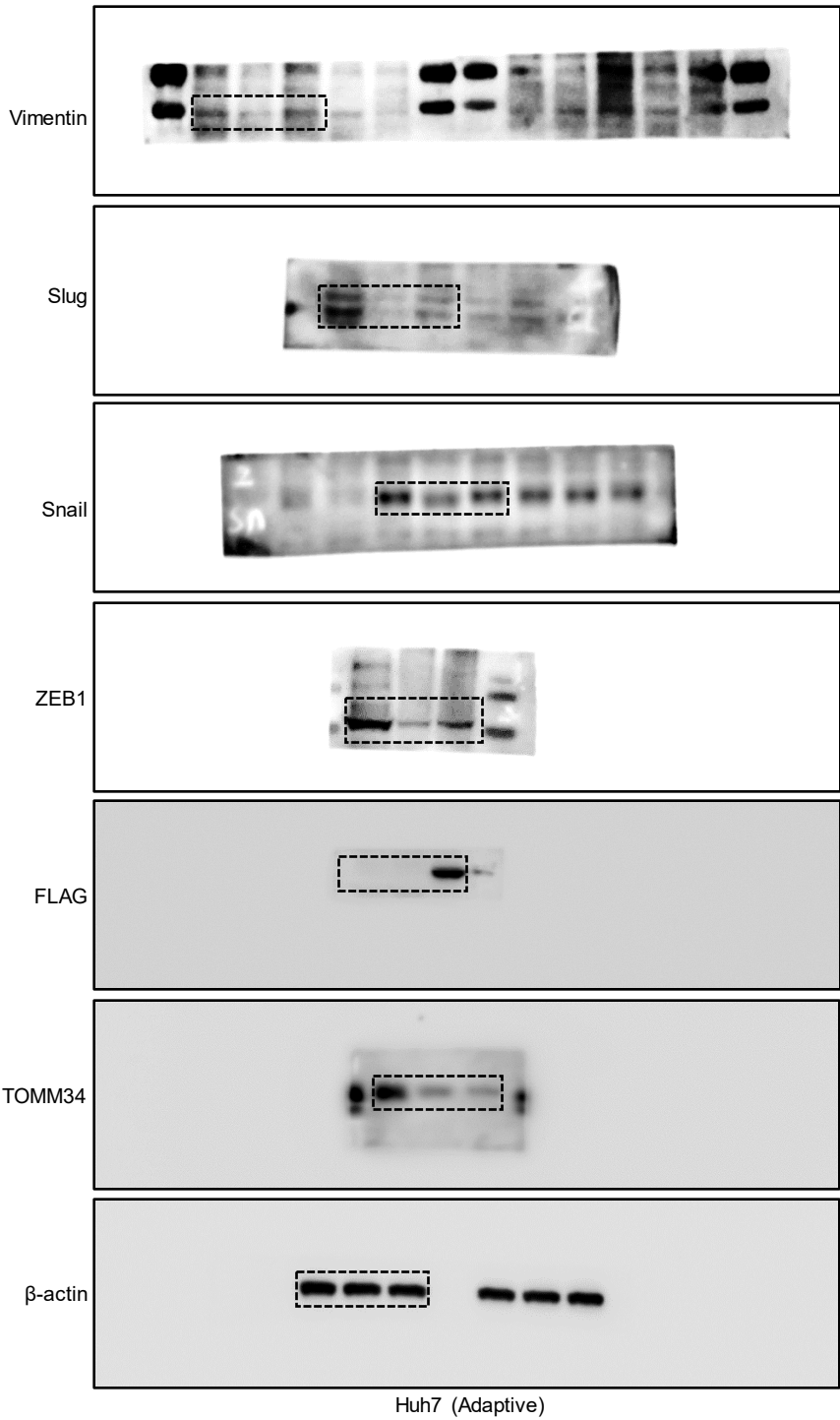

**H**

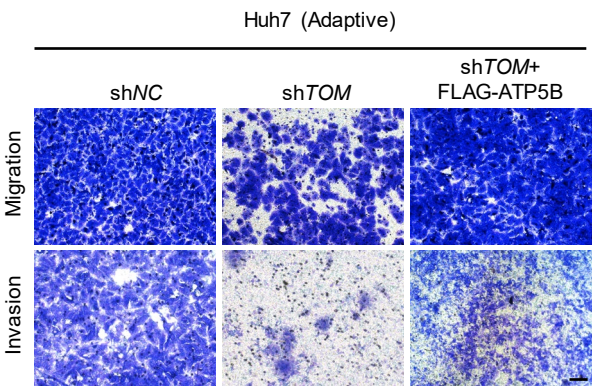

Supplement: Supplementary file 7 — Source Data for Figure 5 [file EMMM-14-e16082-s002.pdf]

Fig. 6

A

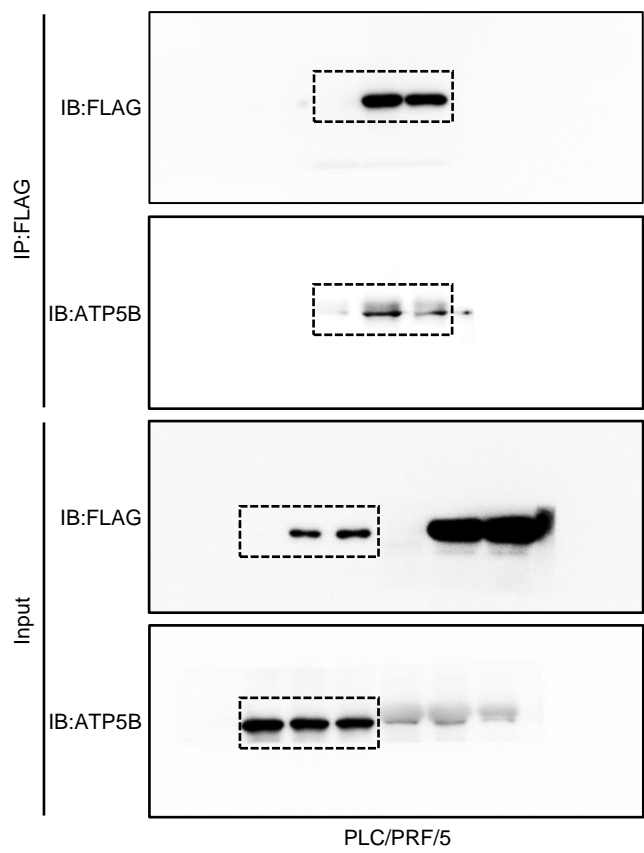

Fig. 6

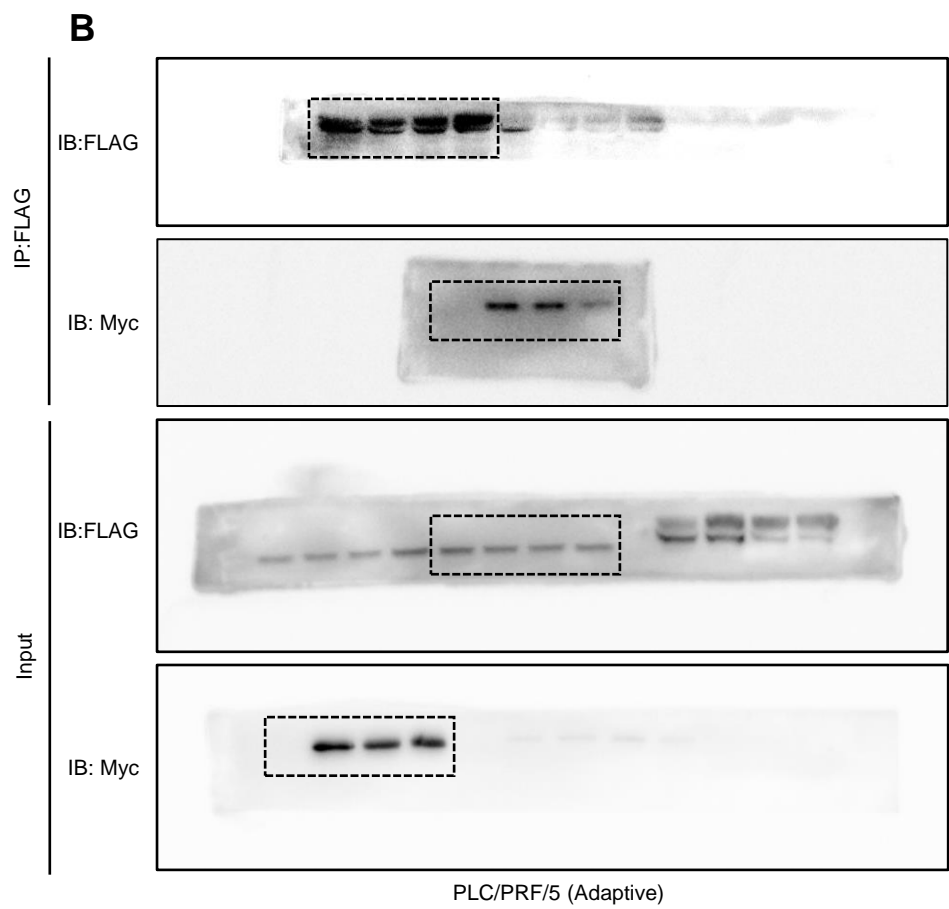

Fig. 6

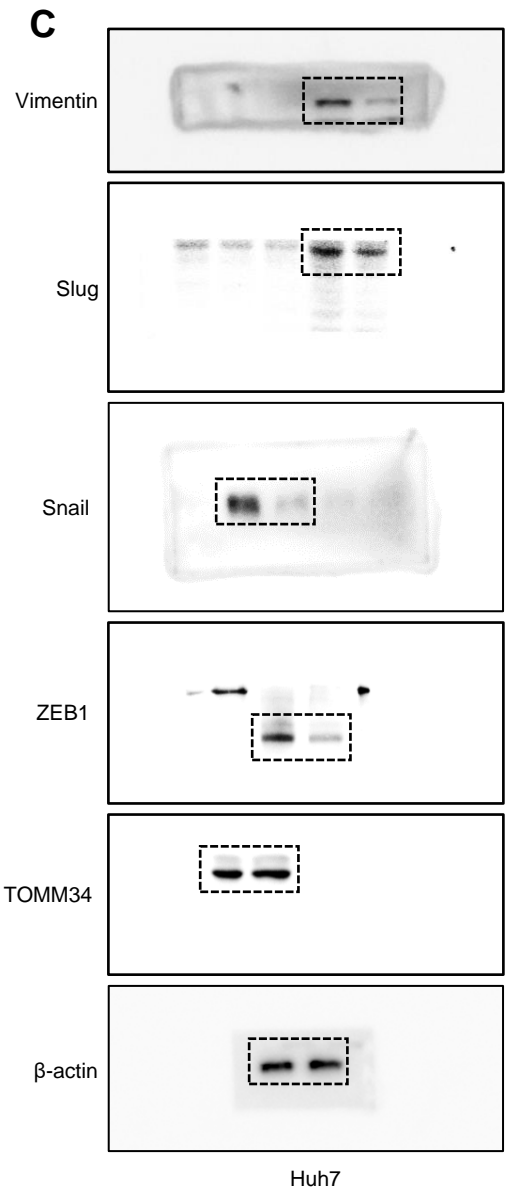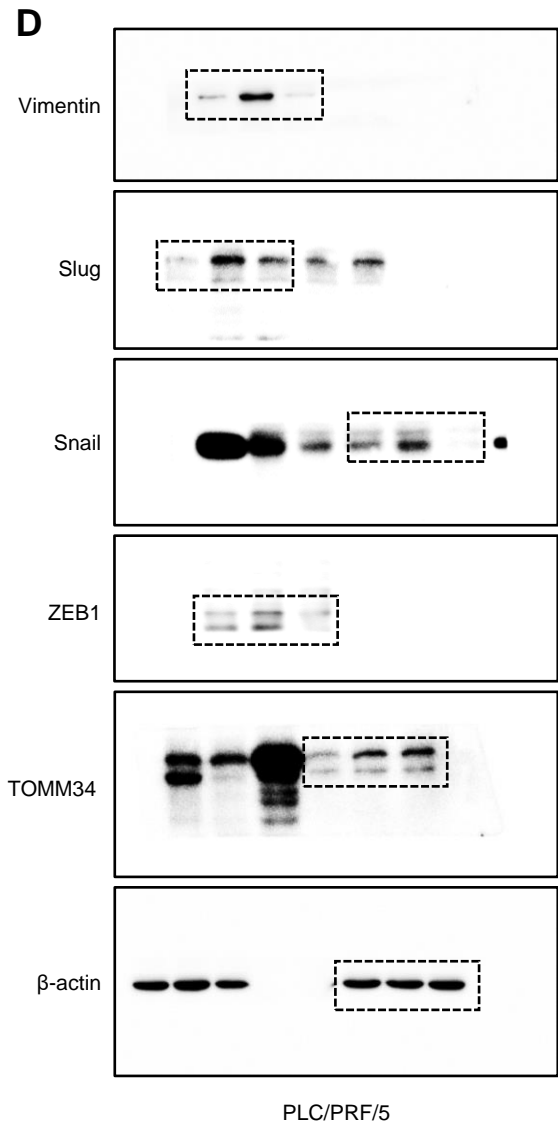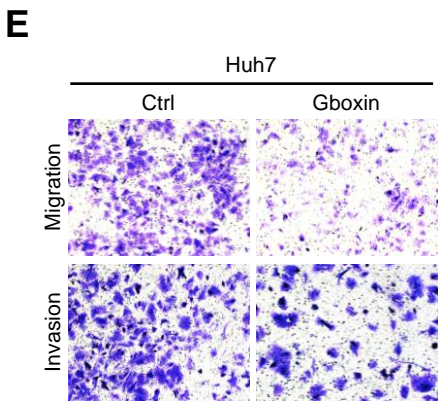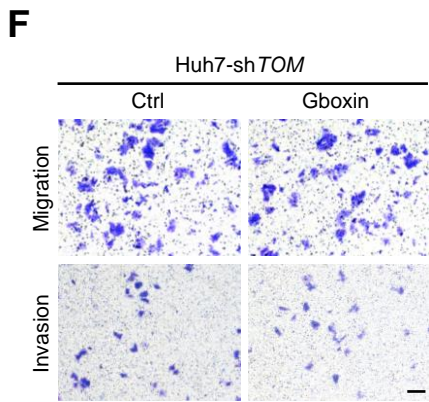

Fig. 6

H

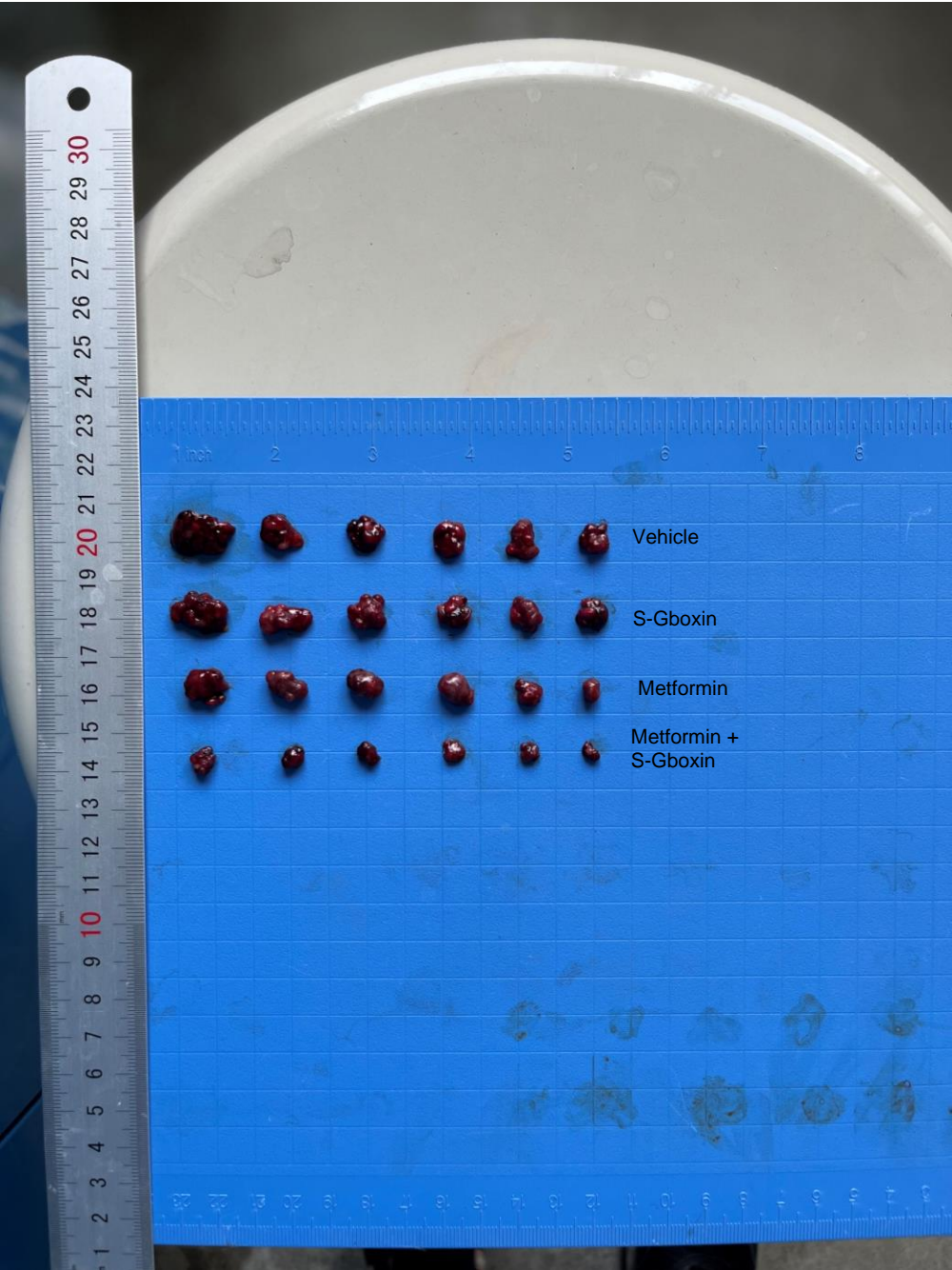

I

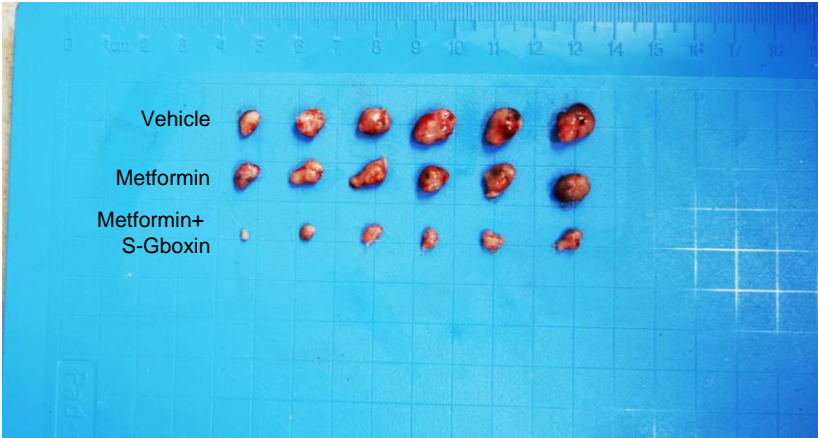

Supplement: Supplementary file 8 — Source Data for Figure 6 [file EMMM-14-e16082-s003.pdf]
