# Supplementary material for: Disrupting metformin adaptation of liver cancer cells by targeting the TOMM34/ATP5B axis
Source: EMBO Mol Med. 2022 Nov 2;14(12):e16082. doi: 10.15252/emmm.202216082 (PMC9728056; doi:10.15252/emmm.202216082)
Supplement: Supplementary file 9 — PDF+ [file EMMM-14-e16082-s009.pdf]

Article

SOURCE DATA | TRANSPARENT PROCESS | OPEN ACCESS

EMBO Molecular Medicine

Disrupting metformin adaptation of liver cancer cells by targeting the TOMM34/ATP5B axis

Ping Jin[1,†], Jingwen Jiang[1,†], Li Zhou[1], Zhao Huang[1], Siyuan Qin[1], Hai-Ning Chen[2], Liyuan Peng[1], Zhe Zhang[1], Bowen Li[1], Maochao Luo[1], Tingting Zhang[1], Hui Ming[3], Ning Ding[4], Lei Li[4], Na Xie[3], Wei Gao[5], Wei Zhang[6], Edouard C Nice[7], Yuquan Wei[8] & Canhua Huang[1,*]

Abstract

Metformin, a well-known antidiabetic drug, has been repurposed for cancer treatment; however, recently observed drug resistance and tumor metastasis have questioned its further application. Here, we found that long-term metformin exposure led to metabolic adaptation of hepatocellular carcinoma (HCC) cells, which was characterized by an obvious epithelial–mesenchymal transition (EMT) phenotype and compensatory elevation of oxidative phosphorylation (OXPHOS). TOMM34, a translocase of the outer mitochondrial membrane, was upregulated to promote tumor metastasis in response to metformin-induced metabolic stress. Mechanistically, TOMM34 interacted with ATP5B to preserve F_1F_O-ATPase activity, which conferred mitochondrial OXPHOS and ATP production. This metabolic preference for OXPHOS suggested a large requirement of energy supply by cancer cells to survive and spread in response to therapeutic stress. Notably, disturbing the interaction between TOMM34 and ATP5B using Gboxin, a specific OXPHOS inhibitor, increased sensitivity to metformin and suppressed tumor progression both *in vitro* and *in vivo*. Overall, this study demonstrates a molecular link of the TOMM34/ATP5B-ATP synthesis axis during metformin adaptation and provides promising therapeutic targets for metformin sensitization in cancer treatment.

Keywords hepatocellular carcinoma; metastasis; metformin adaptation; oxidative phosphorylation; TOMM34
Subject Categories Cancer; Metabolism
DOI 10.15252/emmm.202216082 | Received 27 March 2022 | Revised 23 September 2022 | Accepted 12 October 2022 | Published online 2 November 2022
EMBO Mol Med (2022) 14: e16082

Introduction

Metformin, a well-known dimethylbiguanide hypoglycaemic agent for treatment of diabetes, has been repurposed for effective treatment of several cancers including hepatocellular carcinoma (HCC), breast cancer, and lung cancer (Bo *et al*, 2012; Patterson *et al*, 2018; Shankaraiah *et al*, 2019). Nevertheless, drug resistance and tumor metastasis were frequently observed during long-term metformin treatment (Wheaton *et al*, 2014). For instance, the combinational treatment of metformin and chemoradiotherapy was associated with worse antitumor efficacy, accompanied with locoregional disease progression and distant metastases in locally advanced nonsmall-cell lung cancer (LA-NSCLC) in a randomized clinical trial (Tsakiridis *et al*, 2021). Moreover, metformin resistance caused continuous activation of epithelial–mesenchymal transition (EMT), which decreased the sensitivity of breast cancer cells to tamoxifen (Scherbakov *et al*, 2016). These observations limit the further application of metformin in cancer treatment. Recently, profiling of the bioactivity of metformin in primary breast cancer suggested the existence of metabolic adaptation to metformin (Lord *et al*, 2018). Typically, increased proliferation was observed in tumors with upregulated oxidative phosphorylation (OXPHOS) after metformin treatment. However, whether OXPHOS is involved in metformin adaptation and the possible underlying mechanism remains unclear. Thus, exploring the molecular basis of metformin adaptation in tumors will improve our understanding of metformin resistance and facilitate its future clinic application.

OXPHOS is an important metabolic pathway that couples the mitochondrial electron transport chain (ETC) for efficient ATP synthesis (Jourdain *et al*, 2021), in which ATPase plays an essential role to catalyze ATP production (Gore *et al*, 2022). Mounting evidence has demonstrated that tumor cells are prone to utilize OXPHOS for efficient ATP production, especially in the ever-

1 State Key Laboratory of Biotherapy and Cancer Center, Collaborative Innovation Center for Biotherapy, West China Hospital and West China School of Basic Medical Sciences and Forensic Medicine, Sichuan University, Chengdu, China
2 Colorectal Cancer Center, State Key Laboratory of Biotherapy and Cancer Center, West China Hospital, Sichuan University, Chengdu, China
3 West China School of Basic Medical Sciences & Forensic Medicine, Sichuan University, Chengdu, China
4 School of Basic Medical Sciences, Chengdu University of Traditional Chinese Medicine, Chengdu, China
5 Clinical Genetics Laboratory, Affiliated Hospital & Clinical Medical College of Chengdu University, Chengdu, China
6 Mental Health Center and Psychiatric Laboratory, The State Key Laboratory of Biotherapy, West China Hospital, Sichuan University, Chengdu, China
7 Department of Biochemistry and Molecular Biology, Monash University, Clayton, Vic, Australia
8 State Key Laboratory of Biotherapy and Cancer Center, Collaborative Innovation Center for Biotherapy, West China Hospital, Sichuan University, Chengdu, China
*Corresponding author. Tel: +86 13258370346; Fax: +86 28 85164060; E-mail: hcanhua@hotmail.com
[†]These authors contributed equally to this work

© 2022 The Authors. Published under the terms of the CC BY 4.0 license.

changing microenvironment during drug treatment and tumor metastasis (Bosc et al, 2017; Burke, 2017; Ashton et al, 2018; Zhang et al, 2022). In view of this, elucidating the molecular mechanisms involved in OXPHOS-mediated metabolic adaptation may provide new ideas for cancer treatment, for which a deep understanding of mitochondrial OXPHOS maintenance is the prerequisite (Eisner et al, 2018; Lisowski et al, 2018). Generally, mitochondrial quality control processes such as mitochondrial biogenesis and turnover are essential for sustaining OXPHOS under stress conditions (Suliman & Piantadosi, 2016; Wu et al, 2016; Jin et al, 2022). Indeed, several key regulators such as PTEN-induced putative kinase 1 (PINK1)/Parkin, Drp1 and OPA1 have been reported to play pivotal roles in maintaining mitochondrial quality, thus promoting tumor progression (Chen et al, 2020; Herkenne et al, 2020; Zheng et al, 2021). For instance, PGC-1α has been well-demonstrated to promote metastasis by enhancing mitochondrial biogenesis and OXPHOS (LeBleu et al, 2014). Notably, the TOM complex, the mitochondrial outer membrane proteins which are generally regarded as the entry site for nascent proteins to translocate into the mitochondria and contribute to preserving mitochondrial integrity, has been demonstrated to be involved in stress adaptation (Zhou et al, 2018; Pitt & Buchanan, 2021). For example, TOMM20 has been found to promote tumor proliferation and metastasis by enhancing mitochondrial ATP production in colorectal cancer (CRC) cells (Park et al, 2019). Nevertheless, further studies are urgently needed to elucidate the potential mechanisms underlying OXPHOS-mediated metabolic adaptation regulated by mitochondrial proteins in cancer cells.

Recent studies have suggested that overexpression of TOMM34, another outer membrane translocase (Nuttall et al, 1997), predicted poor prognosis in a number of cancers including liver cancer (Toraih et al, 2019), colorectal cancer (Zhang et al, 2014), and breast cancer (Aleskandarany et al, 2012). However, the mechanisms involved in TOMM34-regulated cancer progression and drug response remain largely unclear. In this study, we disclose the specific mechanism of TOMM34-mediated mitochondrial metabolic adaptation in HCC cells after long-term metformin exposure. Our experiments further demonstrate that Gboxin, a specific OXPHOS inhibitor, is indeed a potent mitochondrial energy antagonist that improves the inhibitory effect of metformin on HCC both *in vitro* and *in vivo*.

Results

Reactivated OXPHOS and ATP production are associated with metformin adaptation and consequent tumor metastasis of HCC

To confirm the existence of metabolic adaptation to metformin, we established patient-derived xenograft (PDX) models using NSG mice. About 1 month after subcutaneous transplantation, when the tumor volumes reached ~100 mm^3, mice received vehicle or metformin treatment for 17–22 days at escalating dose levels according to previously reported methods (Lord et al, 2018). The results showed that metformin slightly suppressed tumor growth (Fig 1A). However, enhanced lung and liver metastases were observed in the escalating metformin-treated group, indicating the occurrence of metformin adaptation (Fig 1B and C). We then set out to explore the metabolic alteration of HCC cells in response to long-term metformin treatment. Huh7 and PLC/PRF/5 HCC cells were gradually exposed to increasing concentrations of metformin over at least 12 days, according to a previous study (Andrzejewski et al, 2017). The residual population was capable of growth in medium containing 5 mM metformin (Fig 1D), and they were more resistant to metformin (Fig 1E). It is worthwhile noting that, although the proliferation rate of these residual HCC cells was decreased (Fig EV1A), they displayed an obvious EMT and polarized phenotype (Fig 1F and G) and obtained enhanced migratory and invasive capacity after metformin withdrawal (Fig 1H). We therefore defined this phenotype as metformin adaptation, and these residual cells were named as metformin-adaptive HCC cells.

Since upregulation of OXPHOS was observed to be linked with metabolic adaptation to long-term metformin treatment (Lord et al, 2018), we next focused on the alterations of OXPHOS and bioenergetic profile of metformin-adaptive HCC cells. Our results suggested that metformin-adaptive HCC cells exhibited a relatively higher respiration rate (Fig 1I) and cellular ATP levels (Fig 1J). Further results revealed a reduction in mitochondrial complex I activity in metformin-adaptive HCC cells (Fig EV1B), which is in line with the long-standing belief that metformin inhibits the activity of mitochondrial respiratory chain complex I (Pollak, 2012). Unexpectedly, metformin-adaptive HCC cells exhibited enhanced mitochondrial complex V activity (Fig 1K). Additionally, the activity of other

Figure 1. Elevated OXPHOS and ATP production are associated with metformin adaptation and consequent metastasis.

A NSG mice were used to establish the HCC PDX model with or without escalating dosage of metformin treatment for 17–22 days. Scale bars, 1 cm. ($n = 5$ mice for each group, Student's *t*-test).

B, C Representative images of lung metastasis and liver metastasis in HCC PDX models in NSG mice. Yellow arrowheads indicate liver metastases. Scale bars, (B) 1,000 μm; (C) (Left) 75 μm, (Right) 20 μm. ($n = 3$ mice for each group in B, Student's *t*-test).

D Schematic diagram describing the process of long-term metformin treatment for Huh7 and PLC/PRF/5 cells.

E The relative number of living cells was valuated after the parental or metformin-adaptive HCC cells being treated with indicated concentration of metformin for 24 h. ($n = 4$ biological replicates, Two-way ANOVA).

F Morphology of parental and metformin-adaptive HCC cells. Scale bars, 25 μm.

G Cell polarity in (F) was shown by Rose plots.

H Migration and invasion of parental and metformin-adaptive HCC cells were evaluated by Transwell assays (5×10^4 cells). Scale bars, 100 μm. ($n = 4$ biological replicates, Two-way ANOVA).

I The OCR of parental Huh7, adaptive Huh7, parental PLC/PRF/5 and adaptive PLC/PRF/5 cells was measured (Oligo, oligomycin; FCCP, Carbonyl cyanide 4-(trifluoromethoxy)phenylhydrazone; R/A, rotenone/antimycin). ($n = 3$ biological replicates).

J Cellular ATP levels of parental and adaptive HCC cells. ($n = 4$ biological replicates, Two-way ANOVA).

K The activity of the mitochondrial respiratory chain complex V in parental and adaptive HCC cells. ($n = 4$ technical replicates, Two-way ANOVA).

Data information: Data are presented as means ± SD.
Source data are available online for this figure.

 © 2022 The Authors

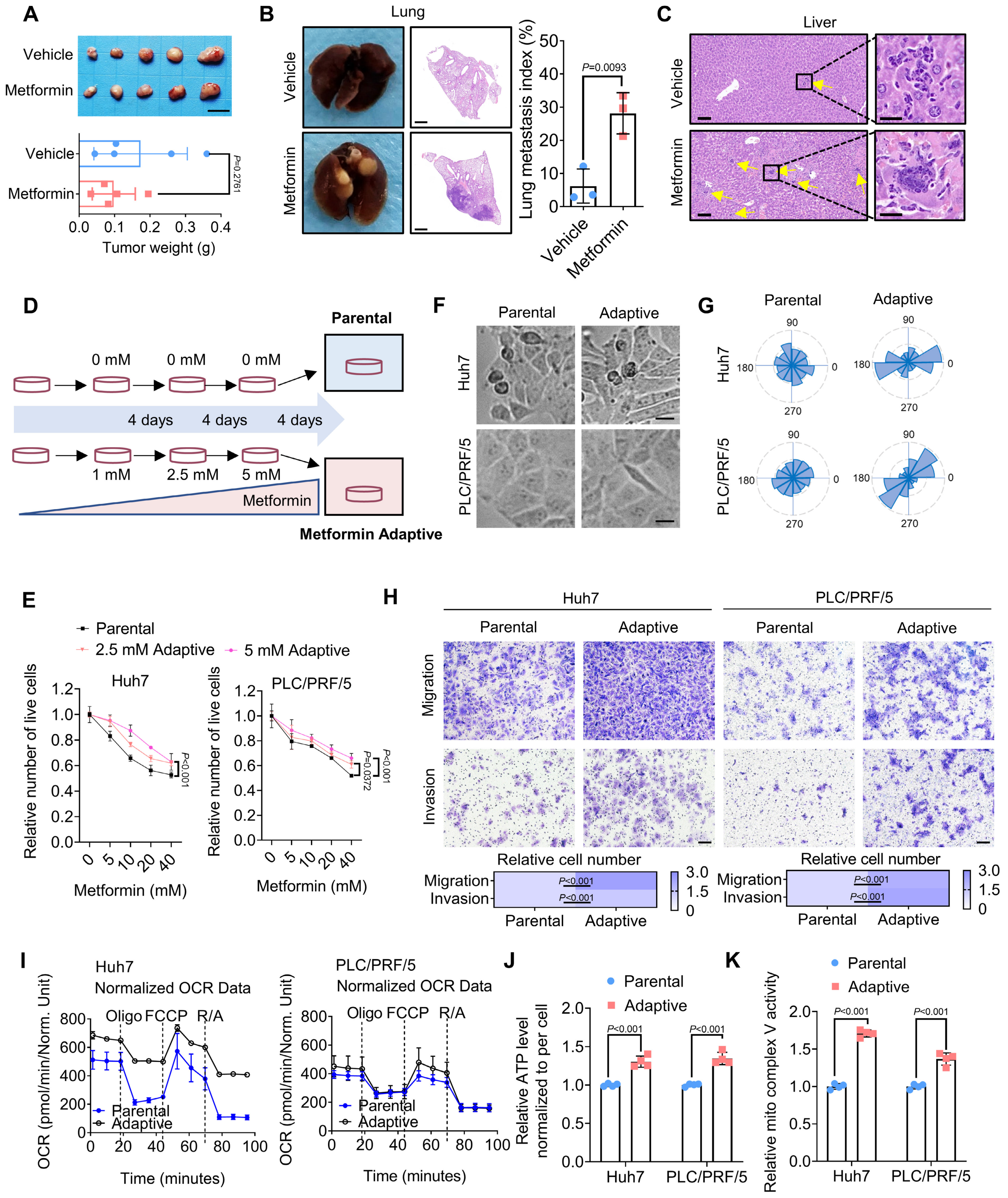
A
Vehicle
Metformin
Vehicle
Metformin
P=0.2761
0.0 0.1 0.2 0.3 0.4
Tumor weight (g)
B
Lung
Vehicle
Metformin
Lung metastasis index (%)
P=0.0093
Vehicle
Metformin
C
Liver
Vehicle
Metformin
D
Parental
0 mM 0 mM 0 mM
4 days 4 days 4 days
1 mM 2.5 mM 5 mM
Metformin
Metformin Adaptive
F
Parental Adaptive
Huh7
PLC/PRF/5
G
Parental Adaptive
Huh7
PLC/PRF/5
E
Parental
2.5 mM Adaptive
5 mM Adaptive
Huh7
PLC/PRF/5
Relative number of live cells
P<0.001
P<0.001
P=0.0372
Metformin (mM)
H
Huh7
PLC/PRF/5
Parental Adaptive Parental Adaptive
Migration
Invasion
Relative cell number
P<0.001
P<0.001
Parental Adaptive
I
Huh7
Normalized OCR Data
PLC/PRF/5
Normalized OCR Data
Oligo FCCP R/A
Parental
Adaptive
OCR (pmol/min/Norm. Unit)
Time (minutes)
J
Parental
Adaptive
Relative ATP level normalized to per cell
P<0.001
Huh7 PLC/PRF/5
K
Parental
Adaptive
Relative mito complex V activity
P<0.001
Huh7 PLC/PRF/5

Figure 1.

mitochondrial complexes (including II, III, and IV) in adaptive cells was preserved at a relatively high level compared with those in parental cells (Fig EV1C–E), which might contribute to the increased intracellular ATP level. Meanwhile, there were no obvious changes in cellular lactate levels between parental and metformin-adaptive HCC cells, indicating that glycolysis was not significantly influenced during metformin adaptation (Fig EV1F). Collectively, these results revealed that a hyperactive mitochondrial complex might work as a compensatory pathway to sustain ATP generation for tumor cells surviving through long-term metformin exposure and obtaining aggressive phenotypes.

TOMM34 promotes HCC growth and metastasis during metformin adaptation

To screen the key factors facilitating metformin adaptation, parental and adaptive HCC cells were subjected to label-free mass spectrometric analysis. Twenty-six overlapping and significantly changed proteins were identified by integrating mass spectrometry (MS) results and the MitoCarta 3.0 database (Fig 2A and B). Among these proteins, TOMM34, the top-ranking mitochondrial outer membrane protein, is of particular interest due to the critical role of the outer mitochondrial membrane in stress response (Fig 2B). We then verified increased mRNA and protein levels of TOMM34 in metformin-adaptive cells using qPCR and immunoblot analysis (Appendix Fig S1A and Fig 2C). Furthermore, TOMM34 was upregulated in metformin-treated PDX tissues (Fig EV2A). To verify the impact of TOMM34 on metabolic adaptation of HCC cells, we first measured the proliferation of HCC cells with TOMM34 overexpression or knockdown in response to metformin treatment. We found that TOMM34 manipulation had little effect on HCC cell proliferation (Fig 2D), while it could regulate the survival of HCC cells upon metformin treatment (Fig 2E and F). Furthermore, TOMM34 was upregulated in response to metabolic stress induced by 2-DG, glucose deprivation and H_2O_2 treatment (Appendix Fig S1B). Moreover, TOMM34 deficiency rendered cancer cells more vulnerable to such metabolic stress, while overexpression of TOMM34 assisted HCC cells to survive through these metabolic stresses (Appendix Fig S1C–H). Collectively, these data suggested that TOMM34 is critical for cancer cell survival under diverse metabolic stresses including metformin treatment.

As metabolic stress is a critical barrier during cancer metastasis (Chen *et al*, 2021), and metformin-adaptive cells exhibited a mesenchymal cell phenotype, we hypothesized that TOMM34-mediated metabolic adaptation might promote HCC metastasis. To this end, we generated an orthotopic mouse model of HCC to evaluate the role of TOMM34 in tumor metastasis (Fig EV2B). IHC assays suggested that the protein level of TOMM34 was higher in lung metastases than the primary tumor, indicating that TOMM34 upregulation may be essential for tumor metastasis. We therefore investigated the morphology of metformin-adaptive Huh7 cells before and after TOMM34 knockdown and found that knockdown of TOMM34 led to an epithelial phenotype (Fig 2G). Consistently, TOMM34 knockdown significantly attenuated the migration and invasion of metformin-adaptive Huh7 cells (Fig 2G and H). Moreover, we also generated TOMM34 knockdown adaptive PLC/PRF/5 cells and TOMM34-overexpressed adaptive Huh7 cells and obtained similar findings (Appendix Fig S2A–E). Furthermore, a tail vein injection BALB/c nude mouse model was established using metformin-adaptive Huh7 cells, and hematoxylin and eosin (HE) staining demonstrated that loss of TOMM34 significantly attenuated the formation of lung metastases (Fig 2I).

To further ascertain the function of TOMM34 in HCC metastasis, TOMM34 stably knocked down or overexpressed HCC cells were injected into the tail vein of BALB/c nude mice. TOMM34 deficiency was found to attenuate lung metastases (Fig 2J), while

Figure 2. TOMM34 promotes HCC growth and metastasis during metformin adaptation.

A The differentially expressed genes in adaptive versus parental HCC cells were determined by label-free fast quantitative analysis, and the mitochondrial-related candidates were further enriched using MitoCarta database 3.0. (https://www.broadinstitute.org/mitocarta/mitocarta30-inventory-mammalian-mitochondrial-proteins-and-pathways).

B Heatmap showing the abundance of proteins (LFQ intensity) identified in a label-free fast quantitative analysis. (|Fold change| > 1, Unique peptides > 2, Score > 20).

C Immunoblots analysis of TOMM34 expression in parental and metformin-adaptive HCC cells.

D Relative growth rates of HCC cells at indicated times were assessed by MTT assay in the absence of metformin (sh*TOM*, sh*TOMM34*, TOMM34 knockdown cells; OE-TOM, OE-TOMM34, TOMM34 overexpressed cells). (n = 4 biological replicates, Two-way ANOVA).

E The relative number of living cells showing the effects of TOMM34 on the viability of HCC cells treated with the indicated concentration of metformin for 24 h (sh*TOM*, sh*TOMM34*, TOMM34 knockdown cells; OE-TOM, OE-TOMM34, TOMM34 overexpressed cells). (n = 4 biological replicates, Two-way ANOVA).

F Trypan blue assay showing the survival of indicated cells treated with 20 mM metformin for 24 h (sh*TOM*, sh*TOMM34*, TOMM34 knockdown cells; OE-TOM, OE-TOMM34, TOMM34 overexpressed cells). (n = 4 biological replicates, Two-way ANOVA).

G The morphology (Left), migration (Middle) and invasion (Right) of metformin-adaptive Huh7 cells with (sh*TOM*) or without (sh*NC*) knockdown of TOMM34 are shown (5×10^4 cells for Transwell assay). Scale bars, (Left) 25 μm, (Middle) and (Right) 100 μm. (n = 3 biological replicates, Two-way ANOVA).

H Wound healing assay showing the migration of metformin-adaptive Huh7 cells with (sh*TOM*) or without (sh*NC*) TOMM34 knockdown. Scale bars, 200 μm. (n = 4 technical replicates, Student's t-test).

I A tail vein injection model was established using adaptive Huh7 cells to assess cell migratory ability. Scale bars, (Left) 1,000 μm, (Right) 25 μm. (n = 6 mice for each group, Student's t-test).

J, K The effects of TOMM34 on HCC lung metastasis in the tail vein injection mouse model (sh*TOM*, sh*TOMM34*, TOMM34 knockdown cells; OE-TOM, OE-TOMM34, TOMM34 overexpressed cells). Scale bars, (Left) 1,000 μm, (Right) 25 μm. (n = 5 mice for each group, Student's t-test).

L Western blot analysis showing the effects of TOMM34 on the expression of EMT markers in HCC cells (sh*TOM*, sh*TOMM34*, TOMM34 knockdown cells; OE-TOM, OE-TOMM34, TOMM34 overexpressed cells).

M The protein expression of TOMM34 in clinical HCC samples with or without metastasis. Scale bars, (Left) 100 μm, (Right) 25 μm. (n = 33 in metastasis present group, n = 53 in metastasis absent group, Student's t-test).

Data information: Data are presented as means ± SD.

© 2022 The Authors

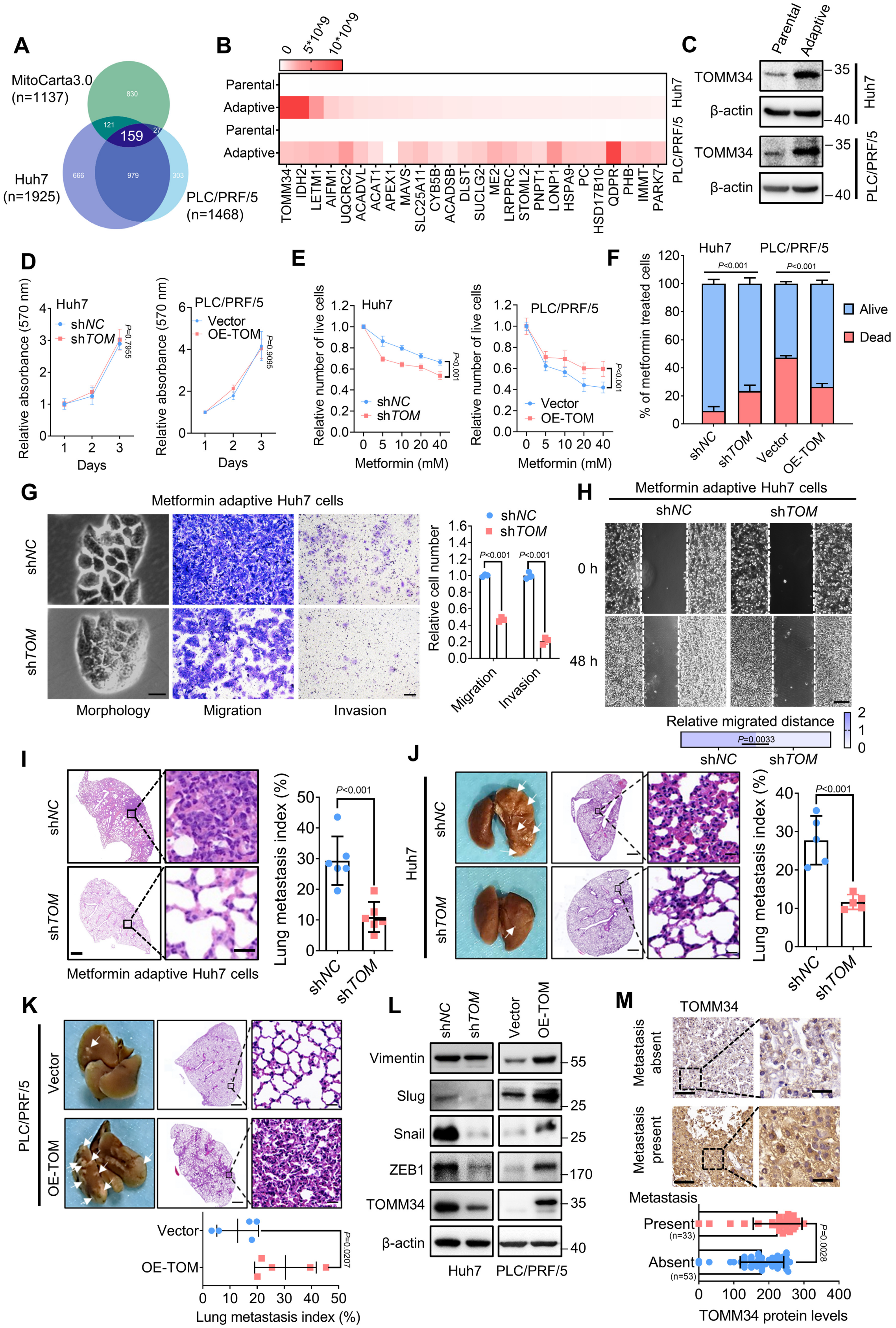
A
MitoCarta3.0
(n=1137)
Huh7
(n=1925)
PLC/PRF/5
(n=1468)
159
B
Parental
Adaptive
TOMM34
IDH2
LETM1
AIFM1
UQCRC2
ACADVL
ACAT1
APEX1
MAVS
SLC25A11
CYB5B
ACADSB
DLST
SUCLG2
ME2
LRPPRC
STOML2
PNPT1
LONP1
HSPA9
PC
HSD17B10
QDPR
PHB
IMMT
PARK7
C
Parental
Adaptive
TOMM34
β-actin
Huh7
PLC/PRF/5
D
Relative absorbance (570 nm)
Days
shNC
shTOM
Vector
OE-TOM
E
Relative number of live cells
Metformin (mM)
F
% of metformin treated cells
Alive
Dead
G
Metformin adaptive Huh7 cells
Morphology
Migration
Invasion
Relative cell number
H
Metformin adaptive Huh7 cells
0 h
48 h
Relative migrated distance
I
Metformin adaptive Huh7 cells
Lung metastasis index (%)
J
K
L
Vimentin
Slug
Snail
ZEB1
TOMM34
β-actin
M
TOMM34
Metastasis absent
Metastasis present
Metastasis
Present (n=33)
Absent (n=53)
TOMM34 protein levels

Figure 2.

overexpression of TOMM34 exhibited the opposite effects (Fig 2K). *In vitro* experiments also demonstrated the similar role of TOMM34 on cell mobility and invasive capacity of HCC cells (Figs 2L and EV2C–F). Loss of TOMM34 also impeded the mesenchymal morphology of HCC cells, while overexpression of TOMM34 induced the mesenchymal features (Fig EV2G). In addition, we evaluated the expression of TOMM34 in clinical HCC samples by IHC analysis and found that TOMM34 was increased in samples with metastatic nodules (Fig 2M), which is consistent with results from GEO datasets (GSE3500; Fig EV2H). Moreover, the HCC patients with higher TOMM34 levels exhibited shorter overall survival (OS; Fig EV2I). Together, these data revealed that TOMM34 induces EMT programming during metformin adaptation and highlighted the pivotal role of TOMM34 in HCC progression and metastasis.

TOMM34 preserves mitochondrial OXPHOS and ATP production in HCC cells

To investigate the mechanisms underlying TOMM34-mediated HCC metastasis, total RNAs from Huh7 cells with or without TOMM34 knockdown were subjected to RNA-sequencing. Kyoto Encyclopedia of Genes and Genomes (KEGG) pathway analysis and Gene Set Enrichment Analysis (GSEA) revealed that OXPHOS was significantly altered upon TOMM34 deficiency (Fig 3A and B). Transmission electron microscopy (TEM) revealed that cells after TOMM34 knockdown exhibited a disorganized internal structure evidenced by vacuolating or swelling mitochondria and blurred or even disappeared cristae (Fig 3C). To further demonstrate the association between TOMM34 expression and mitochondrial functions, we measured mitochondrial DNA content, cellular ATP levels, and oxygen consumption rate (OCR) upon manipulation of TOMM34 expression. Consistently, TOMM34 knockdown decreased mtDNA content and intracellular ATP levels, while overexpression of TOMM34 exhibited the opposite effect (Fig 3D and E). Moreover, TOMM34 deficiency impaired OCR of HCC cells with inferior maximal and reserved respiration (Fig 3F). Besides, loss of TOMM34 decreased the expression of major mitochondrial OXPHOS complex subunits such as ATP5A1, UQCRC2, MTCO1, SDHB, and NDUFB3, while TOMM34 overexpression induced the upregulation of these proteins (Fig 3G). Consistently, the activities of complex I and complex V were significantly decreased in TOMM34 deficient Huh7 cells but increased in TOMM34 overexpressed PLC/PRF/5 cells (Fig 3H). However, using Mito-Tracker green, a mitochondrial-specific staining dye, we found that TOMM34 had little impact on mitochondrial mass (Appendix Fig S3A and B). Moreover, TOMM34 knockdown also diminished cellular ATP levels and decreased mitochondrial complex activities in metformin-adaptive HCC cells (Fig 3I and J). Altogether, these results demonstrated the critical role of TOMM34 in maintaining mitochondrial integrity, thereby sustaining OXPHOS and ATP production in HCC cells during metformin adaptation.

TOMM34 interacts and positively correlates with ATP5B

We then investigated the molecular basis involved in TOMM34-regulated maintenance of ATP production in HCC cells during metformin adaptation. Immunoprecipitation (IP) followed by MS assay was employed to identify potential TOMM34 interacting proteins, and ATP5B, a pivotal subunit of ATPase was identified (Fig 4A). Co-IP experiments were then performed to validate the interaction between Myc-tagged TOMM34 and Flag-tagged ATP5B in HEK293T cells (Fig 4B). In addition, the endogenous interaction between TOMM34 and ATP5B was confirmed in Huh7 cells by Co-IP assay and proximity ligation assay (PLA; Fig 4C and D). Considering the role of the TOM complex in transporting precursor protein into mitochondria and maintaining mitochondrial stability (Liu *et al*, 2018; Gomkale *et al*, 2020), we wondered whether TOMM34 affects the expression of ATP5B. As shown in Fig 4E, TOMM34 had no obvious impact on the mRNA levels of ATP5B. Notably, Western blot analysis suggested that TOMM34 knockdown reduced protein levels of ATP5B, while overexpression of TOMM34 increased ATP5B protein expression. Given that TOMM34 can upregulate the protein level of ATP5B without affecting its mRNA, we postulated that the ATP5B protein stability might be modulated by TOMM34.

Figure 3. TOMM34 preserves mitochondrial OXPHOS and ATP production in HCC cells.

A, B KEGG and GSEA (Gene Set Enrichment Analysis) analysis of the RNA-seq data identified the oxidative phosphorylation pathway as the one of most enriched signaling regulated by TOMM34. ($P < 0.020$ in (A), $P = 0.0075$ in (B)).

C Transmission electron microscopy (TEM) of Huh7 sh*NC* and sh*TOM* (sh*TOMM34*) cells. Yellow arrowheads indicate mitochondria, bule arrowheads indicate nucleus, red arrowheads indicate endoplasmic reticulum. Scale bars, (Left) 20 μm, (Middle) 4 μm, (Right) 1 μm. ($n = 5$ biological replicates, One-way ANOVA).

D mtDNA content in HCC cells (sh*TOM*, sh*TOMM34*, TOMM34 knockdown cells; OE-TOM, OE-TOMM34, TOMM34 overexpressed cells). ($n = 4$ biological replicates, Student's *t*-test).

E Intracellular ATP levels in HCC cells (sh*TOM*, sh*TOMM34*, TOMM34 knockdown cells; OE-TOM, OE-TOMM34, TOMM34 overexpressed cells). ($n = 6$ biological replicates in Huh7 cells, $n = 3$ biological replicates in MHCC97H cells, $n = 5$ biological replicates in PLC/PRF/5 cells, Student's *t*-test).

F Oxygen consumption rate (OCR) in Huh7 and HMCC97H cells with (sh*TOM*) or without (sh*NC*) TOMM34 knockdown was measured using a Seahorse XF24 Extracellular Analyzer (Oligo, oligomycin; FCCP, Carbonyl cyanide 4-(trifluoromethoxy)phenylhydrazone; R/A, rotenone/antimycin). ($n = 3$ biological replicates).

G Western blot analysis showing the protein expression of the main mitochondrial respiratory chain complex subunit (sh*TOM*, sh*TOMM34*, TOMM34 knockdown cells; OE-TOM, OE-TOMM34, TOMM34 overexpressed cells).

H Mitochondrial respiratory chain complex activity in HCC cells with or without TOMM34 knockdown/overexpression (sh*TOM*, sh*TOMM34*, TOMM34 knockdown cells; OE-TOM, OE-TOMM34, TOMM34 overexpressed cells). ($n = 3$ technical replicates, Two-way ANOVA).

I Intracellular ATP levels in adaptive Huh7 cells normalized to cells with (sh*TOM*) or without (sh*NC*) TOMM34 knockdown. ($n = 4$ biological replicates, Two-way ANOVA).

J Detection of mitochondrial respiratory chain complex activity in metformin-adaptive HCC cells with (sh*TOM*) or without (sh*NC*) TOMM34 knockdown. ($n = 4$ technical replicates, Two-way ANOVA).

Data information: Data are presented as means ± SD.
Source data are available online for this figure.

© 2022 The Authors

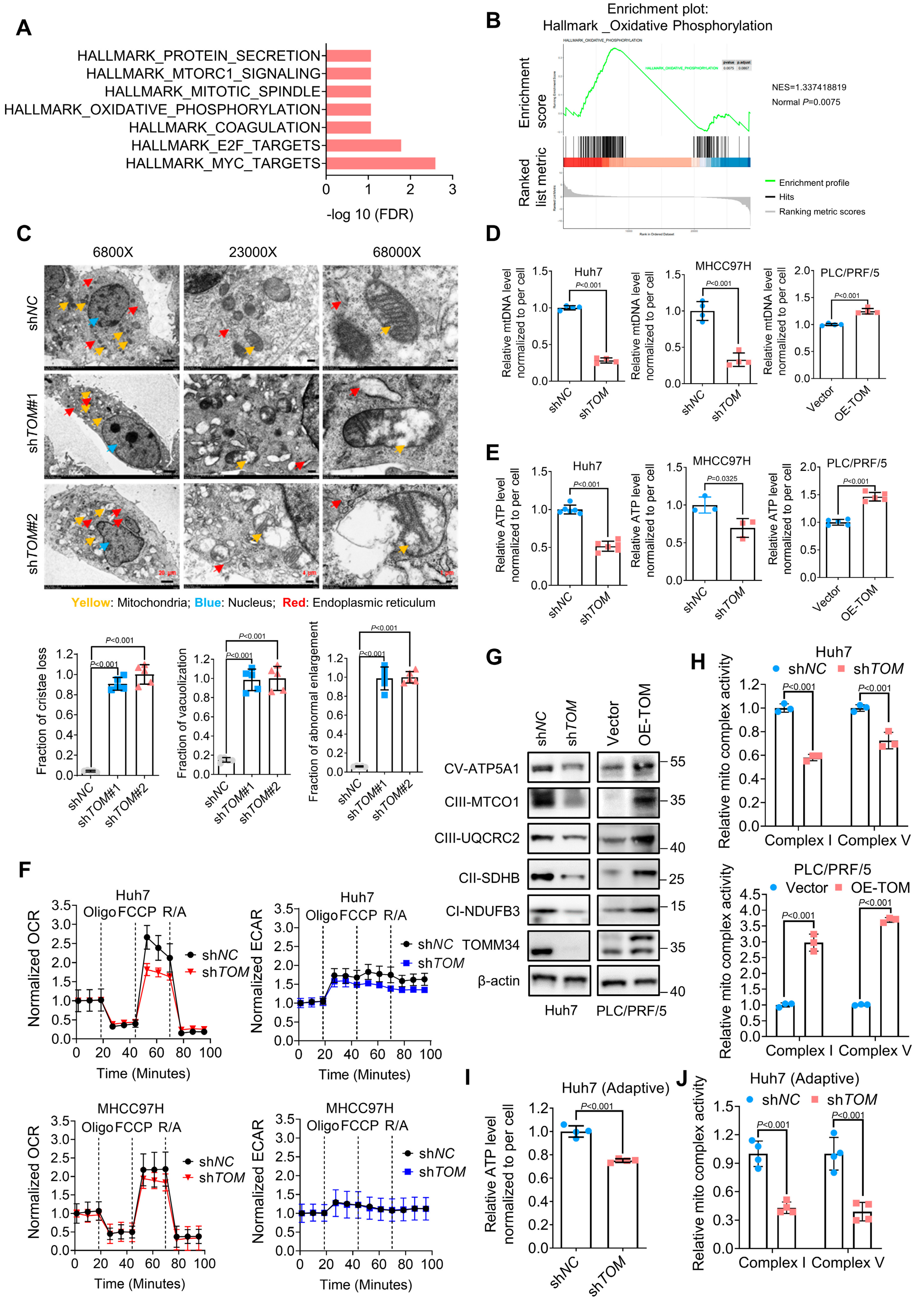

A
HALLMARK_PROTEIN_SECRETION
HALLMARK_MTORC1_SIGNALING
HALLMARK_MITOTIC_SPINDLE
HALLMARK_OXIDATIVE_PHOSPHORYLATION
HALLMARK_COAGULATION
HALLMARK_E2F_TARGETS
HALLMARK_MYC_TARGETS
-log 10 (FDR)
B
Enrichment plot:
Hallmark _Oxidative Phosphorylation
Enrichment score
Ranked list metric
NES=1.337418819
Normal P=0.0075
Enrichment profile
Hits
Ranking metric scores
C
6800X
23000X
68000X
shNC
shTOM#1
shTOM#2
Yellow: Mitochondria; Blue: Nucleus; Red: Endoplasmic reticulum
Fraction of cristae loss
Fraction of vacuolization
Fraction of abnormal enlargement
P<0.001
D
Huh7
MHCC97H
PLC/PRF/5
Relative mtDNA level normalized to per cell
shNC
shTOM
Vector
OE-TOM
E
Relative ATP level normalized to per cell
P=0.0325
F
Huh7
MHCC97H
Oligo FCCP R/A
Normalized OCR
Normalized ECAR
Time (Minutes)
G
CV-ATP5A1
CIII-MTCO1
CIII-UQCRC2
CII-SDHB
CI-NDUFB3
TOMM34
β-actin
55
35
40
25
15
35
40
Huh7
PLC/PRF/5
H
Relative mito complex activity
Complex I
Complex V
I
Huh7 (Adaptive)
J
Huh7 (Adaptive)

Figure 3.

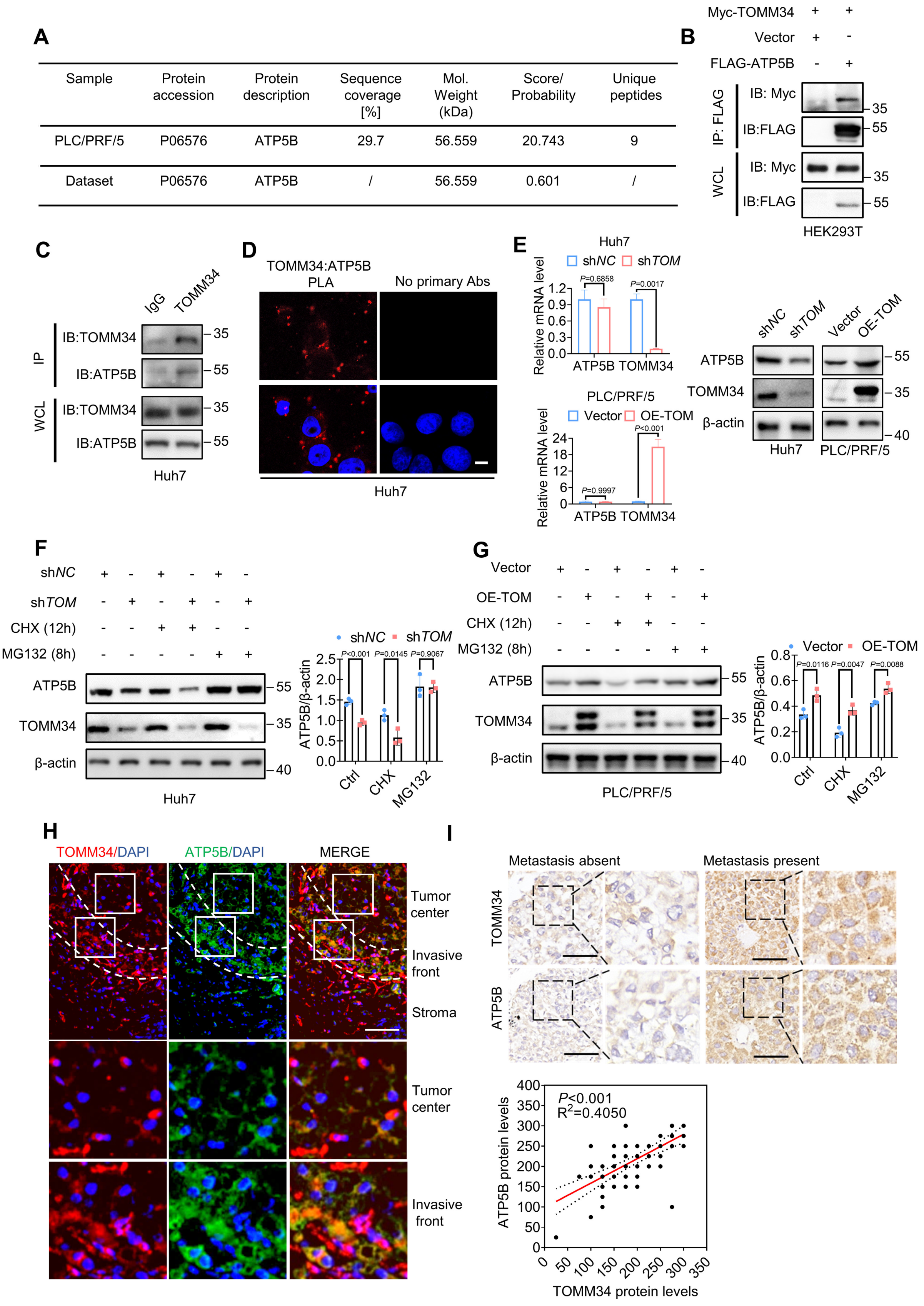

Figure 4.

© 2022 The Authors

Figure 4. TOMM34 interacts and positively correlates with ATP5B.

A Mass spectrometry and *PrePPI* dataset (https://bhapp.c2b2.columbia.edu/PrePPI/) identify the bait protein TOMM34 and its binding partner ATP5B.
B Co-IP assays indicate the interaction between exogenous TOMM34 and ATP5B in HEK293T cells.
C Co-IP assays indicate the interaction between endogenous TOMM34 and ATP5B in Huh7 HCC cells.
D The *in situ* interaction between TOMM34 and ATP5B was determined by proximity ligation assays (PLA). Scale bars, 10 μm.
E The mRNA and protein expression of ATP5B in indicated cells were determined by qPCR ($n = 3$ technical replicates, Two-way ANOVA) or immunoblotting (sh*TOM*, sh*TOMM34*, TOMM34 knockdown cells; OE-TOM, OE-TOMM34, TOMM34-overexpressed cells).
F, G Western blot showing protein level of ATP5B in Huh7 (F) or PLC/PRF/5 (G) cells treated with or without 10 mg/ml CHX for 12 h or 25 μM MG132 for 8 h. Relative gray value of ATP5B was calculated using Image J (sh*TOM*, sh*TOMM34*, TOMM34 knockdown cells; OE-TOM, OE-TOMM34, TOMM34 overexpressed cells; CHX, cyclohexane). ($n = 3$ technical replicates, Student's *t*-test).
H Tissue immunofluorescence assay showing the co-localization of TOMM34 and ATP5B at the invasive front of clinical HCC tissues. Scale bars, 20 μm.
I A positive correlation between TOMM34 and ATP5B levels in human HCC samples was shown by representative immunohistochemical images (Clinicopathologic characteristics are list in Appendix Table S1). Scale bars, 50 μm. ($n = 72$).

Data information: Data are presented as means ± SD.
Source data are available online for this figure.

To verify this, we detected the ATP5B protein level in cells with or without the treatment with cyclohexane (CHX) or MG132. As shown in Fig 4F and G, knockdown of TOMM34 accelerated the turnover of ATP5B protein, while overexpression of TOMM34 prolonged the half-life of ATP5B protein, indicating that TOMM34 may regulate the stability of the ATP5B protein. To further reveal the clinical relevance between TOMM34 and ATP5B, we performed tissue immunofluorescence assays. The results indicated that TOMM34 and ATP5B colocalized in the invasive front area (Fig 4H). Furthermore, we analyzed TOMM34 and ATP5B protein levels in our HCC TMA samples. Consistently, the ATP5B level was positively correlated with the TOMM34 expression level, both being highly expressed in metastatic HCC tissues (Fig 4I). Therefore, our results indicated that TOMM34 interacts with and stabilizes ATP5B.

We next mapped the region of TOMM34 responsible for binding with ATP5B. Six Myc-tagged TOMM34 truncated mutants (TOMM34-FL, Truncation 1, Truncation 2, Truncation 3, Truncation 4, Truncation 5, and Truncation 6) were generated (Fig EV3A), according to previous studies (Trcka *et al*, 2014, 2019). Co-IP and immunoblotting assays showed that TPR4, a 34-amino acid region (193–226), was essential for the interaction of TOMM34 with ATP5B (Fig EV3B). To prove the significance of the TOMM34/ATP5B interaction, we utilized the TPR4 region-truncated protein (Myc-TOM-TPR4-del) to perform migration and invasion rescue experiments. As shown in Fig EV3C–E, overexpression of full-length Myc-tagged TOMM34 (Myc-TOM-FL) restored the migration and invasion of endogenous TOMM34 knockdown HCC cells, while the Myc-TOM-TPR4-del failed to restore the metastatic potential of cells, indicating that TPR4 region-mediated TOMM34/ATP5B interaction is responsible for enhanced migratory and invasive capacities of metformin-adaptive HCC cells.

ATP5B is essential for TOMM34-mediated metformin adaptation and HCC metastasis

Next, we employed multiple assays to ascertain the role of ATP5B in TOMM34-regulated metformin adaptation and HCC metastasis. As shown in Fig 5A–C, silencing ATP5B impeded TOMM34-promoted ATP synthesis and the EMT process in metformin-adaptive HCC cells. Additionally, transwell and wound healing assays also demonstrated that ATP5B silencing significantly diminished the migratory and invasive capacities of adaptive cells (Fig 5D and E). Moreover, we overexpressed exogenous ATP5B (FLAG-ATP5B) in TOMM34 knockdown HCC cells, and found the cellular ATP level, expression of mesenchymal markers and their migratory and invasive abilities were significantly restored (Fig 5F–H). Therefore, these data implied that ATP5B participates in TOMM34-mediated metformin adaptation and HCC metastasis. As expected, ATP5B silencing also impaired the cellular ATP levels, mesenchymal markers and the migration and invasion of parental HCC cells (Fig EV4A–G). Given the vital role of ATP5B in mediating metformin adaptation and tumor metastasis, we next investigated the pathological significance of ATP5B in liver cancer. Bioinformatics analysis of the Iizuka Liver 2 dataset showed that high ATP5B levels were associated with higher risk of HCC recurrence (Appendix Fig S4A). Furthermore, Kaplan–Meier analyses from the TCGA dataset indicated that high ATP5B levels correlated with shorter overall survival time for HCC patients (Appendix Fig S4B). Moreover, TOMM34

Figure 5. ATP5B is essential for TOMM34-mediated metformin adaptation and HCC metastasis.

A ATP levels in adaptive HCC cells were measured with or without silencing of ATP5B by siRNAs. ($n = 4$ biological replicates, One-way ANOVA).
B, C Western blot showing the expression of indicated proteins in adaptive HCC cells with or without ATP5B silencing.
D, E Transwell assays showing the migration and invasion of adaptive HCC cells with or without the silence of ATP5B (5×10^4 adaptive Huh7 cells, 1×10^5 adaptive PLC/PRF/5 cells), Scale bars, 100 μm. ($n = 3$ biological replicates, Two-way ANOVA).
F ATP level of indicated cells was measured by ATP detection kit. ($n = 4$ biological replicates, One-way ANOVA).
G Western blot showing the expression of EMT markers in cells with (sh*TOM*) or without (sh*NC*) knockdown of TOMM34 or overexpression of ATP5B.
H Transwell assays showing the migration and invasion of indicated cells (5×10^4 cells, sh*TOM*, sh*TOMM34*, TOMM34 knockdown cells). Scale bars, 100 μm. ($n = 4$ biological replicates, two-way ANOVA).

Data information: Data are presented as means ± SD.
Source data are available online for this figure.

© 2022 The Authors

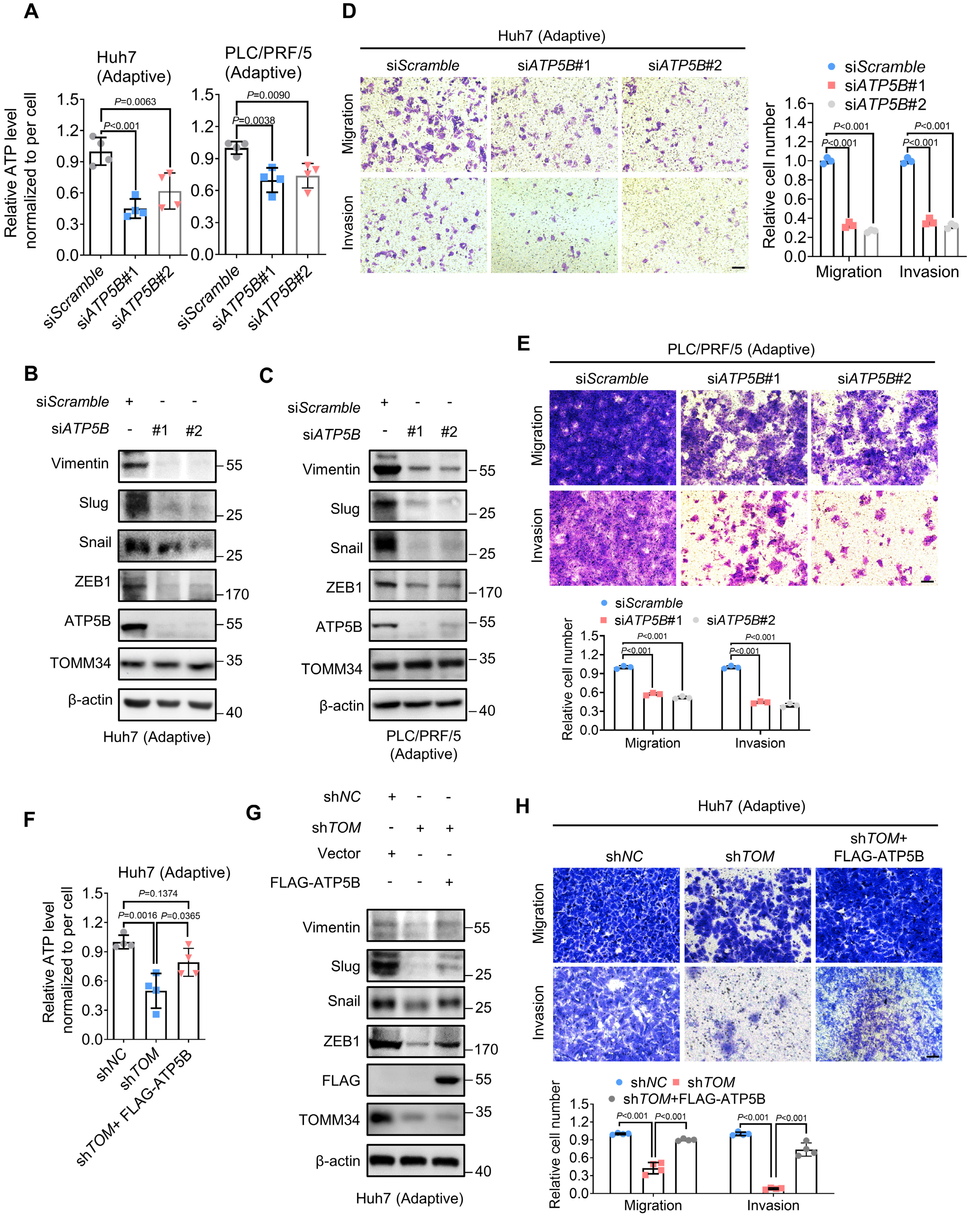

Figure 5.

© 2022 The Authors

upregulation in combination with high ATP5B expression exhibited the worst survival rate for HCC patients (Appendix Fig S4C). Taken together, our results suggested that ATP5B plays a critical role in TOMM34-mediated metformin adaptation, and high levels of ATP5B predict poor outcome for HCC patients.

Gboxin abrogates metabolic adaptation to metformin *in vitro* and *in vivo*

Gboxin, a OXPHOS inhibitor showing excellent antitumor activity in low-passage primary glioblastoma (GBM) cells, has been reported to interact with ATP5B (Shi *et al*, 2019). Therefore, we wondered whether Gboxin influences the interaction between TOMM34 and ATP5B thereby diminishing the metastatic potential of HCC cells. We first evaluated the cytostatic potential of Gboxin on HCC cells (Fig EV5A), and accordingly selected concentrations (200 and 400 nM) with little growth inhibitory effect for follow-up experiments. Co-IP and subsequent immunoblotting assays revealed that Gboxin abrogated the interaction between ATP5B and TOMM34 in a dose-dependent manner (Figs 6A and B, and EV5B). Effects of Gboxin on cell OCR and ECAR were then measured. As shown in Fig EV5C and D, Gboxin decreased OCR but had no obvious effect on ECAR. We next evaluated the effects of Gboxin on TOMM34/ATP5B-mediated tumor metastasis. At a relatively low concentration that had little growth inhibitory effect on HCC cells, Gboxin suppressed the expression of EMT markers while having no obvious effect on TOMM34 protein levels (Fig 6C). Moreover, Gboxin could also reduce the expression of EMT markers induced by TOMM34 overexpression (Fig 6D). We also found that Gboxin could inhibit the migration and invasion of HCC cells while having no obvious impact on the metastasis of TOMM34-deficient Huh7 cells (Fig 6E and F, and Appendix Fig S5A–C). Moreover, treatment of Gboxin could attenuate ATP production and subdue TOMM34 overexpression-mediated ATP increase (Fig 6G). Collectively, these results showed that Gboxin antagonizes the association between TOMM34 and ATP5B to inhibit HCC metastasis.

We further evaluated the feasibility of abrogating metabolic adaptation by combinational use of Gboxin and metformin for retarding HCC progression. Transwell assays revealed that combinational of Gboxin and metformin could dramatically inhibit the metastatic potential of both parental and metformin-adaptive HCC cells (Fig EV5E). Furthermore, tail vein injection model and a subcutaneously injection xenograft model also confirmed the inhibitory effects of S-Gboxin (a functional analog that is suitable for *in vivo* studies (Shi *et al*, 2019)) on metformin adaptation *in vivo* (Figs 6H and EV5F). However, the S-Gboxin alone exhibited no significant antitumor effect over the control group, in contrast to the robust anticancer efficacy of combinational group. In addition, a PDX model was established to confirm the synergistic antitumor effect of S-Gboxin and metformin. Xenografts with combinatorial treatment exhibited remarkably reduced tumor, evidenced by the reduction of tumor size, weight and circulating tumor cell (CTC) in PDX models (Fig 6I and J). Moreover, we also determined the proliferation, migration, and invasion of noncancerous liver cells LO2 upon combinational treatment with metformin and Gboxin. As shown in Appendix Fig S5D–F, combinational treatment with metformin and Gboxin had no obvious effect on the proliferation, migration, and invasion of LO2 cells, in contrast to its inhibitory effects on HCC cells. These findings suggest limited cytotoxicity of metformin plus Gboxin to noncancerous liver cells. In summary, our data revealed a potential combination therapy strategy of S-Gboxin and metformin in retarding HCC progression.

Discussion

The main obstacles for the treatment of liver cancer include drug tolerance and tumor metastasis, during which metabolic stress generally occurs (Feng *et al*, 2011; Yu *et al*, 2018). Cancer cells usually undergo metabolic adaptations to provide energetic flexibility to survive stress conditions and obtain an aggressive phenotype (Chen *et al*, 2016; Schito & Rey, 2018; Cai *et al*, 2020; Kowalik *et al*, 2020; Liu *et al*, 2021). In the present study, we identified that elevation of TOMM34 is pivotal for facilitating HCC cells to overcome energetic stress induced by metformin. Further study found that TOMM34 interacted with ATP5B to maintain OXPHOS and ATP production, which replenished energy to survive metabolic stress and retain the migratory and invasive characteristics in HCC cells. Disrupting TOMM34/ATP5B axis by Gboxin impaired the compensatory adaptive process and tumor metastasis in both HCC cell lines and PDX models. Our work outlined the molecular basis underlying the adaptation of tumor cells to metformin-induced energetic crisis and linked the mitochondrial response to metabolic reprogramming upon metformin exposure. Intervening such TOMM34-mediated metabolic adaptation might hold the potential to suppress tumor metastasis and achieve better clinical outcomes for cancer patients.

Figure 6. Gboxin abrogates metabolic adaptation to metformin *in vitro* and *in vivo*.

A, B Co-IP assay was performed to detect the interaction between TOMM34 and ATP5B treated with or without Gboxin for 24 h in parental or adaptive PLC/PRF/5 cells.
C, D Immunoblotting of EMT markers of HCC cells treated with or without 200 nM Gboxin for 24 h (sh*TOM*, sh*TOMM34*, TOMM34 knockdown cells; OE-TOM, OE-TOMM34, TOMM34 overexpressed cells).
E, F Transwell assay showing the migration and invasion of HCC cells treated with or without 200 nM Gboxin for 36 h (5×10^4 cells; sh*TOM*, sh*TOMM34*, TOMM34 knockdown cells). Scale bars, 100 μm. ($n = 3$ biological replicates, two-way ANOVA).
G Cellular ATP levels of HCC cells treated with or without 200 nM Gboxin for 24 h (OE-TOM, OE-TOMM34, TOMM34 overexpressed cells). ($n = 6$ biological replicates in Huh7, $n = 4$ biological replicates in PLC/PRF/5, one-way ANOVA).
H Adaptive Huh7 cells were subcutaneously injected into mice. The mice then received Gboxin, metformin or combinational treatment. The image and weight of tumor xenografts were shown. Scale bars, 1 cm. ($n = 6$ mice in each group, one-way ANOVA).
I Images and weights of isolated tumors from HCC PDX models. Scale bars, 1 cm. ($n = 6$ mice in each group, one-way ANOVA).
J Circulating tumor cell (CTC) was detected in HCC PDX models. ($n = 3$ biological replicates, one-way ANOVA).

Data information: Data are presented as means ± SD.
Source data are available online for this figure.

© 2022 The Authors

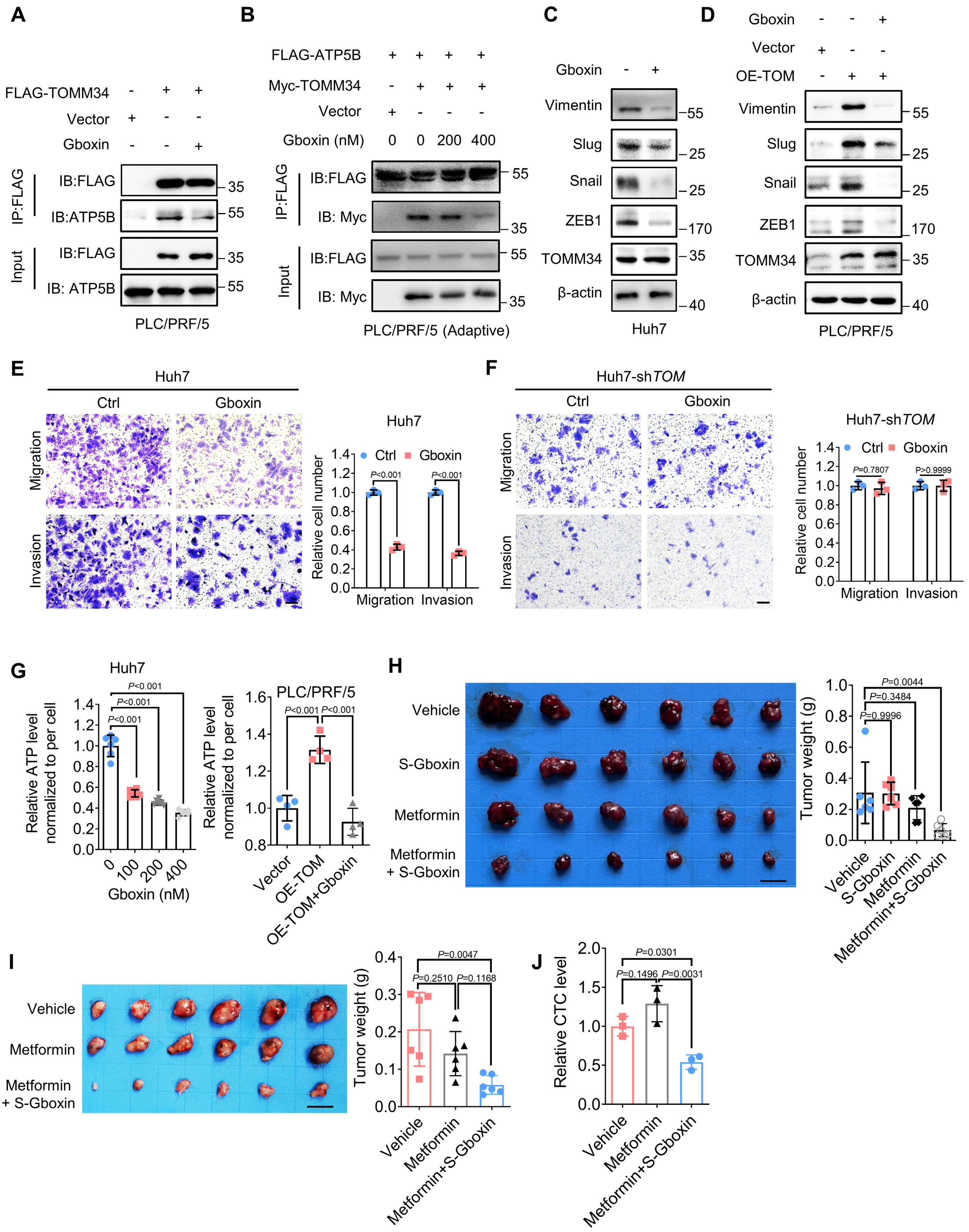

Figure 6.

© 2022 The Authors

Multiple studies including epidemiologic and clinical investigations have confirmed the antitumor effect of metformin on a variety of tumor types (Coyle *et al*, 2016; De & Kuppusamy, 2020). However, controversies remain as drug resistance and tumor metastasis occurred mutually in many cancers (Scherbakov *et al*, 2016; Lord *et al*, 2018; Tsakiridis *et al*, 2021). For instance, metformin has been reported to promote the survival of estrogen-deprived tumor cells through eliciting fatty acid oxidation-driven mitochondrial respiration, indicating the activation of alternative pro-survival mechanisms in metformin resistance (Hampsch *et al*, 2020). In line with this, our present study demonstrates that metformin adaptation was attributed to reactivation of mitochondrial OXPHOS. Particularly, mitochondrial OXPHOS endowed cancer cells with metabolic flexibility to survive and metastasize upon metformin treatment. This observation is supported by the recently published clinical studies, in which reactivated OXPHOS was associated with metformin resistance (Liu *et al*, 2016; Lord *et al*, 2018). Moreover, it has been reported that mitochondrial defects, including mutations of complex subunit genes, might be the most likely determinant of drug resistance to metformin (Birsoy *et al*, 2014). Therefore, blocking the reactivated OXPHOS or interdicting mitochondrial function may hold the potential to sensitize metabolic-adaptive cells to metformin. However, further investigations are needed to support the clinical management of metformin adaptation.

There also exist other concerns about the clinical application of metformin, including the applicable cancer patient cohorts, the therapeutic dosage, and molecular mechanisms underlying the therapeutic effect of metformin. For example, tumor patients with diabetes receiving metformin and chemotherapeutics have higher pathologic complete response rates and improved overall survival rates than nondiabetic groups (Jiralerspong *et al*, 2009; Sonnenblick *et al*, 2017). These different outcomes of metformin treatment might be due to the distinct metabolic patterns of different models, as the above findings were from diabetic patients, while our results, together with other similar conclusions, are extracted from laboratory models or in non-diabetic patients. Importantly, the doses of metformin used in laboratory models (*in vitro* cell lines and *in vivo* mouse models) were 10- to 1,000-fold higher than those deemed safe clinically (Dowling *et al*, 2012; Wang *et al*, 2019). In view of this, it is essential to explore the acceptable antitumor doses of metformin. Assessing the efficacy and safety of higher doses of metformin is also important to determine its clinical availability.

Previous studies have demonstrated the significantly positive association between the upregulated TOMM34 expression and poor outcome of tumor patients (Ahmed *et al*, 2020). In the present work, we found that TOMM34 plays a key role in supporting OXPHOS and subsequent metabolic adaptation of HCC cells under chronic metformin treatment. Tumor cells with a compensatory increase of TOMM34 protein levels following metformin treatment exhibited an increase of OXPHOS, resulting in enhanced metastatic potential. Impairment of TOMM34 expression dramatically decreased the metastatic potential of both metformin-adaptive and parental HCC cells *in vivo* and *in vitro*, suggesting that TOMM34 is a previously unrecognized factor responsible for metformin adaptation and metastasis of HCC. This is somewhat similar to the scenario for TOMM70, which has been reported to participate in cold-stress response by promoting cristae formation and respiration (Latorre-Muro *et al*, 2021). Additionally, PGC-1α, a master regulator of mitochondrial biogenesis, has been found to enhance bioenergetic potential thus facilitating metformin adaptation (Andrzejewski *et al*, 2017). These studies together with our findings indicated that reactivation of mitochondrial OXPHOS is essential for stress adaptation, and targeting this process holds the potential for improving the therapeutic effect for tumor patients.

ATP synthase F1 β subunit (ATP5B) is one of the master catalytic subunits of mitochondrial ATP synthase and acts as an oncogene to promote the growth, metastasis, and drug resistance of tumors (Xu & Li, 2016; Gale *et al*, 2020; Wang *et al*, 2021). Based on the interaction proteomics analysis, we found that ATP5B interacted with TOMM34 and participated in TOMM34-maintained OXPHOS. TOMM34 deficiency impaired the mitochondrial inner membrane system and ATP synthase, resulting in decreased activity of complex I and V. Moreover, silencing ATP5B neutralized TOMM34-promoted ATP production and tumor metastasis, suggesting the indispensable role of ATP5B in TOMM34-mediated metformin adaptation. Moreover, we found that Gboxin, an F_1F_O ATP synthase inhibitor, could disturb the interaction between TOMM34 and ATP5B, leading to impairment of the compensatory adaptive process and tumor metastasis in both HCC cell lines and PDX models. Together with the fact that Gboxin suppressed the proliferation of various cancer cell lines, the present data indicated that Gboxin might be a promising strategy to overcome drug resistance.

In summary, our present study found that TOMM34/ATP5B-mediated metabolic adaptation conferred HCC cells with resilience to long-term exposure of metformin. This compensatory adaptive process enabled the survival and metastasis of tumor cells under metabolic stress. Inhibition of TOMM34-ATP5B interaction by Gboxin weakened mitochondrial OXPHOS and abrogated metformin adaptation, indicating the potential clinical value of the combinational use of Gboxin and metformin in the treatment of liver cancer.

Materials and Methods

Study approval

All experimental procedures regarding human tissue were approved by the Research Ethics Board of the West China Hospital of Sichuan University (2019 (338); 2020 (374)). All animal experiments were performed in accordance with the National Institutes of Health guidelines for the care and use of animals in research and approved by the Institutional Animal Care and Use Committee at West China Hospital of Stomatology, Sichuan University.

Reagents and antibodies

Metformin (cat# S5958), Gboxin (cat# S8828) and S-Gboxin (cat# S0096) were purchased from Selleck; Protein A/G beads were purchased from GE (GE Healthcare, cat# 17-0963-03) or Millipore (Millipore, cat# 16-266); Anti-FLAG® M2 Affinity Gel (cat# A2220) was purchased from Sigma-Aldrich. The antibodies used for immunoblotting or otherwise noted are listed as follows: anti-β-actin (cat# sc-69879, 1:2,000 dilution) was purchased from Santa Cruz Biotechnology; anti-TOMM34 (cat# ab230103, 1:1,000 dilution) was purchased from Abcam; anti-Myc-tag (cat# 2278, 1:1,000 dilution), anti-ZO-1 (cat# 9782, 1:1,000 dilution), anti-E-cadherin

(cat# 9782, 1:1,000 dilution), anti-Vimentin (cat# 9782, 1:2,000 dilution), anti-Claudin-1 (cat# 9782, 1:1,000 dilution), and anti-FLAG-tag (cat# 14793 S, 1:1,000 dilution) were purchased from Cell Signaling Technology; anti-ATP5B (cat# A5769, 1:2,000 dilution), anti-MT-ND1 (cat# A18316, 1:2,000 dilution), anti-MT-ND2 (cat# A17968, 1:2,000 dilution), anti-MT-ND3 (cat# A17969, 1:2,000 dilution), anti-MT-ATP6 (cat# A17960, 1:2,000 dilution), anti-MT-ATP8 (cat# A17890, 1:2,000 dilution), and anti-MT-CO3 (cat# A17891, 1:2,000 dilution) were purchased from ABclonal Technology.

HCC specimens and cell lines

Human HCC tissues were obtained from West China Hospital and Sichuan Province People's Hospital (Chengdu, China) with informed consent and approved by the Institutional Ethics Committee of Sichuan University. Patients with a known synchronous cancer diagnosis or other cancer diagnosis within 5 years of the operation were excluded. Data including clinical information and pathologic characteristics were obtained from medical records. The tissue chips were purchased from Xinchao Biotechnology Co., Ltd. (Shanghai). Informed consent was obtained from all subjects and that the experiments conformed to the principles set out in the WMA Declaration of Helsinki and the Department of Health and Human Services Belmont Report. Human liver cancer cells: PLC/PRF/5 cell line was obtained from American Type Culture Collection (ATCC), Huh7 cell line was purchased from Cell Bank of the Institute of Culture Collection of the Chinese Academy of Sciences, MHCC97H cell line was purchased from Shanghai Zhongqiao Xinzhou Biotechnology Co., LTD. They were all maintained in Dulbecco's modified Eagle's medium (Gibco) supplemented with 10% fetal bovine serum (BI), 100 U/ml penicillin, and 100 μg/ml streptomycin (Invitrogen). All the cell lines were authenticated by short-tandem repeat analysis and subjected to mycoplasma infection test using Mycoplasma PCR Detection Kit (Beyotime Biotechnology, China). Metformin-adaptive Huh7 and PLC/PRF/5 HCC cells were established according previously reported with minor changes (Andrzejewski *et al*, 2017). Briefly, parental HCC cells were treated with gradually increased concentrations of metformin over at least 12 days, the metformin-adaptive population was maintained in the medium containing 5 mM metformin.

Establishment of stable knockdown and overexpressed cells

Stable knockdown of target genes was achieved by lentiviral-based short-hairpin RNA (shRNA) delivery. TOMM34 stable overexpressed Huh7 cells were generated by using the pLenti6.3 expression vectors. The shRNAs and single guided RNAs used in this study are listed below.

Oligonucleotides

TOMM34 (Human) shRNA-1#:

Forward oligo1 (3′UTR): 5′CCGGCCGGGCTTCATAGTTCTTTGTCTCGAGACAAAGAACTATGAAGCCCGGTTTTTTG 3; Reverse oligo1 (5′UTR): 5′AATTCAAAAAACCGGGCTTCATAGTTCTTTGTCTCGAGACAAAGAACTATGAAGCCCGG 3′

TOMM34 (Human) shRNA-2#:

Forward oligo2 (3′UTR): 5′CCGGCGGGCTTCATAGTTCTTTGTACTCGAGTACAAAGAACTATGAAGCCCGTTTTTTG 3; Reverse oligo2 (5′UTR): 5′AATTCAAAAAACGGGCTTCATAGTTCTTTGTACTCGAGTACAAAGAACTATGAAGCCCG 3′

TOMM34 cDNA was amplified and inserted into the FLAG-tagged viral plasmid followed by packaging and infection to overexpress TOMM34 protein in PLC/PRF/5 cells.

ATP5B plasmid was purchased from Shanghai Yile Biological Technology Co., Ltd.

In vivo tail vein injection and orthotopic implantation model

For the tail vein injection model, 1×10^6 TOMM34 stable knockdown Huh7 cells or TOMM34 overexpression PLC/PRF/5 cells, as well as their corresponding control cells, were injected into male BALB/c-nude mice of 6-week-old through the tail vein. For orthotopic implantation, 2.5×10^5 wild-type and TOMM34 stable knockout Huh7 cells, wild-type and TOMM34 stable overexpression PLC/PRF/5 cells were injected orthotopically into the left liver lobe of nude mice, respectively. After 2 weeks, mice were euthanized, their lungs and livers were separated and photographed. Then, the tissues were fixed in 4% formaldehyde for hematoxylin–eosin (H&E) analysis.

Mitochondrial isolation

Mitochondria were prepared using a Mitochondrial Isolation Kit (Beyotime Biotechnology, C3601) according to the manufacturer's instructions. Briefly, cells (5×10^7) were collected and washed with 5–10 ml of ice-cold PBS, and then centrifuging at 600 *g* for 5 min at 4°C. Sediment cells were sequentially lysed using prechilled mitochondrial extraction buffer with 1 mM PMSF. Cells were homogenized in an ice-cold grinder after incubating on ice for 10 min, and then centrifuged at 600 *g* for 10 min at 4°C. The supernatant was further centrifuged at 10,000 *g* at 4°C for 15 min to obtain the mitochondrial fraction.

Mitochondrial DNA (mtDNA) isolation

mtDNA was isolated using Mitochondrial DNA Isolation Kit (Abcam, ab65321) according to the manufacturer's instructions. Briefly, 5×10^7 cells were resuspended in 1 ml cytosol extraction buffer and homogenized in an ice-cold grinder after incubating on ice for 10 min. Then, the supernatant was transferred to a fresh 1.5 ml tube, and centrifuged at 10,000 *g* for 30 min at 4°C. The pellet was resuspended in 1 ml extraction buffer and centrifuged again at 10,000 *g* for 30 min at 4°C. The mitochondria pellet was lysed in 30 μl mitochondrial lysis buffer and kept on ice for 10 min. Five microliter Enzyme Mix was added before incubating at 50°C in a water bath for 60 min until the solution becomes clear. The mixture was centrifuged at 15,000 *g* for 5 min after adding 100 μl absolute ethanol, mixing and incubating at −20°C for 10 min. The pellet is mitochondrial DNA.

Mitochondrial respiratory complex activity assay

Mitochondrial respiratory complex activity was analyzed using Mitochondrial Respiratory Chain Complex Activity Assaying Kit (Solarbio, BC0510, BC3230, BC3240, BC0940, BC1440). 5×10^7 cells were resuspended in 1.0 ml extraction buffer and homogenized in an ice-cold grinder. Then, cells were centrifuged at 600 *g*

 © 2022 The Authors

for 10 min at 4°C before transferring the supernatant to a fresh 1.5 ml tube for further centrifuging at 10,000 *g* at 4°C for 15 min to obtain the mitochondrial fraction. For complex I, the pellet was resuspended with 400 μl of extraction buffer and then ground in an ultrasonic cell disruptor. The homogenate was used for the complex I activity analysis and protein quantitation. Complex I activity was measured by the decrease of absorbance at 340 nm. Complex II activity was calculated by measuring the decrease rate of 2,6-dichlorophenol. Complex III and complex IV activity was calculated by detecting the absorption at 550 nm of reduced cytochrome C. For complex V, the pellet was resuspended in 600 μl of reagent I and then ground in an ultrasonic cell disruptor. The homogenate was used for the complex V activity analysis and protein quantitation. Complex V activity was measured at 660 nm by measuring the rate of Pi increase.

Seahorse XF24 respirometry

The Oxygen Consumption Rate (OCR) and Extracellular Acidification Rate (ECAR) were measured using a Mito Stress Test Kit and XF24 Extracellular Flux Analyzer (Seahorse Bioscience) according to the manufacturer's protocol. In brief, 150,000 cells were plated in 1 ml standard growing medium and cultured overnight. Then, cells were washed with XF media (1% FBS) and incubated in a CO_2-free incubator at 37°C for 2 h to establish equilibration prior to loading. OCR and ECAR of HCC cells were measured under XF Base Medium containing 2 mM glutamine (pH was adjusted to 7.40 ± 0.05 at 37°C) after injection of 5 μM oligomycin, 3 μM FCCP, 1 μM antimycin A and rotenone using a Seahorse XF Analyzer.

Patient-derived xenograft (PDX)

Denotes PDX passage 2 (P2) male NSG mice were purchased from BEIJING IDMO Co., Ltd. Mice were sacrificed by euthanasia when tumors reached a volume of 800–1,000 mm^3. Then tumor tissues were collected and, cut into ~3 mm^3 pieces and implanted subcutaneously in male NSG mice. For the metformin adaptation models, mice were randomly divided into two groups and received vehicle or metformin treatment for 17–22 days at escalating dose levels (150 mg/kg/day for days 1–7, 200 mg/kg/day for days 8–14, and 250 mg/kg/day thereafter) once the tumor volumes reached ~100 mm^3, which was blinded to the investigators who collected the samples and performed analyses. Then, mice were euthanized for analysis, tissues were harvested and fixed in 4% formalin immediately. For validating the combinational effect of Gboxin and metformin, mice were randomly divided into three groups and received indicated treatment for 10 days (vehicle group, 200 mg/kg/day metformin treatment, combinational treatment with 200 mg/kg/day metformin and 10 mg/kg/day S-Gboxin). Mice were euthanized for analysis, tissues were harvested and fixed in 4% formalin immediately.

Subcutaneously implanted tumor model

Male BALB/c nude mice at 6 weeks were purchased from GemPharmatech Co, Ltd. 1×10^7 adaptive Huh7 cells were suspended in 100 μl PBS and injected subcutaneously into the flanks of the BALB/c nude mice. When the tumor volume reached ~100 mm^3, mice were randomly divided into four groups and administered with 0.1 ml of vehicle, S-Gboxin (10 mg/kg/day), metformin (200 mg/kg/day), or combination of S-Gboxin and metformin for 14 days (Oral gavage every day). Then mice were euthanized, and tumor tissues were harvested.

Transwell migration and invasion assay

The transwell migration and invasion assays were performed using 8.0 μm pore polycarbonate membrane inserts (Corning, cat# 3422) according to the manufacturer's instructions. Briefly, cells were resuspended by FBS-free starvation medium and then planted in the inserts, and growth medium was added outside the chamber in the wells of the plate. For invasion assay, the insert was pre-coated with matrigel (BD Biosciences, cat# 356234) and cells were seeded in each chamber. The cells were incubated at 37°C for 36 h and then fixed by 4% formaldehyde and stained with 0.1% crystal violet. Migrated cells were visualized using a DM2500 fluorescence microscope (Leica).

Wound healing assay

About 2×10^5 cells were seeded in 12-wells plate and incubated at 37°C overnight. A "wound" in the cell monolayer was created using a pipette tip Images were captured immediately and at regular intervals during cell migration to close the wound. Comparing the images to quantify the migrated distance.

Co-immunoprecipitation (Co-IP)

Cells were collected and lysed using 1 ml IP lysis buffer (100 mM NaCl, 20 mM Tris–HCl, pH 7.5, 0.5 mM EDTA, 0.1% NP-40) with complete protease and phosphatase inhibitor on ice for 30 min. And then centrifuged at 4,000 *g* at 4°C for 10 min to obtain the supernatant fraction. One hundred microliter of the supernatant was transferred to a new tube as the control after centrifugation, and the remaining supernatant was incubated with 1 μg of IgG, anti-Myc, anti-TOMM34 antibody or Anti-FLAG® M2 Affinity Gel (cat# A2220, Sigma-Aldrich) as indicated overnight at 4°C. For those samples incubated with antibody, 30 μl of protein A/G agarose beads (GE Healthcare, cat# 17-0963-03) were then added and left for another 2 h at 4°C after washing three times with IP lysis buffer. After three washes using washing buffer (150 mM NaCl, 0.5 mM EDTA, 20 mM Tris–HCl, pH 7.4, 0.5% NP-40), the beads were recovered by centrifugation on 500 *g* for 3 min and eluted by SDS-PAGE loading buffer. The resultant eluates were subjected to immunoblotting analysis.

Mass spectrometry (MS)

Anti-FLAG® M2 Affinity Gel (cat# A2220, Sigma-Aldrich) were incubated with cell lysates according to the manufacturer's instructions. Immunoprecipitated proteins were subjected to SDS-PAGE and sample containing areas were cut into small pieces. After dehydration with acetonitrile (Fisher Scientific, cat# A955-4), reduction with 10 mM 1,4-dithiothreitol (Sigma-Aldrich, cat# D0632), alkylation with 55 mM iodoacetamide (Sigma-Aldrich, cat# V900335) and digestion with trypsin (Promega, cat# V5071) overnight, peptides were extracted from the gel particles and purified using ZipTips

© 2022 The Authors

(Millipore, cat# ZTC18S096) before MS analysis. The MS analysis and data processing were performed as described in our previous study (Jiang *et al*, 2020).

TMA-based immunohistochemistry

Briefly, the human HCC tissue microarray (TMA) was deparaffinized with xylene and ethanol and further blocked by 3% H_2O_2 for 10 min at room temperature. Then, the TMA was incubated in retrieval buffer and boiled for 3.5 min. After three washes with PBS, the TMA was blocked with 5% normal serum and incubated with primary antibody at 4°C overnight. Next, TMA was treated with MaxVision HRP solution (MXB Biotechnology, cat# 5020) for 60 min, followed by staining with DAB Peroxidase Substrate (MXB Biotechnology, cat# 0031). The EnVision Detection System (Agilent Technologies, K5007) was used to detect antigen expression.

Detection of circulating tumor cell (CTC)

CTC was detected by RT-qPCR using human-specific short interspersed elements (Primers: Forward-5′-GGTGAAACCCCGTCTCT ACT-3′; Reverse-5′-GGTTCAAGCGATTCTCCTGC-3′) according to a previous report (Funakoshi *et al*, 2017).

Intracellular ATP measurement

Intracellular ATP levels were detected using the bioluminescence method with an ATP Detection Kit (Beyotime Biotechnology, S0027) according to the manufacturer's instructions. Briefly, the cells (2×10^4) were seeded in six-well plates for 24 h and then washed with PBS before adding 200 µl lysis buffer. After lysing on ice for 30 min, the six-well plate was centrifuged at 12,000 *g* at 4°C for 5 min to obtain the supernatant fraction. Then, 100 µl of ATP detection working solution was added to the detection tube and left at room temperature for 3–5 min to consume all the background ATP. Next, 20 µl sample or standard were added to the detection tube, quickly mixed and measured for relative light unit (RLU) value using a Chemiluminometer (luminometer) or liquid scintillation meter.

Immunofluorescence

Cells were seeded on the glass cover slides (WHB scientific, cat# whb-24-cs) overnight. After being fixed by 4% formaldehyde, permeabilized with 0.3% Triton X-100 and blocked with 5% BSA, the slides were then incubated with indicated antibodies (1:100 dilution) at 4°C overnight. Then, slides were incubated with Alexa Fluor 488/594–conjugated goat anti-rabbit IgG (1:200 dilution) at room temperature for 1 h and nuclei were stained with DAPI solution (Solarbio, cat# C0060, 1:2,000 dilution) at room temperature for 10 min. Images were captured using confocal laser scanning microscopy (Carl Zeiss Microimaging).

RNA-Seq

Total RNA extraction was performed with TRIzol Reagent (Invitrogen, Carlsbad, CA, USA). RNA-sequencing analysis was performed at Novogene (Tianjin, China). For analysis of sequencing results, the differentially expressed genes were identified under threshold:

The paper explained

Problem

Metformin, a well-known antidiabetic drug, has been repurposed for cancer treatment. However, recently observed drug resistance and enhanced tumor metastasis have questioned its further application. Therefore, investigating the mechanisms underlying metformin adaptation and exploring a combinational strategy may lead to better clinical outcome for cancer treatment.

Results

We found that long-term metformin exposure led to metabolic adaptation of HCC cells, manifesting as an obvious epithelial–mesenchymal transition (EMT) phenotype and compensatory elevation of ATPase activity. TOMM34, a translocase of the outer mitochondrial membrane, was identified to promote tumor metastasis in response to long-term metformin treatment. Mechanistically, TOMM34 bound to ATP5B and preserved ATPase activity, thus enhancing mitochondrial oxidative phosphorylation (OXPHOS) and ATP production. Notably, disturbing the interaction between TOMM34 and ATP5B with Gboxin, a specific OXPHOS inhibitor, impaired metformin adaptation and suppressed tumor growth and metastasis both *in vitro* and *in vivo*.

Impact

We highlight the clinic value of TOMM34/ATP5B as potential targets for HCC therapy. We also provide evidence for the potential application of the combinational use of Gboxin and metformin in HCC treatment.

P-value ≤ 0.05 and log2 (fold change) ≥ 1. KEGG pathway enrichment analysis was performed using KOBAS (http://kobas.cbi.pku.edu.cn/index.php).

Statistical analysis

All data and error bars are presented as the mean ± SD from at least three independent experiments. Comparisons between two groups were performed by two-tailed Student's *t*-test or one-way analysis of variance (ANOVA). Comparisons of repeated measurements over time were performed using two-way ANOVA. Survival curves were generated using the Kaplan–Meier method and evaluated by the log-rank (Mantel-Cox) test. Pearson's Chi-square test was used to investigate the correlation between two independent groups. The P-value was calculated using GraphPad (version 8.00). $P < 0.05$ was considered statistically significant. Image analysis was performed by ImageJ, ZENBLACK or Case Viewer software.

Data availability

The datasets produced in this study are available in the following databases:

- RNA-Seq data generated in this study has been deposited in the SRA database: PRJNA861118 (https://www.ncbi.nlm.nih.gov/bioproject/PRJNA861118).
- Protein interaction and label-free AP-MS data: IPXD035365 (http://proteomecentral.proteomexchange.org/cgi/GetDataset?ID=PXD035365; https://www.iprox.cn/page/project.html?id=IPX0004716000).

Expanded View for this article is available online.

© 2022 The Authors

Acknowledgements
We thank Prof. Rui Liu for providing valuable suggestions on the manuscript and study design. We thank Prof. Lunzhi Dai and his students Yanqiu Gong, Yu Liu, Zhili Xia for the technical assistance for MS analysis. This work was supported by grants from the National Natural Science Foundation of China (81821002, 81790251, 82130082, 82003098), National Key Research and Development Project of China 2020YFA0509400, Guangdong Basic and Applied Basic Research Foundation (2019B030302012), and 1·3·5 project for disciplines of excellence (ZYGD22007, ZYJC21004).

Author contributions
Ping Jin: Data curation; software; validation; investigation; visualization; methodology; writing – original draft. **Jingwen Jiang:** Conceptualization; resources; data curation; formal analysis; visualization; methodology; writing – original draft. **Li Zhou:** Conceptualization; data curation; software; investigation; writing – original draft. **Zhao Huang:** Conceptualization; data curation; software; investigation; methodology; writing – original draft. **Siyuan Qin:** Resources; data curation; software; investigation; visualization. **Hai-Ning Chen:** Resources; data curation; software; methodology. **Liyuan Peng:** Data curation; validation; investigation; methodology. **Zhe Zhang:** Validation; investigation; methodology. **Bowen Li:** Software; formal analysis; visualization; methodology. **Maochao Luo:** Resources; software; visualization; methodology. **Tingting Zhang:** Data curation; methodology. **Hui Ming:** Data curation; software. **Ning Ding:** Formal analysis; investigation. **Lei Li:** Software; project administration. **Na Xie:** Resources; software; visualization; methodology. **Wei Gao:** Resources; data curation; software; formal analysis; methodology. **Wei Zhang:** Resources; data curation; validation; project administration. **Edouard C Nice:** Resources; software; writing – review and editing. **Yuquan Wei:** Conceptualization; resources; supervision; project administration. **Canhua Huang:** Conceptualization; resources; data curation; supervision; funding acquisition; project administration; writing – review and editing.

Disclosure and competing interests statement
The authors declare that they have no conflict of interest.

References

Ahmed MAH, Ali MH, Abbas HH, Elatrash GA, Foda AAM (2020) Expression of TOMM34 and its clinicopathological correlations in urothelial carcinoma of the bladder. *Pathol Oncol Res* 26: 411 – 418

Aleskandarany MA, Negm OH, Rakha EA, Ahmed MA, Nolan CC, Ball GR, Caldas C, Green AR, Tighe PJ, Ellis IO (2012) TOMM34 expression in early invasive breast cancer: a biomarker associated with poor outcome. *Breast Cancer Res Treat* 136: 419 – 427

Andrzejewski S, Klimcakova E, Johnson RM, Tabaries S, Annis MG, McGuirk S, Northey JJ, Chenard V, Sriram U, Papadopoli DJ *et al* (2017) PGC-1alpha promotes breast cancer metastasis and confers bioenergetic flexibility against metabolic drugs. *Cell Metab* 26: e775

Ashton TM, McKenna WG, Kunz-Schughart LA, Higgins GS (2018) Oxidative phosphorylation as an emerging target in cancer therapy. *Clin Cancer Res* 24: 2482 – 2490

Birsoy K, Possemato R, Lorbeer FK, Bayraktar EC, Thiru P, Yucel B, Wang T, Chen WW, Clish CB, Sabatini DM (2014) Metabolic determinants of cancer cell sensitivity to glucose limitation and biguanides. *Nature* 508: 108 – 112

Bo S, Ciccone G, Rosato R, Villois P, Appendino G, Ghigo E, Grassi G (2012) Cancer mortality reduction and metformin: a retrospective cohort study in type 2 diabetic patients. *Diabetes Obes Metab* 14: 23 – 29

Bosc C, Selak MA, Sarry JE (2017) Resistance is futile: targeting mitochondrial energetics and metabolism to overcome drug resistance in cancer treatment. *Cell Metab* 26: 705 – 707

Burke PJ (2017) Mitochondria, bioenergetics and apoptosis in cancer. *Trends Cancer* 3: 857 – 870

Cai Z, Li CF, Han F, Liu C, Zhang A, Hsu CC, Peng D, Zhang X, Jin G, Rezaeian AH *et al* (2020) Phosphorylation of PDHA by AMPK drives TCA cycle to promote cancer metastasis. *Mol Cell* 80: 263 – 278

Chen CL, Uthaya Kumar DB, Punj V, Xu J, Sher L, Tahara SM, Hess S, Machida K (2016) NANOG metabolically reprograms tumor-initiating stem-like cells through tumorigenic changes in oxidative phosphorylation and fatty acid metabolism. *Cell Metab* 23: 206 – 219

Chen Q, Lei JH, Bao J, Wang H, Hao W, Li L, Peng C, Masuda T, Miao K, Xu J *et al* (2020) BRCA1 deficiency impairs mitophagy and promotes inflammasome activation and mammary tumor metastasis. *Adv Sci (Weinh)* 7: 1903616

Chen HN, Shu Y, Liao F, Liao X, Zhang H, Qin Y, Wang Z, Luo M, Liu Q, Xue Z *et al* (2021) Genomic evolution and diverse models of systemic metastases in colorectal cancer. *Gut* 71: 322 – 332

Coyle C, Cafferty FH, Vale C, Langley RE (2016) Metformin as an adjuvant treatment for cancer: a systematic review and meta-analysis. *Ann Oncol* 27: 2184 – 2195

De A, Kuppusamy G (2020) Metformin in breast cancer: preclinical and clinical evidence. *Curr Probl Cancer* 44: 100488

Dowling RJ, Niraula S, Stambolic V, Goodwin PJ (2012) Metformin in cancer: translational challenges. *J Mol Endocrinol* 48: R31 – R43

Eisner V, Picard M, Hajnoczky G (2018) Mitochondrial dynamics in adaptive and maladaptive cellular stress responses. *Nat Cell Biol* 20: 755 – 765

Feng YX, Wang T, Deng YZ, Yang P, Li JJ, Guan DX, Yao F, Zhu YQ, Qin Y, Wang H *et al* (2011) Sorafenib suppresses postsurgical recurrence and metastasis of hepatocellular carcinoma in an orthotopic mouse model. *Hepatology* 53: 483 – 492

Funakoshi K, Bagheri M, Zhou M, Suzuki R, Abe H, Akashi H (2017) Highly sensitive and specific Alu-based quantification of human cells among rodent cells. *Sci Rep* 7: 13202

Gale M, Li Y, Cao J, Liu ZZ, Holmbeck MA, Zhang M, Lang SM, Wu L, Do Carmo M, Gupta S *et al* (2020) Acquired resistance to HER2-targeted therapies creates vulnerability to ATP synthase inhibition. *Cancer Res* 80: 524 – 535

Gomkale R, Cruz-Zaragoza LD, Suppanz I, Guiard B, Montoya J, Callegari S, Pacheu-Grau D, Warscheid B, Rehling P (2020) Defining the substrate spectrum of the TIM22 complex identifies pyruvate carrier subunits as unconventional cargos. *Curr Biol* 30: 1119 – 1127

Gore E, Duparc T, Genoux A, Perret B, Najib S, Martinez LO (2022) The multifaceted ATPase inhibitory factor 1 (IF1) in energy metabolism reprogramming and mitochondrial dysfunction: a new player in age-associated disorders? *Antioxid Redox Signal* 37: 370 – 393

Hampsch RA, Wells JD, Traphagen NA, McCleery CF, Fields JL, Shee K, Dillon LM, Pooler DB, Lewis LD, Demidenko E *et al* (2020) AMPK activation by metformin promotes survival of dormant ER(+) breast cancer cells. *Clin Cancer Res* 26: 3707 – 3719

Herkenne S, Ek O, Zamberlan M, Pellattiero A, Chergova M, Chivite I, Novotna E, Rigoni G, Fonseca TB, Samardzic D *et al* (2020) Developmental and tumor angiogenesis requires the mitochondria-shaping protein Opa1. *Cell Metab* 31: 987 – 1003

© 2022 The Authors

Jiang J, Zhang L, Chen H, Lei Y, Zhang T, Wang Y, Jin P, Lan J, Zhou L, Huang Z *et al* (2020) Regorafenib induces lethal autophagy arrest by stabilizing PSAT1 in glioblastoma. *Autophagy* 16: 106 – 122

Jin P, Jiang J, Zhou L, Huang Z, Nice EC, Huang C, Fu L (2022) Mitochondrial adaptation in cancer drug resistance: prevalence, mechanisms, and management. *J Hematol Oncol* 15: 97

Jiralerspong S, Palla SL, Giordano SH, Meric-Bernstam F, Liedtke C, Barnett CM, Hsu L, Hung MC, Hortobagyi GN, Gonzalez-Angulo AM (2009) Metformin and pathologic complete responses to neoadjuvant chemotherapy in diabetic patients with breast cancer. *J Clin Oncol* 27: 3297 – 3302

Jourdain AA, Begg BE, Mick E, Shah H, Calvo SE, Skinner OS, Sharma R, Blue SM, Yeo GW, Burge CB *et al* (2021) Loss of LUC7L2 and U1 snRNP subunits shifts energy metabolism from glycolysis to OXPHOS. *Mol Cell* 81: 1905 – 1919

Kowalik MA, Puliga E, Cabras L, Sulas P, Petrelli A, Perra A, Ledda-Columbano GM, Morandi A, Merlin S, Orru C *et al* (2020) Thyroid hormone inhibits hepatocellular carcinoma progression via induction of differentiation and metabolic reprogramming. *J Hepatol* 72: 1159 – 1169

Latorre-Muro P, O'Malley KE, Bennett CF, Perry EA, Balsa E, Tavares CDJ, Jedrychowski M, Gygi SP, Puigserver P (2021) A cold-stress-inducible PERK/OGT axis controls TOM70-assisted mitochondrial protein import and cristae formation. *Cell Metab* 33: 598 – 614

LeBleu VS, O'Connell JT, Gonzalez Herrera KN, Wikman H, Pantel K, Haigis MC, de Carvalho FM, Damascena A, Domingos Chinen LT, Rocha RM *et al* (2014) PGC-1alpha mediates mitochondrial biogenesis and oxidative phosphorylation in cancer cells to promote metastasis. *Nat Cell Biol* 16: 1001 – 1015

Lisowski P, Kannan P, Mlody B, Prigione A (2018) Mitochondria and the dynamic control of stem cell homeostasis. *EMBO Rep* 19: e45432

Liu W, Duan X, Fang X, Shang W, Tong C (2018) Mitochondrial protein import regulates cytosolic protein homeostasis and neuronal integrity. *Autophagy* 14: 1293 – 1309

Liu X, Romero IL, Litchfield LM, Lengyel E, Locasale JW (2016) Metformin targets central carbon metabolism and reveals mitochondrial requirements in human cancers. *Cell Metab* 24: 728 – 739

Liu R, Li J, Shao J, Lee J-H, Qiu X, Xiao Y, Zhang B, Hao Y, Li M, Chen Q (2021). Innate immune response orchestrates phosphoribosyl pyrophosphate synthetases to support DNA repair. *Cell Metab* 33: 2076 – 2089.e9. https://doi.org/10.1016/j.cmet.2021.07.009

Lord SR, Cheng WC, Liu D, Gaude E, Haider S, Metcalf T, Patel N, Teoh EJ, Gleeson F, Bradley K *et al* (2018) Integrated pharmacodynamic analysis identifies two metabolic adaption pathways to metformin in breast cancer. *Cell Metab* 28: 679 – 688

Nuttall SD, Hanson BJ, Mori M, Hoogenraad NJ (1997) hTom34: a novel translocase for the import of proteins into human mitochondria. *DNA Cell Biol* 16: 1067 – 1074

Park SH, Lee AR, Choi K, Joung S, Yoon JB, Kim S (2019) TOMM20 as a potential therapeutic target of colorectal cancer. *BMB Rep* 52: 712 – 717

Patterson RE, Marinac CR, Sears DD, Kerr J, Hartman SJ, Cadmus-Bertram L, Villasenor A, Flatt SW, Godbole S, Li H *et al* (2018) The effects of metformin and weight loss on biomarkers associated with breast cancer outcomes. *J Natl Cancer Inst* 110: 1239 – 1247

Pitt AS, Buchanan SK (2021) A biochemical and structural understanding of TOM complex interactions and implications for human health and disease. *Cell* 10: 1164

Pollak MN (2012) Investigating metformin for cancer prevention and treatment: the end of the beginning. *Cancer Discov* 2: 778 – 790

Scherbakov AM, Sorokin DV, Tatarskiy VV Jr, Prokhorov NS, Semina SE, Berstein LM, Krasil'nikov MA (2016) The phenomenon of acquired resistance to metformin in breast cancer cells: the interaction of growth pathways and estrogen receptor signaling. *IUBMB Life* 68: 281 – 292

Schito L, Rey S (2018) Cell-autonomous metabolic reprogramming in hypoxia. *Trends Cell Biol* 28: 128 – 142

Shankaraiah RC, Callegari E, Guerriero P, Rimessi A, Pinton P, Gramantieri L, Silini EM, Sabbioni S, Negrini M (2019) Metformin prevents liver tumourigenesis by attenuating fibrosis in a transgenic mouse model of hepatocellular carcinoma. *Oncogene* 38: 7035 – 7045

Shi Y, Lim SK, Liang Q, Iyer SV, Wang HY, Wang Z, Xie X, Sun D, Chen YJ, Tabar V *et al* (2019) Gboxin is an oxidative phosphorylation inhibitor that targets glioblastoma. *Nature* 567: 341 – 346

Sonnenblick A, Agbor-Tarh D, Bradbury I, Di Cosimo S, Azim HA Jr, Fumagalli D, Sarp S, Wolff AC, Andersson M, Kroep J *et al* (2017) Impact of diabetes, insulin, and metformin use on the outcome of patients with human epidermal growth factor receptor 2-positive primary breast cancer: analysis from the ALTTO phase III randomized trial. *J Clin Oncol* 35: 1421 – 1429

Suliman HB, Piantadosi CA (2016) Mitochondrial quality control as a therapeutic target. *Pharmacol Rev* 68: 20 – 48

Toraih EA, Alrefai HG, Hussein MH, Helal GM, Khashana MS, Fawzy MS (2019) Overexpression of heat shock protein HSP90AA1 and translocase of the outer mitochondrial membrane TOM34 in HCV-induced hepatocellular carcinoma: a pilot study. *Clin Biochem* 63: 10 – 17

Trcka F, Durech M, Man P, Hernychova L, Muller P, Vojtesek B (2014) The assembly and intermolecular properties of the Hsp70-Tomm34-Hsp90 molecular chaperone complex. *J Biol Chem* 289: 9887 – 9901

Trcka F, Durech M, Vankova P, Chmelik J, Martinkova V, Hausner J, Kadek A, Marcoux J, Klumpler T, Vojtesek B *et al* (2019) Human stress-inducible Hsp70 has a high propensity to form ATP-dependent antiparallel dimers that are differentially regulated by cochaperone binding. *Mol Cell Proteomics* 18: 320 – 337

Tsakiridis T, Pond GR, Wright J, Ellis PM, Ahmed N, Abdulkarim B, Roa W, Robinson A, Swaminath A, Okawara G *et al* (2021) Metformin in combination with chemoradiotherapy in locally advanced non-small cell lung cancer: the OCOG-ALMERA randomized clinical trial. *JAMA Oncol* 7: 1333 – 1341

Wang Y, An H, Liu T, Qin C, Sesaki H, Guo S, Radovick S, Hussain M, Maheshwari A, Wondisford FE *et al* (2019) Metformin improves mitochondrial respiratory activity through activation of AMPK. *Cell Rep* 29: 1511 – 1523

Wang X, Chang X, He C, Fan Z, Yu Z, Yu B, Wu X, Hou J, Li J, Su L *et al* (2021) ATP5B promotes the metastasis and growth of gastric cancer by activating the FAK/AKT/MMP2 pathway. *FASEB J* 35: e20649

Wheaton WW, Weinberg SE, Hamanaka RB, Soberanes S, Sullivan LB, Anso E, Glasauer A, Dufour E, Mutlu GM, Budigner GS *et al* (2014) Metformin inhibits mitochondrial complex I of cancer cells to reduce tumorigenesis. *Elife* 3: e02242

Wu H, Wei H, Sehgal SA, Liu L, Chen Q (2016) Mitophagy receptors sense stress signals and couple mitochondrial dynamic machinery for mitochondrial quality control. *Free Radic Biol Med* 100: 199 – 209

Xu G, Li JY (2016) ATP5A1 and ATP5B are highly expressed in glioblastoma tumor cells and endothelial cells of microvascular proliferation. *J Neurooncol* 126: 405 – 413

 © 2022 The Authors

Yu J, Xu QG, Wang ZG, Yang Y, Zhang L, Ma JZ, Sun SH, Yang F, Zhou WP (2018) Circular RNA cSMARCA5 inhibits growth and metastasis in hepatocellular carcinoma. *J Hepatol* 68: 1214 – 1227

Zhang B, Wang J, Wang X, Zhu J, Liu Q, Shi Z, Chambers MC, Zimmerman LJ, Shaddox KF, Kim S *et al* (2014) Proteogenomic characterization of human colon and rectal cancer. *Nature* 513: 382 – 387

Zhang Z, Qin S, Chen Y, Zhou L, Yang M, Tang Y, Zuo J, Zhang J, Mizokami A, Nice EC *et al* (2022) Inhibition of NPC1L1 disrupts adaptive responses of drug-tolerant persister cells to chemotherapy. *EMBO Mol Med* 14: e14903

Zheng Y, Huang C, Lu L, Yu K, Zhao J, Chen M, Liu L, Sun Q, Lin Z, Zheng J *et al* (2021) STOML2 potentiates metastasis of hepatocellular carcinoma by promoting PINK1-mediated mitophagy and regulates sensitivity to lenvatinib. *J Hematol Oncol* 14: 16

Zhou B, Zhang JY, Liu XS, Chen HZ, Ai YL, Cheng K, Sun RY, Zhou D, Han J, Wu Q (2018) Tom20 senses iron-activated ROS signaling to promote melanoma cell pyroptosis. *Cell Res* 28: 1171 – 1185

License: This is an open access article under the terms of the Creative Commons Attribution License, which permits use, distribution and reproduction in any medium, provided the original work is properly cited.

Expanded View Figures

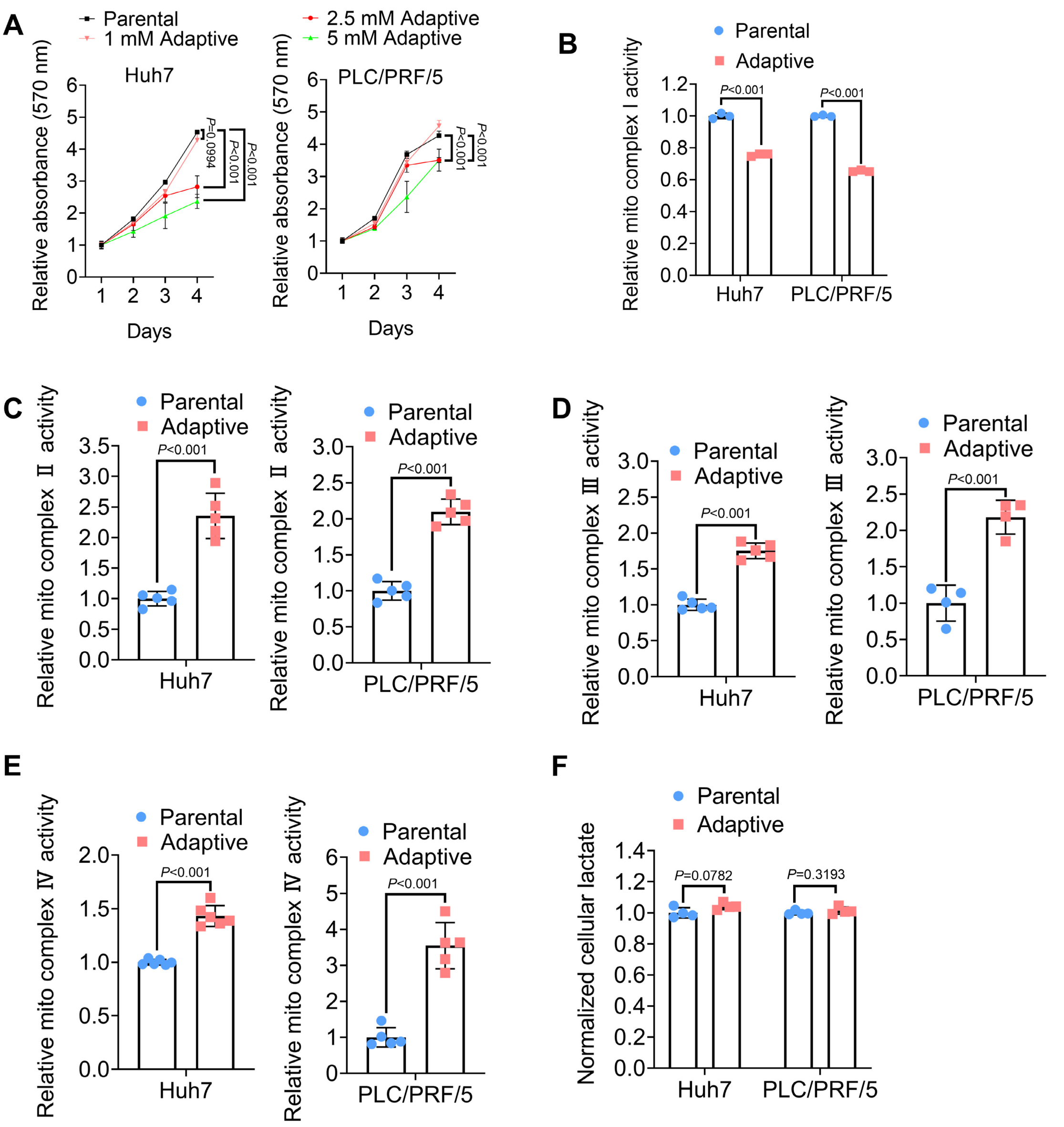

Figure EV1. Metformin-adaptive HCC cells exhibit hyperactivity of mitochondrial respiration.

A MTT assays showing the growth of parental HCC cells and metformin-adaptive HCC cells (obtained from long-term exposure to different concentrations of metformin). (n = 6 biological replicates, Two-way ANOVA).

B–E The activity of mitochondrial complex I, II, III and IV in parental and adaptive HCC cells was measured. (n = 3 technical replicates in B, n = 5 technical replicates in C, n = 5 or 4 technical replicates in D, n = 6 or 5 technical replicates in E, Student's t-test).

F Cellular lactate level of parental and adaptive HCC cells. (n = 4 biological replicates, Two-way ANOVA).

Data information: Data are presented as means ± SD.

© 2022 The Authors

Figure EV2. TOMM34 upregulates the metastatic potential of HCC.

A The protein level of TOMM34 in PDX models with or without long-term metformin treatment was evaluated by immunohistochemistry. Scale bars, (Left) 1,000 μm; (Right) 25 μm. ($n = 5$ biological replicates in each group, Student's t-test).

B Schematic diagram for the HCC lung metastasis model in the orthotopic injection mouse. HE and IHC assays showing the histology and TOMM34 expression. Scale bars, 100 or 25 μm.

C, D Transwell assays showing migration and invasion ability of Huh7 and PLC/PRF/5 cells (5×10^4 Huh7 cells, 1×10^5 PLC/PRF/5 cells; sh*TOM*, sh*TOMM34*, TOMM34 knockdown cells; OE-TOM, OE-TOMM34, TOMM34-overexpressed cells). Scale bars, 100 μm. ($n = 3$ biological replicates, Two-way ANOVA).

E, F Wound healing assay showing migration of HCC cells. Scale bars, 200 μm. ($n = 6$ technical replicates, Student's t-test).

G Alterations in the morphology of HCC cells with or without the overexpression or knockdown of TOMM34 (sh*TOM*, sh*TOMM34*, TOMM34 knockdown cells; OE-TOM, OE-TOMM34, TOMM34-overexpressed cells). Scale bars, 100 μm.

H TOMM34 mRNA levels in HCC patients with or without metastasis according to TCGA data set GSE3500 (https://www.ncbi.nlm.nih.gov/geo/query/acc.cgi?acc=GSE3500). (One-way ANOVA). The boxplot represents the minimum value, the second quartile, the median value, the third quartile, and the maximum value.

I Overall survival of HCC patients from TCGA dataset (https://portal.gdc.cancer.gov/projects/TCGA-LIHC). Patients were stratified into High and Low groups according to their TOMM34 expression levels. Statistical difference was determined using log-rank (Mantel-Cox) test.

Data information: Data are presented as means ± SD.
Source data are available online for this figure.

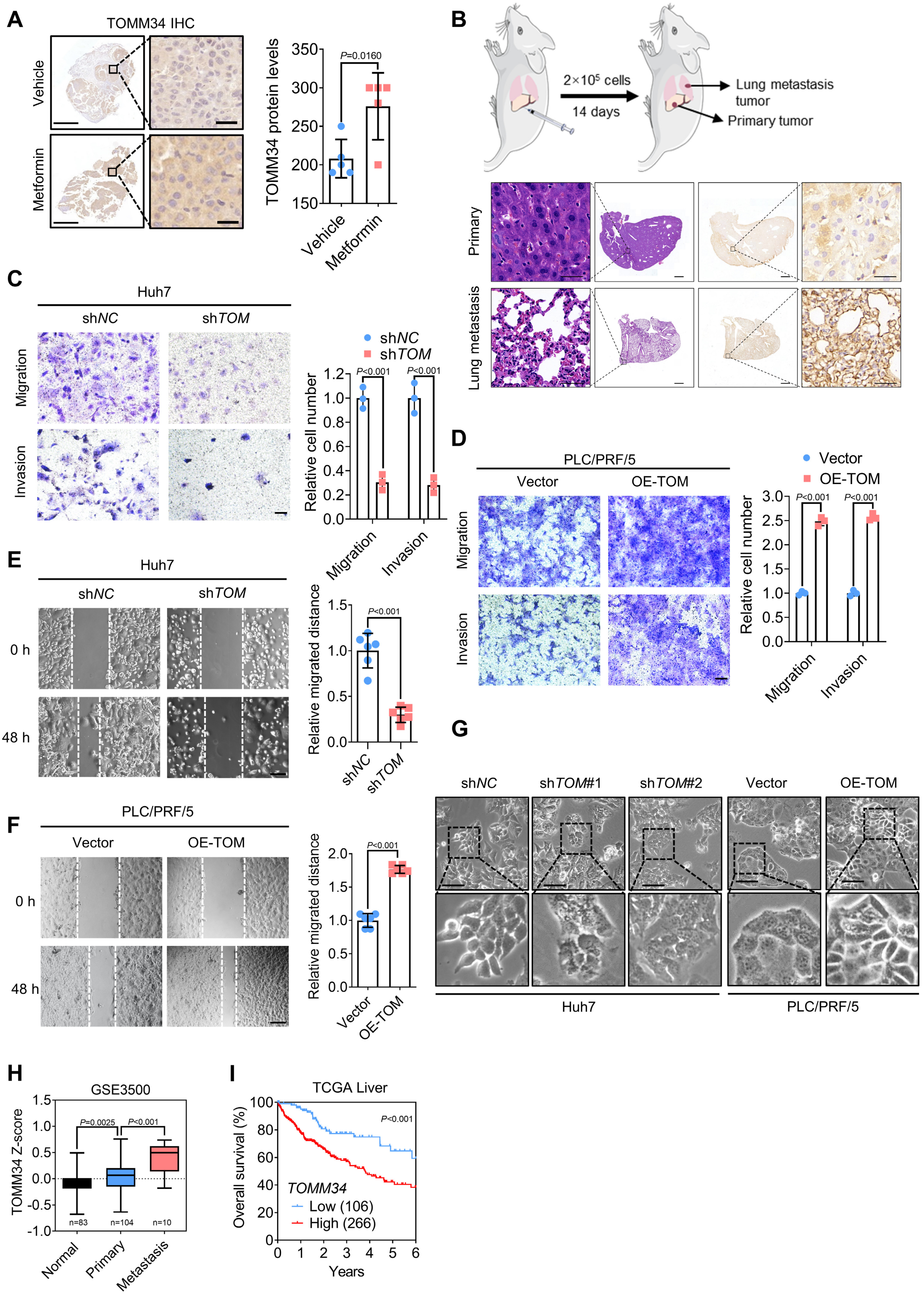

Figure EV2.

 © 2022 The Authors

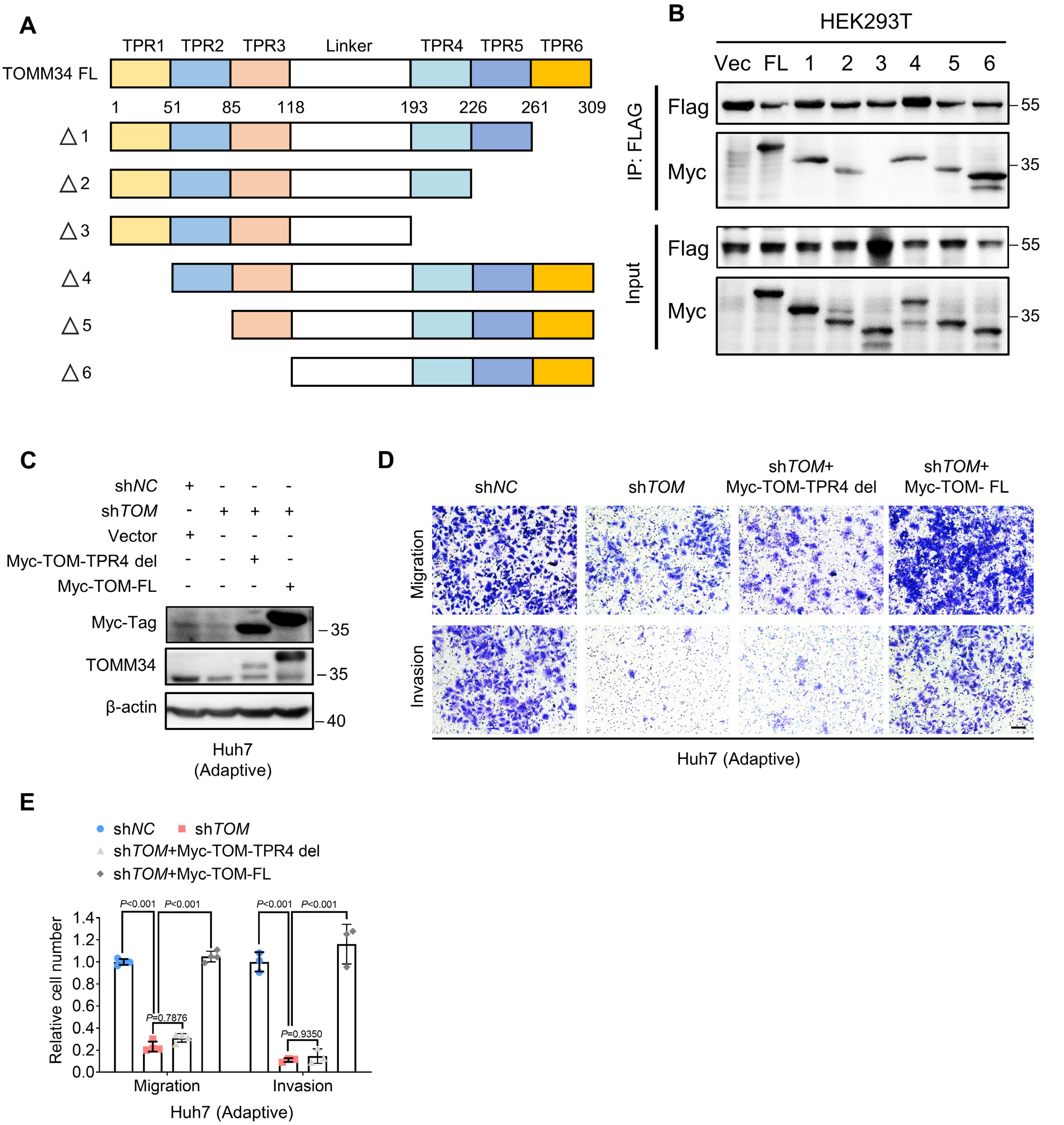

Figure EV3. TPR4 region is required for the interaction of TOMM34 with ATP5B.

A Schematic diagram showing wild-type TOMM34 (FL, full length, 1–309) and its truncations.

B HEK293T cells were transfected with the indicated plasmids, followed by co-IP assays and Western blot to examine their interactions with ATP5B (FL, full length, 1–309).

C Western blot showing the expression of full-length (Myc-TOM-FL) or TPR4-truncated TOMM34 (Myc-TOM-TPR4 del) in adaptive Huh7 sh*TOM* (sh*TOMM34*) cells.

D, E Transwell assays showing the migration and invasion of HCC cells expressing full-length (Myc-TOM-FL) or TPR4-truncated TOMM34 (Myc-TOM-TPR4 del) (5×10^4 cells). Scale bars, 100 μm. ($n = 4$ biological replicates for migration, $n = 3$ biological replicates for invasion, Two-way ANOVA).

Data information: Data are presented as means ± SD.
Source data are available online for this figure.

© 2022 The Authors

Figure EV4. ATP5B is essential for TOMM34-mediated metastasis.

A ATP levels in HCC cells were measured with or without silencing of ATP5B by siRNAs. ($n = 4$ biological replicates in Huh7, $n = 3$ biological replicates in PLC/PRF/5, One-way ANOVA).

B, C Western blot showing the expression of indicated proteins in HCC cells with or without ATP5B silencing (OE-TOM, OE-TOMM34, TOMM34-overexpressed cells).

D, E Transwell assays showing the migration and invasion of parental HCC cells with or without the silence of ATP5B (5×10^4 cells, OE-TOM, OE-TOMM34, TOMM34-overexpressed cells) Scale bars, 100 μm. ($n = 3$ biological replicates, Two-way ANOVA).

F, G Wound healing assay showing the migration of HCC cells transfected with si*Scramble* or si*ATP5B* (OE-TOM, OE-TOMM34, TOMM34-overexpressed cells) Scale bars, 200 μm. ($n = 3$ technical replicates, Two-way ANOVA).

Data information: Data are presented as means ± SD.
Source data are available online for this figure.

 © 2022 The Authors

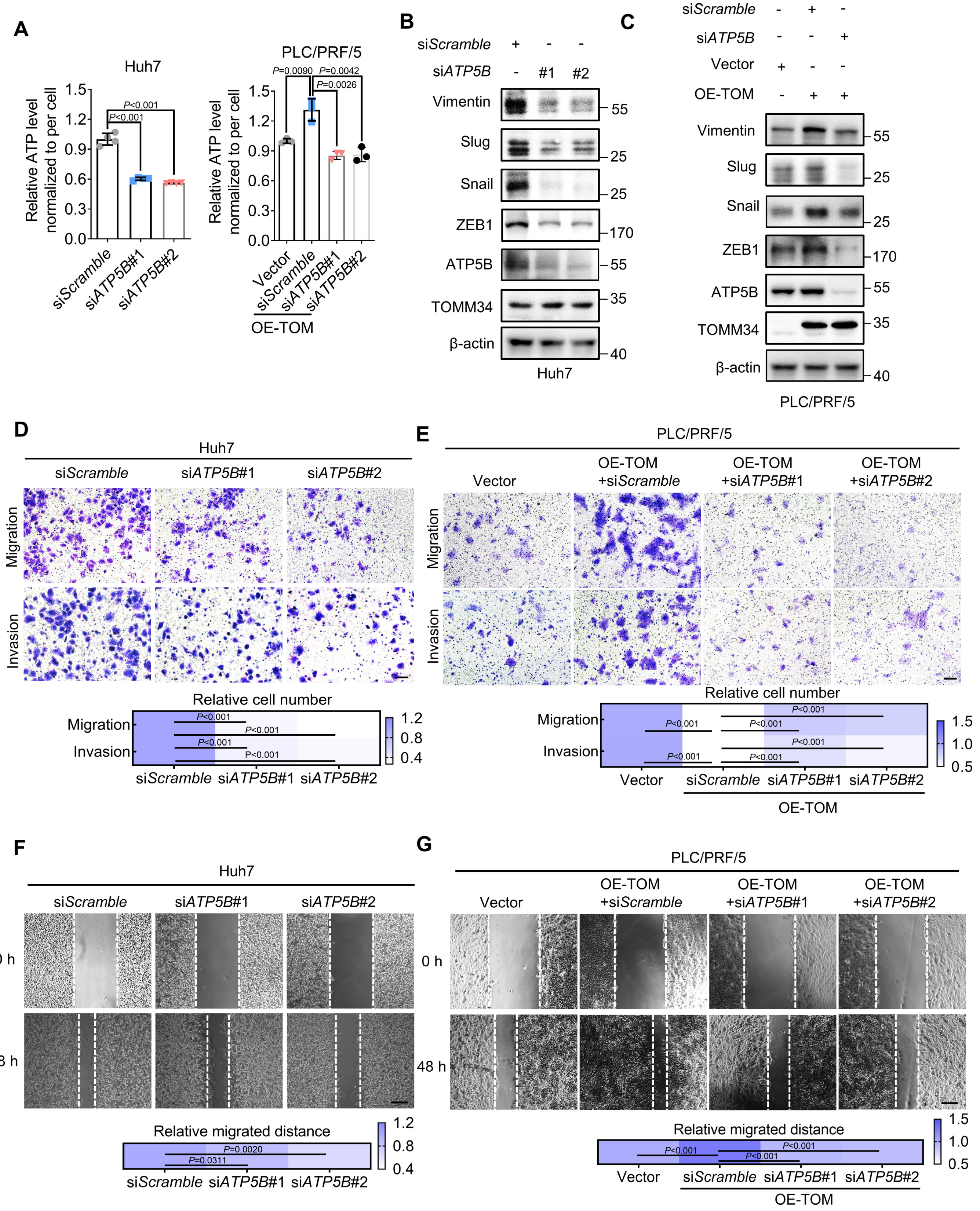
A
Huh7
Relative ATP level normalized to per cell
P<0.001
P<0.001
siScramble
siATP5B#1
siATP5B#2
PLC/PRF/5
P=0.0090
P=0.0042
P=0.0026
Vector
siScramble
siATP5B#1
siATP5B#2
OE-TOM
B
siScramble
siATP5B
Vimentin
Slug
Snail
ZEB1
ATP5B
TOMM34
β-actin
Huh7
C
Vector
OE-TOM
PLC/PRF/5
D
Migration
Invasion
Relative cell number
E
OE-TOM +siScramble
OE-TOM +siATP5B#1
OE-TOM +siATP5B#2
F
0 h
48 h
Relative migrated distance
P=0.0020
P=0.0311
G

Figure EV4.

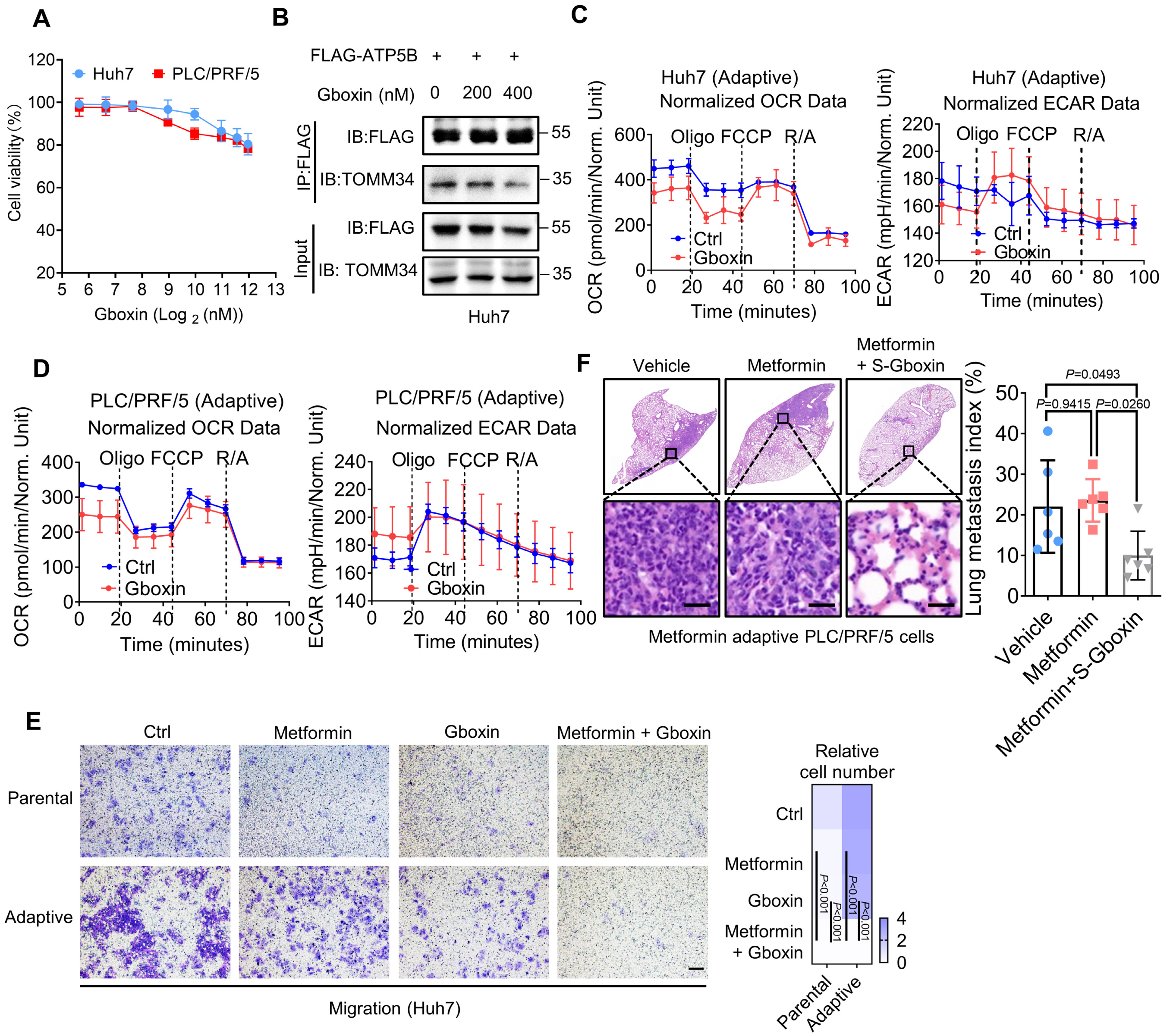

Figure EV5. Gboxin inhibits HCC metastasis by disturbing the interaction of TOMM34 and ATP5B.

A MTT assay showing the effects of Gboxin on the viability of HCC cells. (n = 6 biological replicates).

B Co-IP assay was performed to detect the interaction between TOMM34 and ATP5B treated with or without Gboxin for 24 h.

C, D The OCR and ECAR of indicated cells treated with or without Gboxin were measured using a Seahorse XF Analyzer (Oligo, oligomycin; FCCP, Carbonyl cyanide 4-(trifluoromethoxy)phenylhydrazone; R/A, rotenone/antimycin). (n = 3 biological replicates).

E Transwell assays showing the effect of combination treatment of Gboxin and metformin on the migration of parental and metformin-adaptive HCC cells (5×10^4 cells). Scale bars, 100 μm. (n = 3 biological replicates, Two-way ANOVA).

F A lung metastatic model showing migration of metformin-adaptive PLC/PRF/5 cells with or without Gboxin administration. Scale bars, 25 μm. (n = 6 mice for each group, Two-way ANOVA).

Data information: Data are presented as means ± SD.
Source data are available online for this figure.

 © 2022 The Authors
