# Supplementary material for: Disrupting metformin adaptation of liver cancer cells by targeting the TOMM34/ATP5B axis
Source: EMBO Mol Med. 2022 Nov 2;14(12):e16082. doi: 10.15252/emmm.202216082 (PMC9728056; doi:10.15252/emmm.202216082)
Supplement: Supplementary file 10 [file EMMM-14-e16082-s004.pdf]

EMBO Molecular Medicine

Disrupting metformin adaptation of liver cancer cells by targeting the TOMM34/ATP5B axis

Ping Jin, Jingwen Jiang, Li Zhou, Zhao Huang, Siyuan Qin, Hai-Ning Chen, Liyuan Peng, Zhe Zhang, Bowen Li, Maochao Luo, Tingting Zhang, Hui Ming, Ning Ding, Lei Li, Na Xie, Wei Gao, Wei Zhang, Edouard C. Nice, Yuquan Wei, Canhua Huang

DOI: 10.15252/emmm.202216082

Corresponding author: Canhua Huang (hcanhua@hotmail.com)

Review Timeline:

Submission Date:	27th Mar 22
Editorial Decision:	25th Apr 22
Revision Received:	24th Jul 22
Editorial Decision:	6th Sep 22
Revision Received:	23rd Sep 22
Accepted:	12th Oct 22

Editor: Lise Roth

Transaction Report:

(Note: With the exception of the correction of typographical or spelling errors that could be a source of ambiguity, letters and reports are not edited. Depending on transfer agreements, referee reports obtained elsewhere may or may not be included in this compilation. Referee reports are anonymous unless the Referee chooses to sign their reports.)

25th Apr 2022

Dear Dr. Huang,

Thank you for the submission of your manuscript to EMBO Molecular Medicine. We have now received feedback from the three reviewers who agreed to evaluate your manuscript. As you will see from the reports below, the referees acknowledge the interest of the study, but also raise a number of concerns that should be adequately addressed in revised version of this manuscript.

Addressing the reviewers' concerns in full will be necessary for further considering the manuscript in our journal, and acceptance of the manuscript will entail a second round of review. EMBO Molecular Medicine encourages a single round of revision only and therefore, acceptance or rejection of the manuscript will depend on the completeness of your responses included in the next, final version of the manuscript. For this reason, and to save you from any frustrations in the end, I would strongly advise against returning an incomplete revision.

Revised manuscripts should be submitted within three months of a request for revision; they will otherwise be treated as new submissions, except under exceptional circumstances in which a short extension is obtained from the editor.

When submitting your revised manuscript, please carefully review the instructions that follow below. We perform an initial quality control of all revised manuscripts before re-review; failure to include requested items will delay the evaluation of your revision.

We require:

1) A .docx formatted version of the manuscript text (including legends for main figures, EV figures and tables). Please make sure that the changes are highlighted to be clearly visible.

2) Individual production quality figure files as .eps, .tif, .jpg (one file per figure). For guidance, download the 'Figure Guide PDF' (https://www.embopress.org/page/journal/17574684/authorguide#figureformat).

3) A .docx formatted letter INCLUDING the reviewers' reports and your detailed point-by-point responses to their comments. As part of the EMBO Press transparent editorial process, the point-by-point response is part of the Review Process File (RPF), which will be published alongside your paper.

4) A complete author checklist, which you can download from our author guidelines (https://www.embopress.org/page/journal/17574684/authorguide#submissionofrevisions). Please insert information in the checklist that is also reflected in the manuscript. The completed author checklist will also be part of the RPF.
5) It is mandatory to include a 'Data Availability' section after the Materials and Methods. Before submitting your revision, primary datasets produced in this study need to be deposited in an appropriate public database, and the accession numbers and database listed under 'Data Availability'. Please remember to provide a reviewer password if the datasets are not yet public (see https://www.embopress.org/page/journal/17574684/authorguide#dataavailability).
In case you have no data that requires deposition in a public database, please state so in this section. Note that the Data Availability Section is restricted to new primary data that are part of this study.

5) For data quantification: please specify the name of the statistical test used to generate error bars and P values, the number (n) of independent experiments (specify technical or biological replicates) underlying each data point and the test used to calculate p-values in each figure legend. The figure legends should contain a basic description of n, P and the test applied. Graphs must include a description of the bars and the error bars (s.d., s.e.m.).

6) We would also encourage you to include the source data for figure panels that show essential data. Numerical data should be provided as individual .xls or .csv files (including a tab describing the data). For blots or microscopy, uncropped images should be submitted (using a zip archive if multiple images need to be supplied for one panel). Additional information on source data and instruction on how to label the files are available at
.

7) Our journal encourages inclusion of *data citations in the reference list* to directly cite datasets that were re-used and obtained from public databases. Data citations in the article text are distinct from normal bibliographical citations and should directly link to the database records from which the data can be accessed. In the main text, data citations are formatted as follows: "Data ref: Smith et al, 2001" or "Data ref: NCBI Sequence Read Archive PRJNA342805, 2017". In the Reference list, data citations must be labeled with "[DATASET]". A data reference must provide the database name, accession number/identifiers and a resolvable link to the landing page from which the data can be accessed at the end of the reference. Further instructions are available at .

8) We replaced Supplementary Information with Expanded View (EV) Figures and Tables that are collapsible/expandable online. A maximum of 5 EV Figures can be typeset. EV Figures should be cited as 'Figure EV1, Figure EV2" etc... in the text and their respective legends should be included in the main text after the legends of regular figures.
- For the figures that you do NOT wish to display as Expanded View figures, they should be bundled together with their legends in a single PDF file called *Appendix*, which should start with a short Table of Content. Appendix figures should be referred to in the main text as: "Appendix Figure S1, Appendix Figure S2" etc.
- Additional Tables/Datasets should be labeled and referred to as Table EV1, Dataset EV1, etc. Legends have to be provided in a separate tab in case of .xls files. Alternatively, the legend can be supplied as a separate text file (README) and zipped together with the Table/Dataset file.
See detailed instructions here:
.

9) The paper explained: EMBO Molecular Medicine articles are accompanied by a summary of the articles to emphasize the major findings in the paper and their medical implications for the non-specialist reader. Please provide a draft summary of your article highlighting
- the medical issue you are addressing,
- the results obtained and
- their clinical impact.
This may be edited to ensure that readers understand the significance and context of the research. Please refer to any of our published articles for an example.

10) For more information: There is space at the end of each article to list relevant web links for further consultation by our readers. Could you identify some relevant ones and provide such information as well? Some examples are patient associations, relevant databases, OMIM/proteins/genes links, author's websites, etc...

11) Author contributions: the contribution of every author must be detailed in a separate section (before the acknowledgments).

12) Conflict of interest: We updated our journal's competing interests policy in January 2022 and request authors to consider both actual and perceived competing interests. Please review the policy https://www.embopress.org/competing-interests and update your competing interests if necessary.

13) Every published paper now includes a 'Synopsis' to further enhance discoverability. Synopses are displayed on the journal webpage and are freely accessible to all readers. They include a short stand first (maximum of 300 characters, including space) as well as 2-5 one-sentences bullet points that summarizes the paper. Please write the bullet points to summarize the key NEW findings. They should be designed to be complementary to the abstract - i.e. not repeat the same text. We encourage inclusion of key acronyms and quantitative information (maximum of 30 words / bullet point). Please use the passive voice. Please attach these in a separate file or send them by email, we will incorporate them accordingly.

Please also suggest a striking image or visual abstract to illustrate your article as a PNG file 550 px wide x 300-600 px high.

14) As part of the EMBO Publications transparent editorial process initiative (see our Editorial at http://embomolmed.embopress.org/content/2/9/329), EMBO Molecular Medicine will publish online a Review Process File (RPF) to accompany accepted manuscripts.
In the event of acceptance, this file will be published in conjunction with your paper and will include the anonymous referee reports, your point-by-point response and all pertinent correspondence relating to the manuscript. Let us know whether you agree with the publication of the RPF and as here, if you want to remove or not any figures from it prior to publication.
Please note that the Authors checklist will be published at the end of the RPF.

EMBO Molecular Medicine has a "scooping protection" policy, whereby similar findings that are published by others during review or revision are not a criterion for rejection. Should you decide to submit a revised version, I do ask that you get in touch after three months if you have not completed it, to update us on the status.

I look forward to receiving your revised manuscript.

Yours sincerely,

Lise Roth

Lise Roth, PhD
Editor

***** Reviewer's comments *****

Referee #1 (Comments on Novelty/Model System for Author):

This study shows that metformin has different impact on primary tumor growth and metastasis. This is an important observation that may have significant clinical impact given the numerous clinical trials on metformin. The study uses different and complementary experimental approaches from in vitro work to in vivo work and validation in clinical samples. This represents a clear strength of this study.

However, the presentation of the paper should be modified and re-contextualized. The interpretation of several results needs to be re-examined.

Referee #1 (Remarks for Author):

The paper by Jin and colleagues examined the role of metformin in hepatocellular carcinoma progression. They report that metformin is linked to EMT and metastasis, in part through regulation of ATP5B and TOMM34. Specifically, the authors claim that TOMM34 interacts with ATP5B to stabilize it and promote OXPHOS. The study encompasses a broad range of complementary experimental approaches including in vitro and in vivo work as well as validation of results with clinical samples. Overall, the results presented bring important new knowledge. However, the narrative of the paper should be revised and the interpretation of several results needs to be revisited.

Major comments:

1) The paper is presented as if the lack of significant clinical results with metformin is due to acquisition of resistance. There is little data to support that claim. Most clinical trials with metformin did not lead to improved clinical outcomes. Several studies put forward explanations for this, including potential problems with drug concentration and drug distribution. In my opinion, the most significant discovery of this study is the differential effect of metformin on primary tumor growth and metastasis. The authors show that metformin decreases primary tumor growth and promotes metastasis. Given that metformin targets mitochondrial complex I, this result indicates that the metabolic requirements for primary tumor growth and metastasis are different. This is very important and should be emphasized.
2) The authors report that long term exposure to metformin leads to increased OXPHOS. It is stated at several places that OXPHOS is increased based on elevated levels of the expression of genes or proteins involved in OXPHOS. Elevated expression of mitochondrial genes or proteins does not necessarily mean increased OXPHOS, and this should be clarified. Importantly, long term exposure to metformin can lead to elevated expression of mitochondrial genes and transcripts. However, metformin blocks complex I and it is unlikely that respiration would be increased in the presence of metformin even in the presence of elevated mitochondrial proteins. The respiration assays should be repeated in the presence of metformin to assess this claim. It is certainly interesting that cells exposed to metformin increase the expression of mitochondrial genes and proteins and that upon metformin removal, these cells display increased OXPHOS. This indicates that the cells attempt to restore mitochondrial ATP production upon its inhibition by metformin.
3) The authors put forward the notion that TOMM34 stabilizes ATP5B. It is clear from the data that these two proteins interact. However, there is no evidence that the impact of TOMM34 on ATP5B is through stabilization of the protein. Several other explanations should be explored, including the possibility that TOMM34 facilitates the import of this ATP synthase subunit in the mitochondria.
4) The rationale for using the different cell lines should be provided. Notably, one cell line is used for knockdown experiments while another one is used for overexpression.

Other comments:
1) To determine glycolytic activity, intracellular lactate was measured. Extracellular lactate should be quantified. Numerous studies have reported that metformin exposure leads to elevated glycolysis and dependency on glucose for survival and growth.
2) The impact of TOMM34 on mitochondrial OXPHOS and glycolysis should be determined in the absence of metformin.
3) In figure 5, extracellular ATP was shown to impact EMT markers and metastatic potential. However, cells do not take up ATP. The authors should provide an explanation for these results.
4) The impact of gboxin on mitochondrial OXPHOS and glycolysis needs to be determined.
5) What is the impact of the combination of metformin and gboxin on non-transformed cells?
6) Fig.3F: ECAR data should be presented.
7) Fig.3G: Different exposures are needed for the different subunits.

8) Fig.S1F and G: the differences in cell viability between the cell lines are striking-this should be discussed. Also, different concentrations of drugs or glucose should be tested as for certain conditions, the viability is lower than 50% at baseline.

Referee #2 (Remarks for Author):

The anti-tumor effects of metformin has been widely reported in recent years, however, drug resistance and tumor metastasis are also emerging during metformin treatment. In this manuscript, the authors observed the adaptation of HCC cells in long-term metformin exposure, which was characterized by an epithelial-mesenchymal transition (EMT) and tumor metastasis. Mechanically, the authors found that TOMM34 was up-regulated under metformin-induced metabolic stress and interacted with ATP5B to preserve ATPase activity, which then promoted OXPHOS and ATP production. As a result, the metabolic switch provided HCC cells the metastatic potential. More significantly, the authors elaborated that Gboxin, an OXPHOS inhibitor, could disturb the interaction of TOMM34 and ATP5B, thereby suppressing tumor survival and metastasis during metformin adaption, which may hold great clinical potential for HCC therapy. Overall, the findings are novel and interesting, and the experiments in general are clearly described and presented with conclusive data. Meanwhile, I have multiple concerns that the authors may want to address before publication.

1. The highlight of this study is that TOMM34/ATP5B-ATP synthesis axis plays an essential role in HCC metformin adaptation. However, a big part of experiments was performed in parental HCC cell lines, like in Figure.5A-5F/5H and Figure.6A/B. It's recommended to repeat some of the key experiments in the adaptive HCC cell lines used in this study;
2. For the functional experiments of this study, the authors only manipulated the TOMM34 knockdown in Huh7 cells and overexpression in PLC/PRF/5 cells to validate the pivotal role of TOMM34 during metformin adaptation. To further confirm the conclusion, the results of TOMM34 overexpression in Huh7 and knockdown in PLC/PRF/5 cells should be supplied;
3. By integrating the MS results and the MitoCarta3.0 database, the authors identified the TOMM34 as the key factor mediating the metformin adaptation. However, the heatmap in Fig.2B shows that it is TOMM34 that is greatly upregulated in the adaptive Huh7 cells, while in the adaptive PLC/PRF/5 cells is the ODPR. The authors may want to explain why they focus only on TOMM34;
4. In Fig.3G, the WB results didn't show the change of ATP5A1 and UQCRC2 expression upon the knockdown or over-expression of TOMM34. The authors may want to repeat and optimize the assays;
5. As authors described the over-expression of TOMM34 could up-regulate the expression of ATP5B in Fig.4E, however, the over-expression of Myc-TOMM34 seems to down-regulate the expression of Flag-ATP5B in Figure.6B. The authors may want to repeat and optimize the assays.

Referee #3 (Comments on Novelty/Model System for Author):

Need to provide details on the characterization of the presumptive HCC models utilized.

Referee #3 (Remarks for Author):

The authors set to characterize the impact of metabolic adaptation to metformin treatment in HCC-derived patient-derived xenografts and cell lines. They showed that treatment of a subQ HCC PDX model with metformin resulted in a slight suppression of growth and an enhanced dissemination of these cells to the lung and liver. Using two HCC-derived cell lines exposed in vitro to increasing metformin concentrations, they selected populations that were able to grow in the presence of 5 mM metformin and showed a more "mesenchymal" phenotype.These cells also seemed to have higher ATP level compared to the parent population, while displaying an overall reduction of mitochondrial complex I activity, when a concomitant increase in complex V activity. These cells did not seem to compensate suppression of OXPHOS with enhanced glycolytic activity, as evidenced by lack of lactate accumulation. When comparing treated and parental cells by untargeted mass spectrometry, the authors identified TOMM34, a mitochondrial outer membrane protein as elevated in the treated cells, and verified its accumulation by immunoblot analysis. Accumulation of TOMM34 seemed to represent a functional adaptation to metformin treatment, as suppressing its accumulation by shRNA knockdown, affected survival of treated but not control cells. TOMM34 also seemed to protect cells from metabolic stress induced by 2-DG, as it might expected for a mitochondrial regulator , upon suppression of glycolysis.
The authors then tested whether TOMM34 accumulation might play a role in the dissemination phenotype consequent to metformin treatment and showed that in an orthotpic (liver) model, TOMM34 levels were elevated in lung lesions compared to the liver ones. TOMM34 suppression also seemed to induce an "epithelial-like" phenotype in another HCC cell line, and inhibited its dissemination to the lungs upon tail vein injection. TOMM34 overexpression seemed to enhance lung dissemination. The authors also provide evidence of mesenchymal features and enhanced ability to migrate in TOMM34 overexpressing cells.
Upon RNA sequencing, the authors show that TOMM34 knockdown, compared to parental,cells, have significant alterations in OXPHOS pathway genes as also evidence by the presence of mitochondrial alteration by TEM. mtDNA, cellular ATP levels and OCR were all reduced upon TOMM34 suppression, as well as expression of mitochondrial OXPHOS complexes subunits, impacting complex I and V activity.
By IP-mass spec analysis the authors identified ATP5B as a TOMM34-interacting protein, with further evidence of interaction in HEK293 cells by co-IP of tagged proteins and evidence of interaction of the endogenous proteins in the Huh67 cells lines.

Changes in TOMM34 levels by overexpression or knockdown seemed to concomitantly affect ATP5B levels.
The authors also show that TOMM34 and ATP5B co-colocalized in the invasive front area of cells in clinical samples and that both proteins were highly expressed in metastatic tissues. From the analysis of deletion mutants of TOMM34, they identify a 34-aa region responsible for its interaction with ATP5B. They then opted to determine whether suppression of ATP5B expression affected the ability of TOMM34 overexpression to sustain ATP accumulation and EMT. They also show that exogenous ATP supplementation restored EMT markers and dissemination ability upon TOMM34 knockdown. ATP5B silencing also inhibited migration and invasion capacity in vitro pf TOMM34 overexpressing cells as well that of metformin adapted cells. They also provide evidence that ATP5B expression correlates with worse prognosis upon analysis of available HCC pathology specimens. They then evaluate whether a published covalent binder OXPHOS inhibitor, Gboxin, described as interacting with ATP5B, affected the interaction of TOMM34 with ATP5B and this was indeed the case. At concentrations that did not impact cell growth, Gboxin seemed to affect EMT marker expression and cell migration, while also impacting ATP accumulation upon TOMM4 overexpression.
Lastly, the authors provide evidence that Gboxin and metformin could be combined leading to a synergistic effect on tumor growth and dissemination behavior, as also evidenced by a reduction of circulating tumor cell load in the PDX model.
The manuscript utilizes multiple experimental approaches to substantiate findings in support the authors claim that long-term effects of metformin treatment on HCC cells induce both an enhanced dissemination ability and deregulation of mitochondrial function via upregulation of certain mitochondrial proteins, which compensates for the effects of the drug on oxidative phosphorylation. While intriguing, I believe that much more work would be needed to solidify these findings, whether this applies specifically to hepatocellular carcinoma, whether the models utilize recapitulate the genetics and cell of origin of the diseases, whether the observed effect are specific to the drug being tested, a recognized polypharmacy entity, what the mechanism are of enhanced migration and dissemination. I would perhaps refocus the manuscript around a defined set of key findings. Breadth of scope should not be an alternative to depth of scope.
1. Better characterization of cell and PDX models under use should be provided. Antibody validation should be provided in the context of knockdown for immunochemistry/immunofluorescence.
2. Is the described "adaptative" mechanism via the TOMM34/ATP5B axis specifically related to complex I inhibition? The authors may want to test other complex I inhibitors, such as phenformin or others, in TOMM34 or ATP5B deficient adaptive HCC cells.
3. Conversely, would this mechanism apply to other mitochondrial inhibitors, e.g. antimycin (Complex III) or oligomycin (complex V)?
4. In cells exposed to long term treatment with metformin, you may need to evaluate any changes in the activity of other mitochondrial complexes, not just Complex I and V (Fig1J and K).
5. Are there any changes in OCR and ECAR upon short-term (24hr) vs long-term treatment? Are TOMM34 protein levels or its interaction with ATP5B increased upon short term treatment (24hr)?
6. Metformin targets multiple signaling pathways including AMPK, mTOR, IGF signaling, and mitochondrial complex I. In your evaluation of MS data with the MitoCarta 3.0 database, which is specific for mitochondrial related genes, might you have missed on an opportunity to look more broadly at adaptive mechanisms (Fig2A-B)? How should we think about other non-mitochondrial mediated effects of metformin treatment in this context?
7. Are there any other metabolic changes in cell exposed to long term metformin treatment versus parental HCC cells? And does TOMM34 overexpression in these cells induce similar changes?
8. In Fig3G, mitochondrial CII and CIII level, rather than CV, were significantly regulated by TOMM34 KD or OE. How should this be interpreted? Mitochondrial biosynthesis related? Are CII and CIII activities increased? Why there is no CI band?
9. In the heatmap of Fig4E, OE-TOM group showed decreased TOMM34, inconsistent with western blotting data.
10. TOMM34 is an outer mitochondrial membrane translocase, while ATP5B is a subunit of ATP synthase which is located in the inner membrane and faces the mitochondrial matrix. How could they interact with each other so tightly (Fig4C)?
11. To prove the relevance of TOMM34/ATP5B interaction, you may need to utilize the TPR4 region-truncated protein to perform migration and invasion rescue experiments. (FigS4)
12. Would migration or invasion be rescued by ATP5B expression in TOMM34 knockdown HCC cells?
13. In Fig6I, a Gboxin alone group should be added to prove the advantage of combination effects in tumor suppression.
14. Gboxin was found to inhibit OXPHOS inhibitor by inhibition of the F0F1 ATP synthase in GBM. This covalent interactor also binds other components of mitochondrial complexes (Nature volume 567, pages341-346 (2019)). In the context of that manuscript there seem to be no evidence of interaction with TOMM34/ATP5B. Is it possible therefore that Gboxin directly inhibits ATP production rather than disturbing interaction of TOMM34 and ATP5B? Please verify it.

Point-by-point response for EMM-2022-16082

The revised sections of the manuscript are marked in blue.

***** Reviewer's comments *****

Referee #1 (Comments on Novelty/Model System for Author):

This study shows that metformin has different impact on primary tumor growth and metastasis. This is an important observation that may have significant clinical impact given the numerous clinical trials on metformin. The study uses different and complementary experimental approaches from in vitro work to in vivo work and validation in clinical samples. This represents a clear strength of this study.

However, the presentation of the paper should be modified and re-contextualized. The interpretation of several results needs to be re-examined.
Re: Thank you very much for your valuable and positive comments. We have performed complementary experiments with appropriate data interpretation. We also carefully re-contextualized several presentations in the revised manuscript. In addition, the interpretation of several results was carefully re-examined. The attached Point-by-point response will provide the description of changes made according to your suggestions.

Referee #1 (Remarks for Author):

The paper by Jin and colleagues examined the role of metformin in hepatocellular carcinoma progression. They report that metformin is linked to EMT and metastasis, in part through regulation of ATP5B and TOMM34. Specifically, the authors claim that TOMM34 interacts with ATP5B to stabilize it and promote OXPHOS. The study

encompasses a broad range of complementary experimental approaches including in vitro and in vivo work as well as validation of results with clinical samples. Overall, the results presented bring important new knowledge. However, the narrative of the paper should be revised and the interpretation of several results needs to be revisited.

Major comments:

1) The paper is presented as if the lack of significant clinical results with metformin is due to acquisition of resistance. There is little data to support that claim. Most clinical trials with metformin did not lead to improved clinical outcomes. Several studies put forward explanations for this, including potential problems with drug concentration and drug distribution. In my opinion, the most significant discovery of this study is the differential effect of metformin on primary tumor growth and metastasis. The authors show that metformin decreases primary tumor growth and promotes metastasis. Given that metformin targets mitochondrial complex I, this result indicates that the metabolic requirements for primary tumor growth and metastasis are different. This is very important and should be emphasized.

Re: Thank you for your valuable comments. Compelling evidence indicates that cancer cells can divert metabolites into anabolic pathways to support their rapid proliferation, while migratory/invasive cancer cells specifically require mitochondrial respiration and increased ATP production (LeBleu *et al*, 2014; Lehuédé *et al*, 2016). In other words, it is possible that tumor metastasis may be more dependent on OXPHOS rather than glycolysis. During adaptation to metabolic stress, HCC cells may undergo metabolic shift from glycolysis to OXPHOS, thus restricting tumor growth but promoting metastasis. In line with this view, we observed that long-term treatment of metformin conferred a relatively high respiration rate on HCC cells (**Fig. 1I**). Moreover, these adaptive cells exhibited decreased proliferative ability but enhanced metastatic potential (**Fig. EV1A** and **Fig. 1H**), indicating that adaptive HCC cells might rely on enhanced OXPHOS for metastasis. Using a OXPHOS inhibitor, Gboxin, could inhibit the migration and invasion of both parental and metformin-

adaptive HCC cells (**Fig. EV5E**). In addition, inhibition of glycolysis with 2-DG at a dosage that could dramatically decrease cell growth (**Response Fig. 1A-B**) had no obvious effect on the metastasis of metformin adaptive cells (**Response Fig. 1C-D**), suggesting the invasion/migration of cells may rely more on OXPHOS than glycolysis. Together, our results indicate that the metabolic requirements for primary tumor growth and metastasis might be different. We have added the descriptions of Fig. EV1A (**line 173**) and Fig. 1I (**line 182**) in the revised manuscript.

Fig. 1

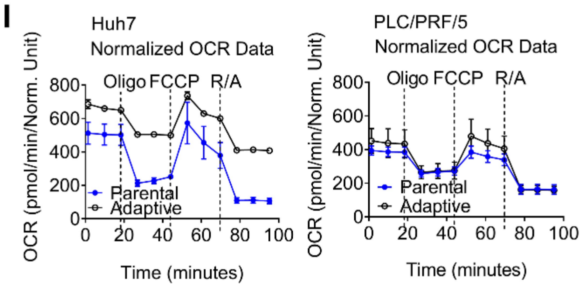

Fig. 1. (I) Oxygen consumption rate (OCR) in parental Huh7, adaptive Huh7, parental PLC/PRF/5 and adaptive PLC/PRF/5 cells was measured using a Seahorse XF24 Extracellular Analyzer.

Fig. EV1

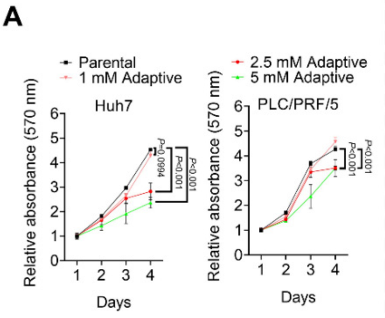

Fig. 1

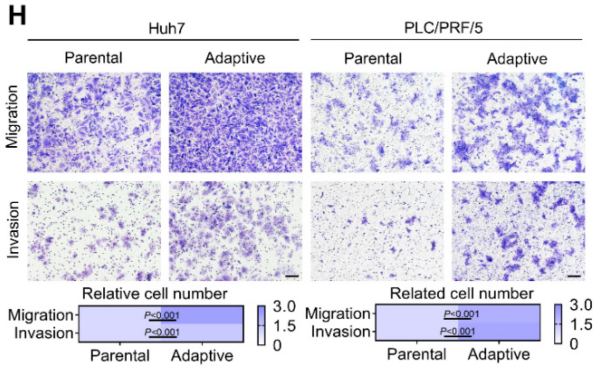

Fig. EV1. (A) MTT assays showing the growth of parental HCC cells and metformin-adaptive HCC cells (obtained from long-term exposure to different concentrations of metformin)

Fig. 1. (H) Migration and invasion of parental and metformin adaptive HCC cells were evaluated by Transwell assays. Scale bars, 100 μm.

Fig. EV5

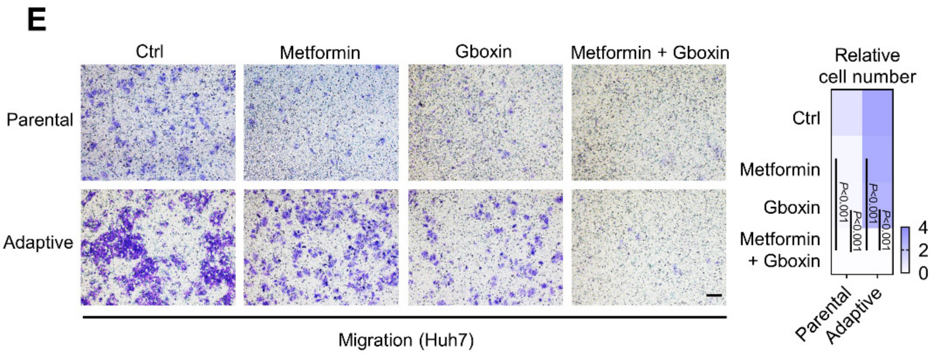

Fig. EV5. (E) Transwell assays showing the effect of combination treatment of Gboxin and metformin on the migration of parental and metformin adaptive Huh7 cells. Scale bars, 100 μm.

Response Fig. 1

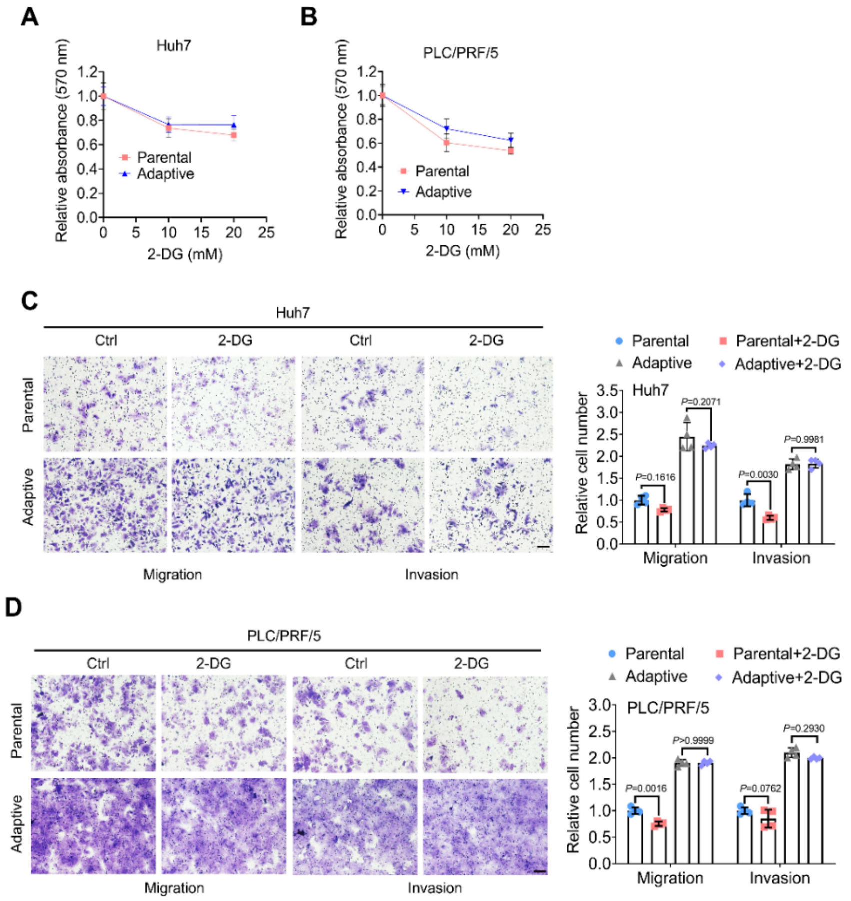

Response Fig. 1. (A-B) MTT assays showing the proliferation of cells treated with indicated concentration of 2-DG for 24 hours. (C-D) Transwell assays showing the migration and invasion of indicated cells treated with or without 10 mM 2-DG for 24 hours. Scale bars, 100 μm.

2) The authors report that long term exposure to metformin leads to increased OXPHOS. It is stated at several places that OXPHOS is increased based on elevated levels of the expression of genes or proteins involved in OXPHOS. Elevated expression of mitochondrial genes or proteins does not necessarily mean increased OXPHOS, and this should be clarified. Importantly, long term exposure to metformin can lead to elevated expression of mitochondrial genes and transcripts. However, metformin blocks complex I and it is unlikely that respiration would be increased in the presence of metformin even in the presence of elevated mitochondrial proteins. The respiration assays should be repeated in the presence of metformin to assess this claim. It is certainly interesting that cells exposed to metformin increase the expression of mitochondrial genes and proteins and that upon metformin removal, these cells display increased OXPHOS. This indicates that the cells attempt to restore mitochondrial ATP production upon its inhibition by metformin.

Re: Thank you for your valuable comments. We have repeated respiration assays in the presence of metformin according to your kind suggestion. As shown in **Response Fig. 2A-B**, although the OCR of adaptive cells rebounded above parental cells, respiration was inhibited in the presence of metformin. We therefore redefined the conception of "Adaptive cells" — "It is worthwhile noting that, although the proliferation rate of these residual HCC cells was decreased (Fig. EV1A), they displayed an obvious EMT and polarized phenotype (Fig. 1F-G) and obtained enhanced migratory and invasive capacity (Fig. 1H) after metformin withdrawal. We therefore defined this phenotype as metformin adaptation, and these residual cells were named as metformin adaptive HCC cells" in the revised manuscript (**Line 171-176**) to avoid ambiguous interpretation. Furthermore, the mRNA levels of mitochondrial genes of cells exposed to metformin were increased, and upon

metformin removal, the expression of these mitochondrial genes also increased. These data suggest that tumor cells may attempt to restore mitochondrial respiration and ATP production upon the exposure to metformin by upregulating the expression of mitochondrial genes (**Response Fig. 2C)**.

Response Fig. 2

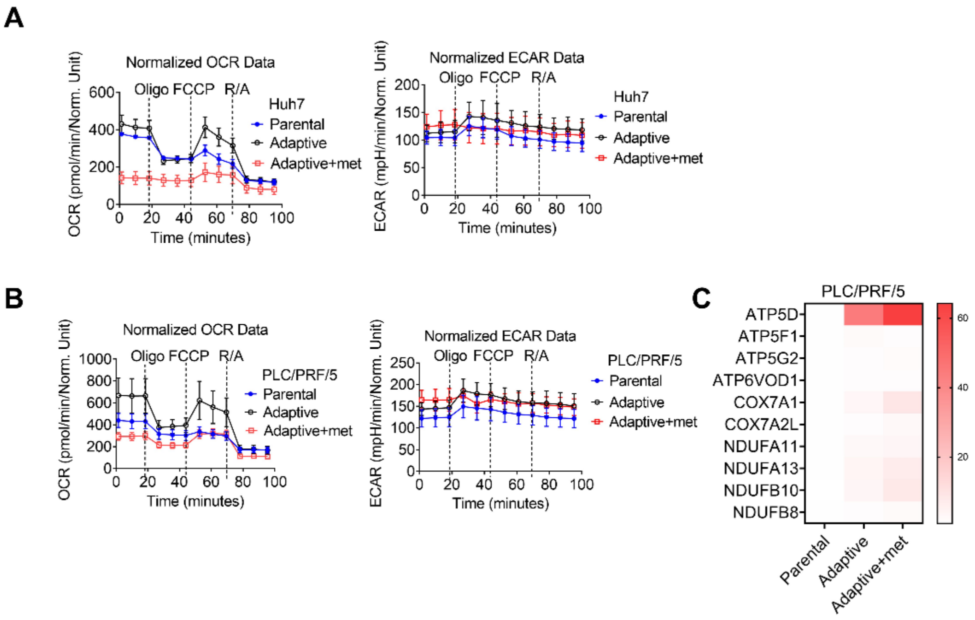

Response Fig. 2. (A-B) Oxygen consumption rate (OCR) and Extracellular Acidification Rate (ECAR) in Huh7 and PLC/PRF/5 cells with or without TOMM34 knockdown were measured using a Seahorse XF24 Extracellular Analyzer. (C) The mRNA levels of indicated OXPHOS genes in parental and adaptive cells with or without the removal of metformin.

3) The authors put forward the notion that TOMM34 stabilizes ATP5B. It is clear from the data that these two proteins interact. However, there is no evidence that the impact of TOMM34 on ATP5B is through stabilization of the protein. Several other explanations should be explored, including the possibility that TOMM34 facilitates the import of this ATP synthase subunit in the mitochondria.

Re: Thank you for your valuable comments. We firstly measured the influence of TOMM34 on the stability of ATP5B protein. As shown in **Fig. 4F-G**, knockdown of

TOMM34 accelerated the turnover of ATP5B protein, while overexpression of TOMM34 prolonged the half-life of ATP5B protein, indicating that TOMM34 may regulate the stability of ATP5B protein. We have added the descriptions of **Fig. 4F-G** in the revised manuscript (**line 298-304**). According to your kind suggestion, we also detected the impact of TOMM34 on the subcellular translocation of ATP5B. As shown in **Response Fig. 3**, knockdown of TOMM34 reduced ATP5B protein level in the mitochondrial fraction, while overexpression of TOMM34 increased the ATP5B protein level located in mitochondria, suggesting that TOMM34 might also be involved in the subcellular translocation of ATP5B. But whether the alteration of ATP5B level in mitochondrial fraction attributes to protein stabilization needs further investigation.

Fig. 4

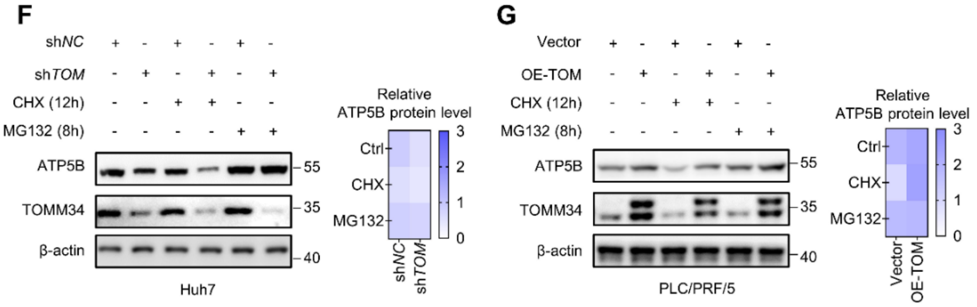

Fig. 4. (F-G) Western blot showing protein levels of ATP5B in Huh7 (F) or PLC/PRF/5 (G) cells treated with or without 10 mg/mL cyclohexane (CHX) or 25 μM MG132 for indicated period.

Response Fig. 3

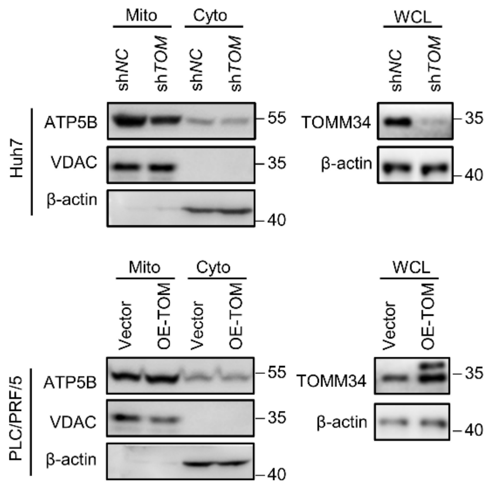

Response Fig. 3. Western blot showing the protein level of ATP5B in mitochondrial and cytoplasmic fraction of cells with different TOMM34 expression. VDAC and actin were used as mitochondrial and cytoplasm marker, respectively.

4) The rationale for using the different cell lines should be provided. Notably, one cell line is used for knockdown experiments while another one is used for overexpression.

Re: Thank you for your valuable comments. We chose PLC/PRF/5 and Huh7 cell lines for overexpression or knockdown respectively according to the basal level of TOMM34 protein in these HCC cell lines. We found that Huh7 cells expressed high TOMM34 protein level while the protein level of TOMM34 in PLC/PRF/5 cells was relatively low (**Response Fig. 4**). Therefore, Huh7 was used for TOMM34 knockdown experiments while PLC/PRF/5 was used for TOMM34 overexpression.

Response Fig. 4

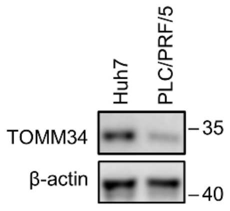

Response Fig. 4. Western blot showing the basal level of TOMM34 protein in Huh7

and PLC/PRF/5 cells**.**

Other comments:

1) To determine glycolytic activity, intracellular lactate was measured. Extracellular lactate should be quantified. Numerous studies have reported that metformin exposure leads to elevated glycolysis and dependency on glucose for survival and growth.

Re: Thank you for your valuable suggestion. We quantified extracellular lactate in the medium of parental or metformin-adaptive cells. The results indicated that the lactate was not obviously increased upon long-term metformin exposure (**Response Fig. 5A**). We also evaluated the impact of lactate in short-term metformin-treated cells and found that combinational use of sodium dichloroacetate (DCA, a lactate inhibitor) exacerbated the inhibitory efficacy of metformin on cancer cell growth (**Response Fig. 5B**), suggesting that short-term metformin exposure did lead to the dependency on glucose for survival and growth**.**

Response Fig. 5

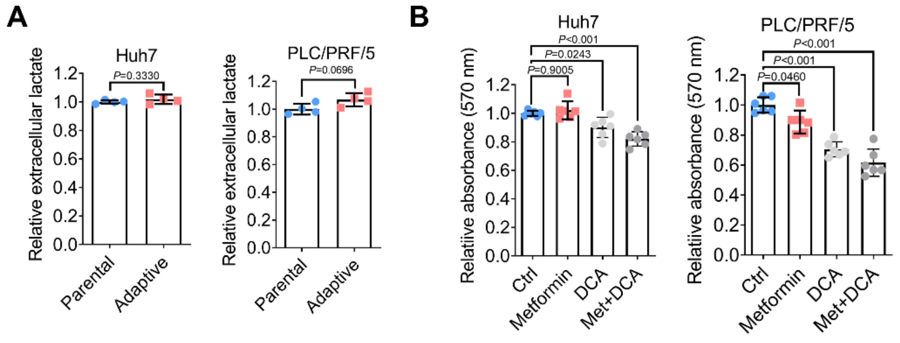

Response Fig. 5. (A) Lactate level in indicated cell supernatants was measured by lactate detection kit. (B) MTT assays showing the proliferation of indicated cells treated with indicated drugs for 24 hours.

2) The impact of TOMM34 on mitochondrial OXPHOS and glycolysis should be determined in the absence of metformin.

Re: Thank you for your valuable comment. The impact of TOMM34 on OCR and extracellular acidification rate (ECAR) in the absence of metformin has been already included in **Fig. 3F**. These results indicate that TOMM34 deficiency impaired mitochondrial OXPHOS but had little effect on glycolysis of HCC cells.

Fig. 3

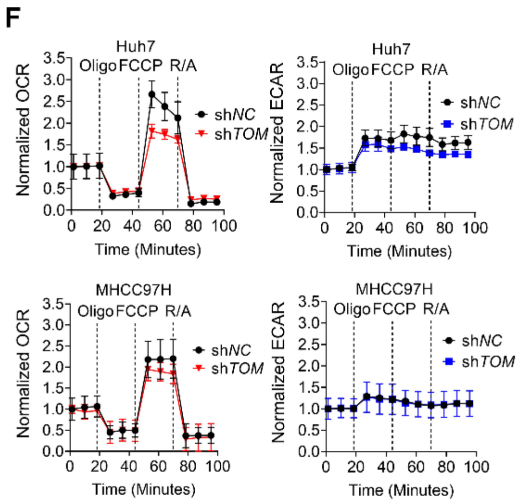

Fig. 3. (F) OCR and Extracellular Acidification Rate (ECAR) in Huh7 and HMCC97H cells with or without TOMM34 knockdown were measured using a Seahorse XF24 Extracellular Analyzer.

3) In figure 5, extracellular ATP was shown to impact EMT markers and metastatic potential. However, cells do not take up ATP. The authors should provide an explanation for these results.

Re: Thank you for your critical comments. Recent studies suggested that although cells do not take up ATP, ATP can regulate cell behavior (Sharma *et al*, 2020). For example, ATP regulates cell behaviors via interacting with P2 purinoceptors (including P2X and P2Y receptors) on cell membrane (review in our paper: From purines to purinergic signaling: molecular functions and human diseases. ***Signal Transduction and Targeted Therapy***) (Huang *et al*, 2021). Therefore, ATP may modulate cellular events even though cells do not take up it. However, here we intended to demonstrate the effect of ATP, as a cellular energy source, on cell migration and invasion. We have now

deleted this figure in the revised manuscript to avoid misunderstanding. Instead, we overexpressed exogenous ATP5B in TOMM34 knockdown metformin-adaptive HCC cells, and found that the expression of mesenchymal markers, the cellular ATP level, cell migration and invasion were significantly restored (**Fig. 5F-H**). We have added the descriptions of **Fig. 5F-H** in the revised manuscript (**Line 335-338**).

Fig. 5

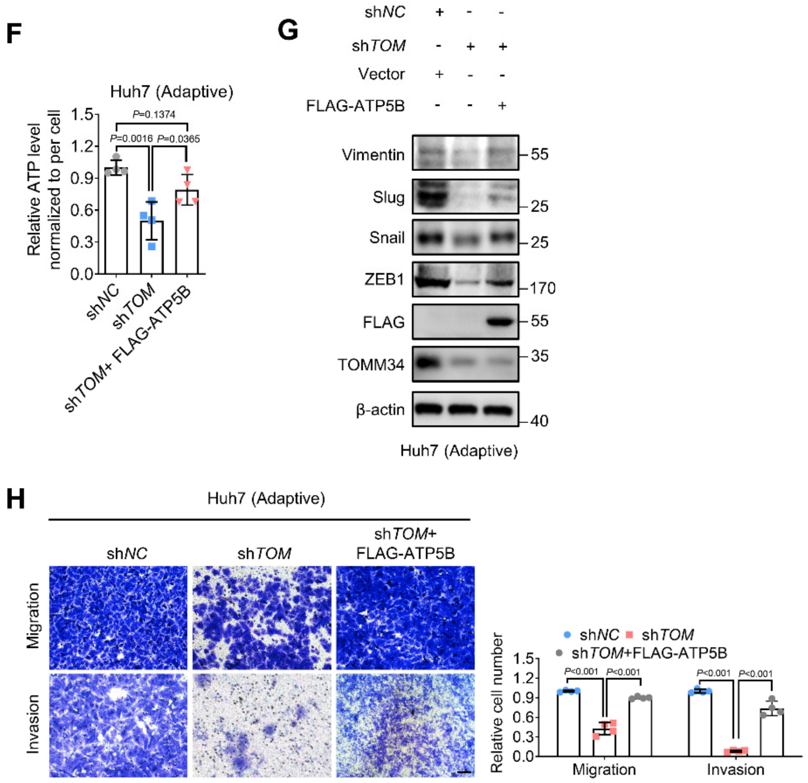

Fig. 5. (F) ATP level of indicated cells was measured by ATP detection kit. (G) Western blot showing the expression of EMT markers in cells with or without knockdown of TOMM34 or overexpression of ATP5B. (H) Transwell assays showing the migration and invasion of indicated cells. Scale bar, 100 μm.

4) The impact of gboxin on mitochondrial OXPHOS and glycolysis needs to be determined.

Re: Thank you for your valuable comment. According to your kind suggestion, we

measured the OCR and ECAR in adaptive cells treated with or without Gboxin for 24 h. As shown in **Fig. EV5C-D**, Gboxin decreased OXPHOS while having no obvious effect on glycolysis. We have added the relevant descriptions in the revised manuscript (**Line 363-365**).

Fig. EV5

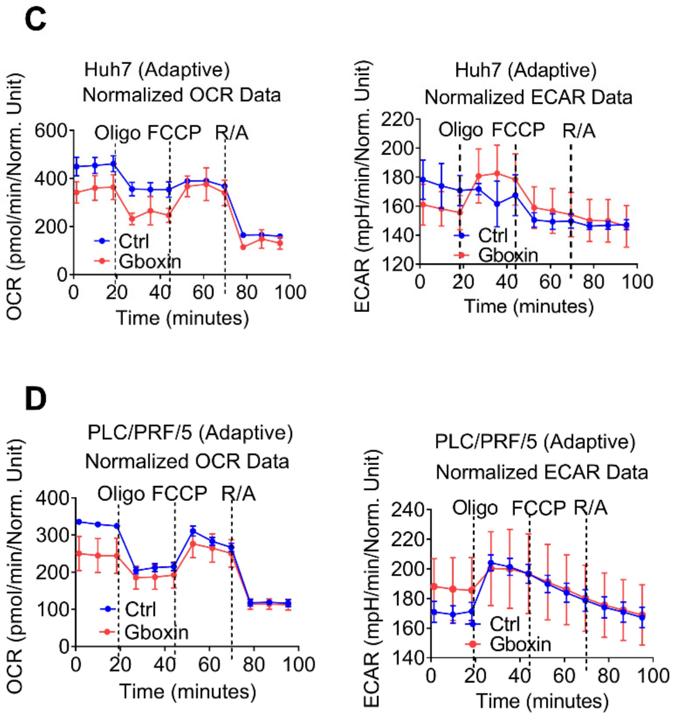

Fig. EV5. (C-D) OCR and ECAR of indicated cells treated with or without Gboxin were measured using a Seahorse XF24 Extracellular Analyzer.

5) What is the impact of the combination of metformin and gboxin on non-transformed cells?

Re: According to your kind suggestion, we determined the proliferation level, migration, and invasion ability of non-transformed liver cells LO2 upon combinational treatment with metformin and Gboxin. As shown in **Appendix Fig. S5D-F**, combinational treatment with metformin and Gboxin had no obvious effect on the proliferation of LO2 cells. Moreover, the migration/invasion of LO2 cells was not obviously changed either, in contrast to the anti-metastatic effects on HCC cells. These

findings suggest limited cytotoxicity of metformin plus Gboxin in noncancerous liver cells. We have added the relevant descriptions in the revised manuscript (**line 391-396**).

Appendix Fig. S5

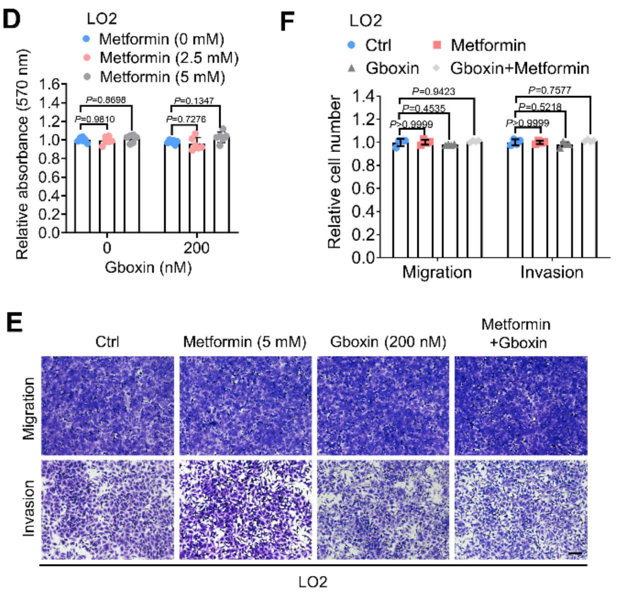

Appendix Fig. S5. (D) MTT assays showing the proliferation of liver non-cancerous cells LO2 treated with or without metformin or Gboxin at indicated concentration for 24 hours. (E-F) Transwell assays showing the migration and invasion of LO2 cells treated with or without metformin or Gboxin at indicated concentration for 24 hours. Scale bar, 100 μm.

6) Fig.3F: ECAR data should be presented.

Re: Thank you for your suggestion. This data has been already included in **Fig. S3E**, and now integrated into **Fig. 3F** in the revised manuscript.

Fig. 3

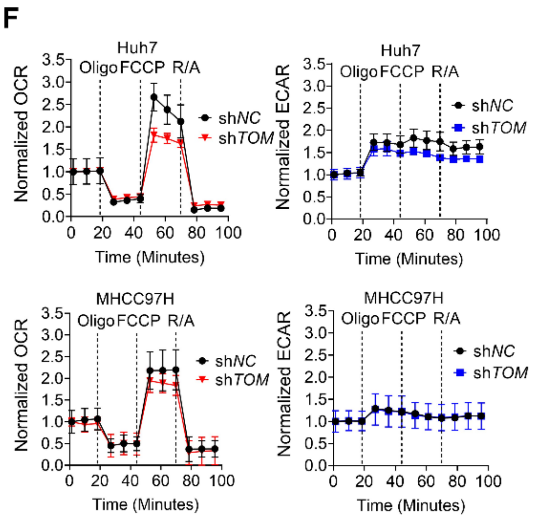

Fig. 3. (F) OCR and Extracellular Acidification Rate (ECAR) in Huh7 and HMCC97H cells with or without TOMM34 knockdown were measured using a Seahorse XF24 Extracellular Analyzer.

7) Fig.3G: Different exposures are needed for the different subunits.

Re: Thank you for your valuable comment. According to your kind suggestion, the different subunits of mitochondria were detected with different exposures (**Fig. 3G**). We also detected an additional complex I subunit NDUFB3 as shown in this figure. We have added the relevant description in the revised manuscript (**line 271**).

Fig. 3

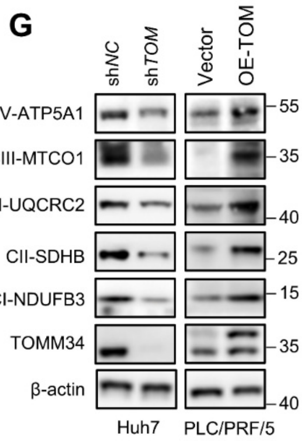

Fig. 3. (G) Western blot analysis showing the protein levels of the main mitochondrial respiratory chain complex subunit.

8) Fig.S1F and G: the differences in cell viability between the cell lines are striking- this should be discussed. Also, different concentrations of drugs or glucose should be tested as for certain conditions, the viability is lower than 50% at baseline.

Re: Thank you for your valuable comment. The differences in cell viability between the cell lines can be attributed to various factors. For example, PLC/PRF/5 cells are HBsAg positive, while Huh7 cells are not. HBsAg might regulate the viability of cells, leading to the differences between these two cell lines. Moreover, the genetic background (such as mutations of *TP53, ATM, etc.*) involved in regulating the viability of cells can be widely different among cell lines. However, this issue is out of the scope in this manuscript. As to the baseline level, Viable rates = [Number of living cells / (Number of living cells + Number of dead cells)] * 100%, the rates of live or death cells were calculated by comparing with each group. It is reasonable that the viability is lower than 50% in each group in **Fig. S1F-H** (**Appendix Fig. S1E-H in the revised manuscript**) as they were exposed to metabolic stress for 36 hours before subjecting to trypan blue staining. We also detected the baseline viability of cells without drug treatment, which was about 80-100% (**Response Fig. 6**).

Response Fig. 6

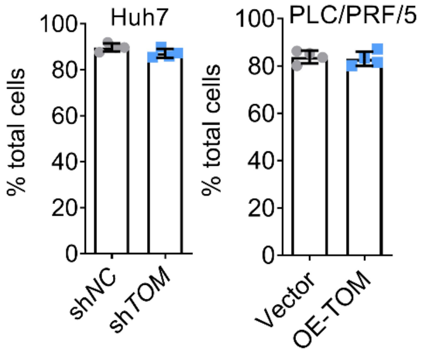

Response Fig. 6. Trypan blue staining assay showing the baseline viability of indicated cells.

Referee #2 (Remarks for Author):

The anti-tumor effects of metformin has been widely reported in recent years, however, drug resistance and tumor metastasis are also emerging during metformin treatment. In this manuscript, the authors observed the adaptation of HCC cells in long-term metformin exposure, which was characterized by an epithelial-mesenchymal transition (EMT) and tumor metastasis. Mechanically, the authors found that TOMM34 was up-regulated under metformin-induced metabolic stress and interacted with ATP5B to preserve ATPase activity, which then promoted OXPHOS and ATP production. As a result, the metabolic switch provided HCC cells the metastatic potential. More significantly, the authors elaborated that Gboxin, an OXPHOS inhibitor, could disturb the interaction of TOMM34 and ATP5B, thereby suppressing tumor survival and metastasis during metformin adaption, which may hold great clinical potential for HCC therapy. Overall, the findings are novel and interesting, and the experiments in general are clearly described and presented with conclusive data. Meanwhile, I have multiple concerns that the authors may want to address before publication.

1. The highlight of this study is that TOMM34/ATP5B-ATP synthesis axis plays an essential role in HCC metformin adaptation. However, a big part of experiments was performed in parental HCC cell lines, like in Figure.5A-5F/5H and Figure.6A/B. It's recommended to repeat some of the key experiments in the adaptive HCC cell lines used in this study;

Re: Thank you for your valuable comment. According to your kind suggestion, we repeated some of the key experiments, including Fig. 5A, C and Fig. 6B in the adaptive HCC cell lines. As shown in **Fig. 5A**, we found that ATP level of adaptive cells was decreased upon ATP5B knockdown. Moreover, knockdown of ATP5B inhibited the expression of mesenchymal markers such as Vimentin, Snail, Slug and ZEB1 in adaptive cells (**Fig. 5B-C**). In addition, we overexpressed exogenous ATP5B (Flag-

tagged) in TOMM34 knockdown metformin adaptive HCC cells, and found that the cellular ATP level, the expression of mesenchymal markers, and cell migration and invasion ability were significantly restored (**Fig. 5F-H**). Further, we also found that treatment of Gboxin disturbed the interaction between TOMM34 and ATP5B in adaptive cells (**Fig. 6B**). These data demonstrate that TOMM34/ATP5B-ATP synthesis axis played an essential role in HCC metformin adaptation. We have added the relevant descriptions in the revised manuscript (**line 335-338**).

Fig. 5

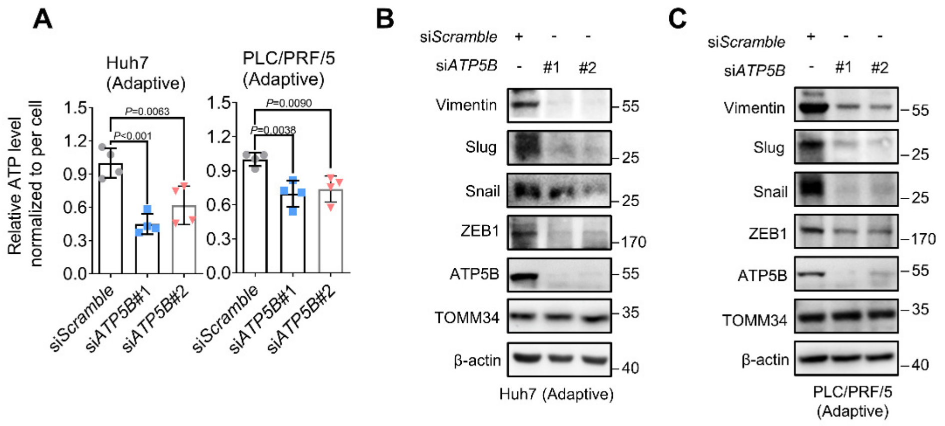

Fig. 5. (A) ATP level of adaptive HCC cells with or without ATP5B knockdown was measured by ATP detection kit. (B-C) Western blot shows the expression of EMT markers in adaptive HCC cells with or without ATP5B knockdown.

Fig. 5

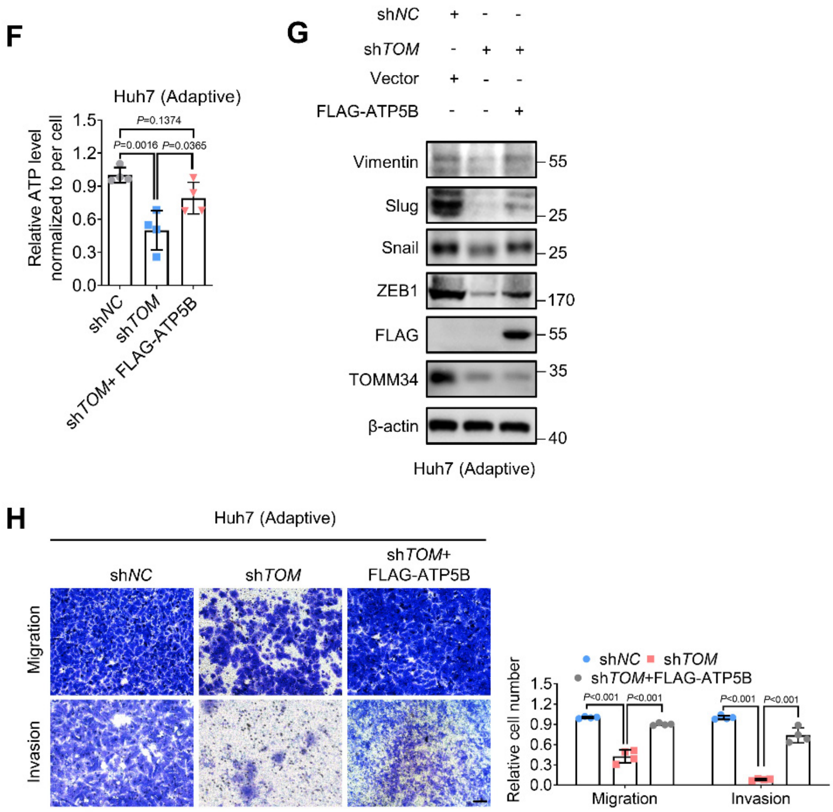

Fig. 5. (F) ATP level of indicated cells was measured by ATP detection kit. (G) Western blot showing the expression of EMT markers in cells with or without the knockdown of TOMM34 or overexpression of ATP5B. (H) Transwell assays showing the migration and invasion of indicated cells. Scale bar, 100 μm.

Fig. 6

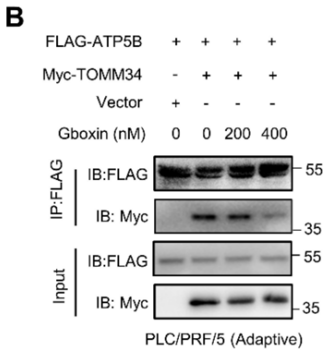

Fig. 6. (B) Co-immunoprecipitation assay showing the interaction between ATP5B and TOMM34 with or without Gboxin treatment.

2. For the functional experiments of this study, the authors only manipulated the TOMM34 knockdown in Huh7 cells and overexpression in PLC/PRF/5 cells to validate the pivotal role of TOMM34 during metformin adaptation. To further confirm the conclusion, the results of TOMM34 overexpression in Huh7 and knockdown in PLC/PRF/5 cells should be supplied;

Re: Thank you for your valuable comment. We detected the basal level of TOMM34 protein in two HCC cell lines, PLC/PRF/5 and Huh7. We found that Huh7 cells expressed higher TOMM34 protein level than PLC/PRF/5 cells, therefore Huh7 was used for TOMM34 knockdown experiments while PLC/PRF/5 was used for TOMM34 overexpression (**Response Fig. 4**). According to your kind suggestion, we generated TOMM34-overexpressed Huh7 cells and TOMM34-knockdown PLC/PRF/5 cells. Then, several key experiments were repeated using these two cell models, and the results were consistent with our previous findings. As shown in **Appendix Fig. S2A-E**, knockdown of TOMM34 in PLC/PRF/5 cells decreased the expression of mesenchymal markers, cellular ATP levels and inhibited cell migration and invasion, while overexpression of TOMM34 in Huh7 cells increased the expression of mesenchymal markers and promoted cell migration and invasion. We have added the relevant descriptions in the revised manuscript (**line 231-233**).

Response Fig. 4

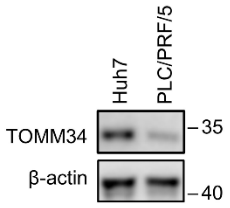

Response Fig. 4. Western blot showing basal level of TOMM34 protein in Huh7 and PLC/PRF/5 cells**.**

Appendix Fig. S2

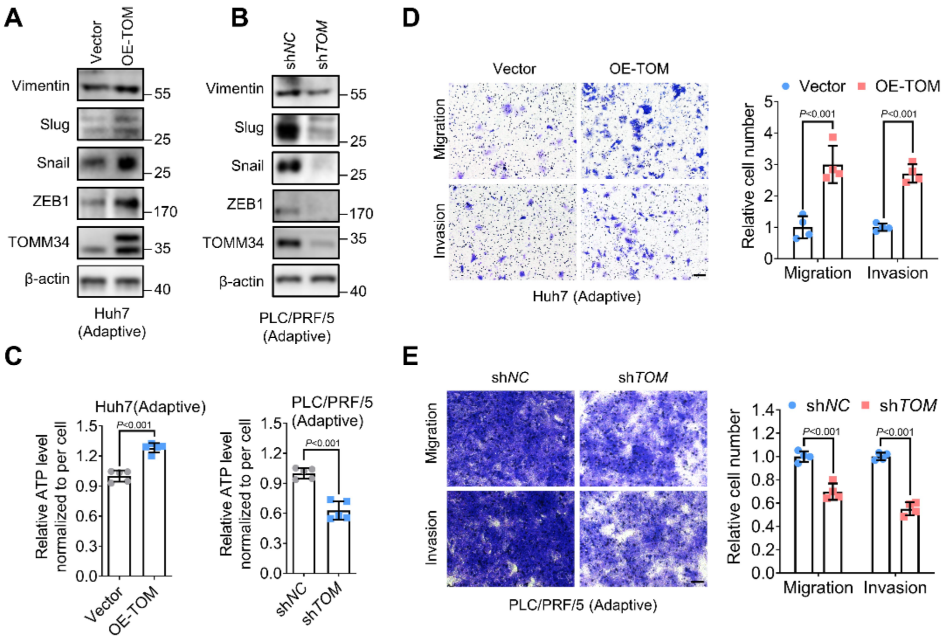

Appendix Fig. S2. (A-B) Western blot showing the expression of EMT markers in adaptive HCC cells with or without knockdown or overexpression of TOMM34. (C) Cellular ATP level of adaptive HCC cells with or without knockdown or overexpression of TOMM34. (D-E) Transwell assays showing the migration and invasion of adaptive HCC cells with or without knockdown or overexpression of TOMM34. Scale bar, 100 μm.

3. By integrating the MS results and the MitoCarta3.0 database, the authors identified

the TOMM34 as the key factor mediating the metformin adaptation. However, the heatmap in Fig.2B shows that it is TOMM34 that is greatly upregulated in the adaptive Huh7 cells, while in the adaptive PLC/PRF/5 cells is the ODPR. The authors may want to explain why they focus only on TOMM34;

Re: Thank you for your valuable comment. Metformin is a well-known energy metabolism-targeting drug, and mitochondria are main energy producers of the cell. Therefore, we speculated that certain mitochondrial proteins might be involved in metformin adaptation. ODPR, as quinoid dihydropteridine reductase, could catalyze the conversion of quinonoid dihydrobiopterin to tetrahydrobiopterin. While TOMM34 is a mitochondrial outer membrane protein, whose "family members" (e.g., TOMM 20, TOMM70) have been well documented to be involved in stress adaptation (Pitt & Buchanan, 2021; Zhou *et al*, 2018). For example, TOMM70-mediated cold-stress response by promoting cristae formation and respiration. Therefore, we decided to investigate the function of TOMM34 in metformin adaptation in this project.

4. In Fig.3G, the WB results didn't show the change of ATP5A1 and UQCRC2 expression upon the knockdown or over-expression of TOMM34. The authors may want to repeat and optimize the assays;

Re: Thank you for your valuable comment. This may be due to unoptimized exposure time. According to your kind suggestion, different subunits of mitochondria were detected solely and the results showed that ATP5A1 and UQCRC2 were both upregulated upon TOMM34 overexpression, while decreased when TOMM34 was knocked down (**Fig. 3G**). Besides, we also measured complex I subunit NDUFB3. This evidence suggests that TOMM34 increased the protein expression of several mitochondrial genes thus supporting the function of mitochondria, which was critical during metformin adaptation in HCC cells. We have added the relevant descriptions in the revised manuscript (**line 271**).

Fig. 3

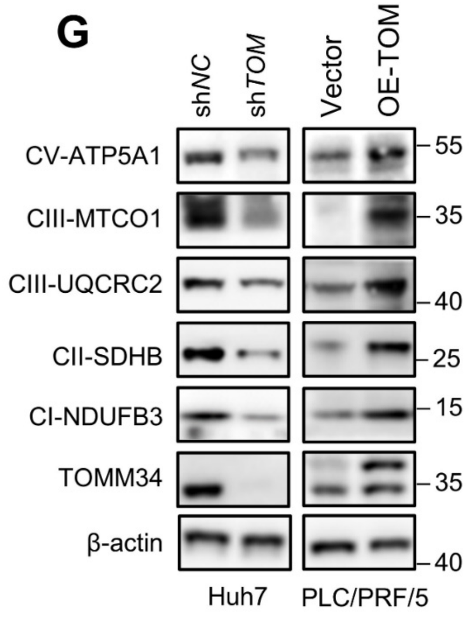

Fig. 3. (G) Western blot showing the protein levels of indicated mitochondrial complex genes in cells with or without TOMM34 knockdown or overexpression.

5. As authors described the over-expression of TOMM34 could up-regulate the expression of ATP5B in Fig.4E, however, the over-expression of Myc-TOMM34 seems to down-regulate the expression of Flag-ATP5B in Figure.6B. The authors may want to repeat and optimize the assays.

Re: Thank you for your valuable comment. The low expression of FLAG-ATP5B in original manuscript may be due to insufficient transfection efficacy. We repeated and optimized the assay in adaptive PLC/PRF/5 cells. The results suggested that treatment of Gboxin disturbed the interaction between TOMM34 and ATP5B in adaptive cells (**Fig. 6B**). These cells were transiently transfected with indicated plasmids (FLAG-ATP5B or Myc-TOMM34), leading to highly expressed protein level of both ATP5B and TOMM34, this may explain why ATP5B cannot be further upregulated by TOMM34.

Fig. 6

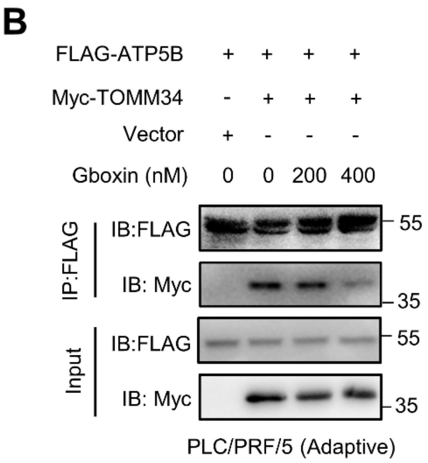

Fig. 6. (B) Co-immunoprecipitation assays showing the interaction between ATP5B and TOMM34 with or without Gboxin treatment.

Referee #3 (Comments on Novelty/Model System for Author):

Need to provide details on the characterization of the presumptive HCC models utilized.

Referee #3 (Remarks for Author):

The authors set to characterize the impact of metabolic adaptation to metformin treatment in HCC-derived patient-derived xenografts and cell lines. They showed that treatment of a subQ HCC PDX model with metformin resulted in a slight suppression of growth and an enhanced dissemination of these cells to the lung and liver. Using two HCC-derived cell lines exposed in vitro to increasing metformin concentrations, they selected populations that were able to grow in the presence of 5 mM metformin and showed a more "mesenchymal" phenotype. These cells also seemed to have higher ATP level compared to the parent population, while displaying an overall reduction of mitochondrial complex I activity, when a concomitant increase in complex V activity. These cells did not seem to compensate suppression of OXPHOS with enhanced glycolytic activity, as evidenced by lack of lactate accumulation. When comparing treated and parental cells by untargeted mass spectrometry, the authors identified TOMM34, a mitochondrial outer membrane protein as elevated in the treated cells, and verified its accumulation by immunoblot analysis. Accumulation of TOMM34 seemed to represent a functional adaptation to metformin treatment, as suppressing its accumulation by shRNA knockdown, affected survival of treated but not control cells. TOMM34 also seemed to protect cells from metabolic stress induced by 2-DG, as it might expected for a mitochondrial regulator, upon suppression of glycolysis.
The authors then tested whether TOMM34 accumulation might play a role in the dissemination phenotype consequent to metformin treatment and showed that in an orthotpic (liver) model, TOMM34 levels were elevated in lung lesions compared to the liver ones. TOMM34 suppression also seemed to induce an "epithelial-like" phenotype in another HCC cell line, and inhibited its dissemination to the lungs upon

tail vein injection. TOMM34 overexpression seemed to enhance lung dissemination. The authors also provide evidence of mesenchymal features and enhanced ability to migrate in TOMM34 overexpressing cells.

Upon RNA sequencing, the authors show that TOMM34 knockdown, compared to parental,cells, have significant alterations in OXPHOS pathway genes as also evidence by the presence of mitochondrial alteration by TEM. mtDNA, cellular ATP levels and OCR were all reduced upon TOMM34 suppression, as well as expression of mitochondrial OXPHOS complexes subunits, impacting complex I and V activity.

By IP-mass spec analysis the authors identified ATP5B as a TOMM34-interacting protein, with further evidence of interaction in HEK293 cells by co-IP of tagged proteins and evidence of interaction of the endogenous proteins in the Huh67 cells lines. Changes in TOMM34 levels by overexpression or knockdown seemed to concomitantly affect ATP5B levels.

The authors also show that TOMM34 and ATP5B co-colocalized in the invasive front area of cells in clinical samples and that both proteins were highly expressed in metastatic tissues. From the analysis of deletion mutants of TOMM34, they identify a 34-aa region responsible for its interaction with ATP5B. They then opted to determine whether suppression of ATP5B expression affected the ability of TOMM34 overexpression to sustain ATP accumulation and EMT. They also show that exogenous ATP supplementation restored EMT markers and dissemination ability upon TOMM34 knockdown. ATP5B silencing also inhibited migration and invasion capacity in vitro pf TOMM34 overexpressing cells as well that of metformin adapted cells. They also provide evidence that ATP5B expression correlates with worse prognosis upon analysis of available HCC pathology specimens.

They then evaluate whether a published covalent binder OXPHOS inhibitor, Gboxin, described as interacting with ATP5B, affected the interaction of TOMM34 with ATP5B and this was indeed the case. At concentrations that did not impact cell growth, Gboxin seemed to affect EMT marker expression and cell migration, while also impacting ATP accumulation upon TOMM4 overexpression.

Lastly, the authors provide evidence that Gboxin and metformin could be combined

leading to a synergistic effect on tumor growth and dissemination behavior, as also evidenced by a reduction of circulating tumor cell load in the PDX model.

The manuscript utilizes multiple experimental approaches to substantiate findings in support the authors claim that long-term effects of metformin treatment on HCC cells induce both an enhanced dissemination ability and deregulation of mitochondrial function via upregulation of certain mitochondrial proteins, which compensates for the effects of the drug on oxidative phosphorylation. While intriguing, I believe that much more work would be needed to solidify these findings, whether this applies specifically to hepatocellular carcinoma, whether the models utilize recapitulate the genetics and cell of origin of the diseases, whether the observed effect are specific to the drug being tested, a recognized polypharmacy entity, what the mechanism are of enhanced migration and dissemination. I would perhaps refocus the manuscript around a defined set of key findings. Breadth of scope should not be an alternative to depth of scope.

1. Better characterization of cell and PDX models under use should be provided. Antibody validation should be provided in the context of knockdown for immunochemistry/immunofluorescence.

Re: Thank you for your valuable comment. Here we provided the certificate for our cell lines used in this study (**Response Fig. 7A-B**). Besides, we also performed H&E and immunochemistry analysis to validate our PDX models (**Response Fig. 7C**), which showed characteristic histology of liver cancer and positive staining of liver cancer markers such as CK19 and CD90. Moreover, antibody validation was also performed by immunofluorescence in sh*NC* and TOMM34 knockdown (sh*TOM*) cells to validate the specificity of TOMM34 antibody used in this study (**Response Fig. 7D)**. We also detected the TOMM34 protein levels using IHC assay in vehicle or metformin-treated PDX tissues. These results indicated that TOMM34 was upregulated in metformin-treated tissues (**Fig. EV2A**). We have added the relevant descriptions in the revised manuscript (**Line 206-207**).

Response Fig. 7

A

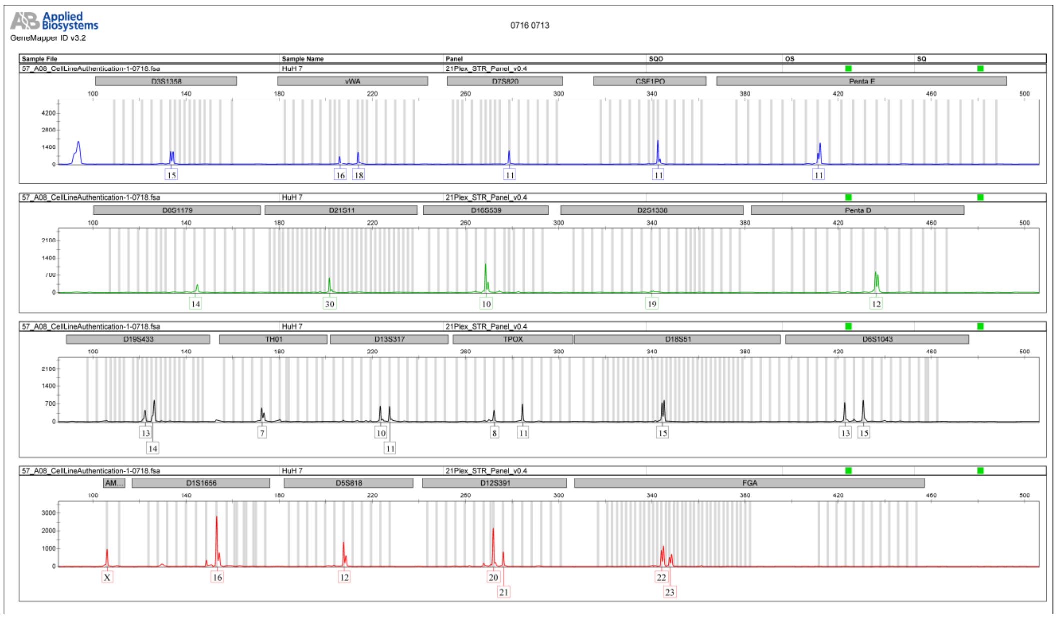

B

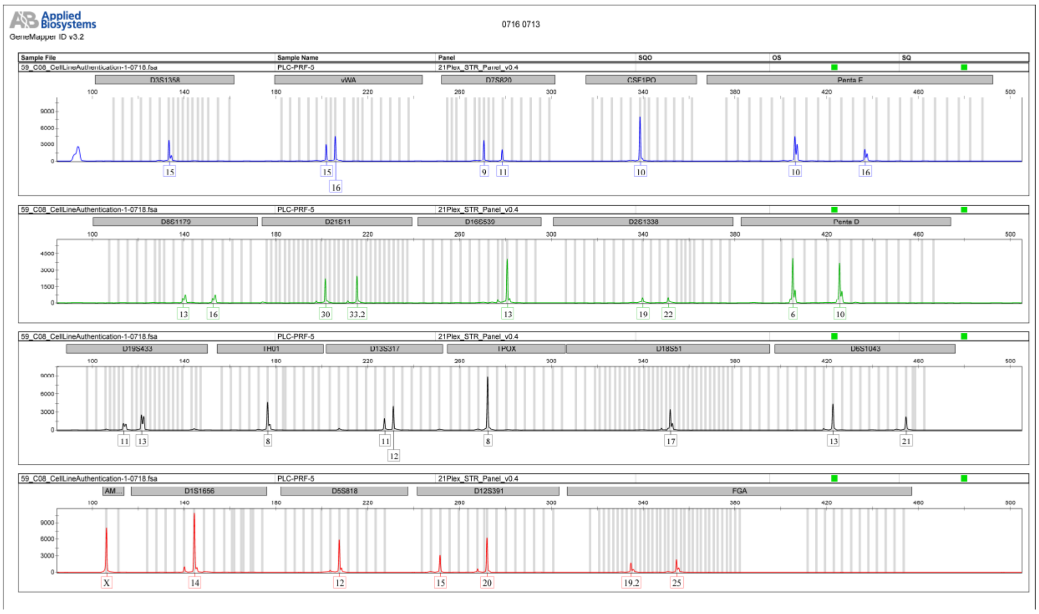

Response Fig. 7

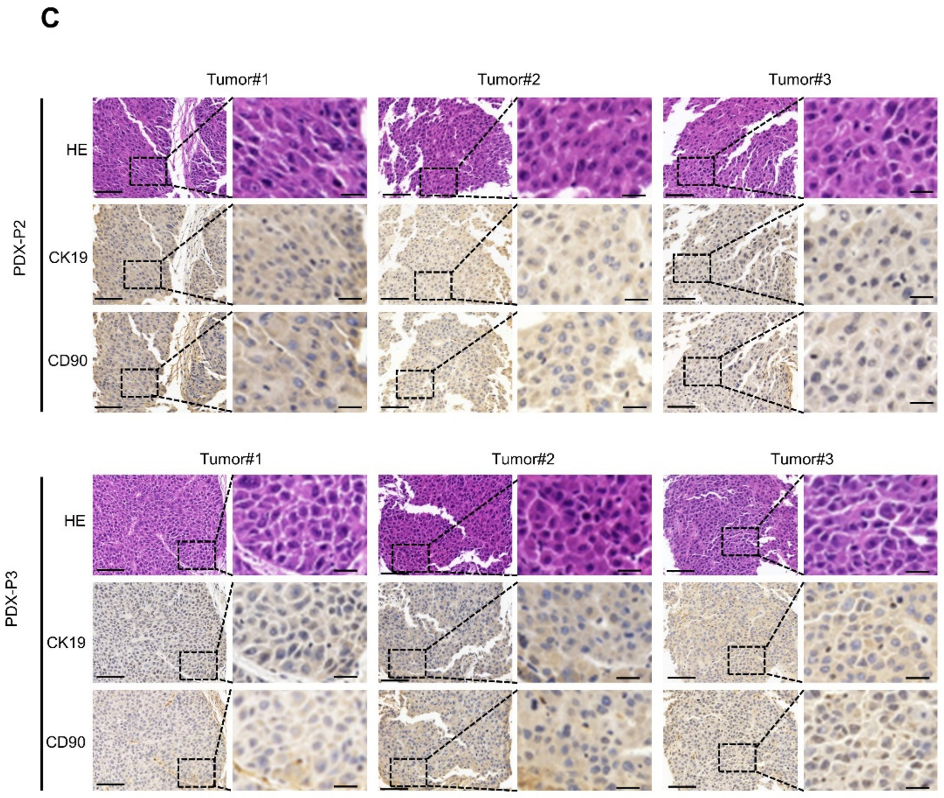

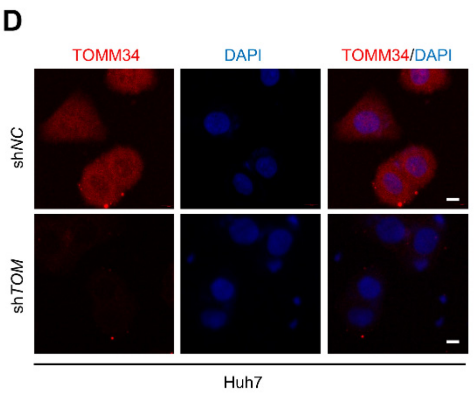

Fig. EV2

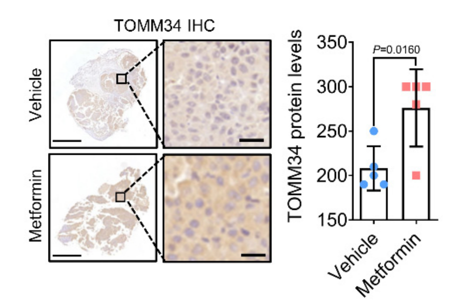

Response Fig. 7. (A-B) STR identification of Huh7 and PLC/PRF/5 cells using Expasy database. (C) H&E staining of PDX tumors to show the histology of liver cancer tissues. Immunohistochemistry image for the expression of HCC markers CK19 and CD90 were shown. Scale bar, 100 μm or 25 μm. (D) Antibody validation by immunofluorescent detection in HCC cells with or without TOMM34 knockdown. Scale bar, 10 μm.

Fig. EV2. (A) The protein levels of TOMM34 in PDX models with or without

metformin treatment were evaluated by immunohistochemistry. Scale bar, (Left) 1000 µm; (Right) 100 µm.

2. Is the described "adaptative" mechanism via the TOMM34/ATP5B axis specifically related to complex I inhibition? The authors may want to test other complex I inhibitors, such as phenformin or others, in TOMM34 or ATP5B deficient adaptive HCC cells.

Re: Thank you for your valuable comment. To ascertain if the adaptation mechanism via the TOMM34/ATP5B axis was specifically related to complex I inhibition, we performed additional assays via exposure to rotenone (complex I inhibitor). As shown in **Response Fig. 8A-C**, knockdown of TOMM34 enhanced the metastatic inhibition effect of rotenone, while displaying no obvious influence on the metastatic inhibition effect of antimycin or oligomycin (complex III, V inhibitors, respectively) in HCC cells. Moreover, TOMM34 was upregulated after 24 hours-removal from 4-days' rotenone treatment (**Response Fig. 8D**). This evidence suggests that the TOMM34/ATP5B axis was probably a general adaptive mechanism related to complex I inhibition.

Response Fig. 8

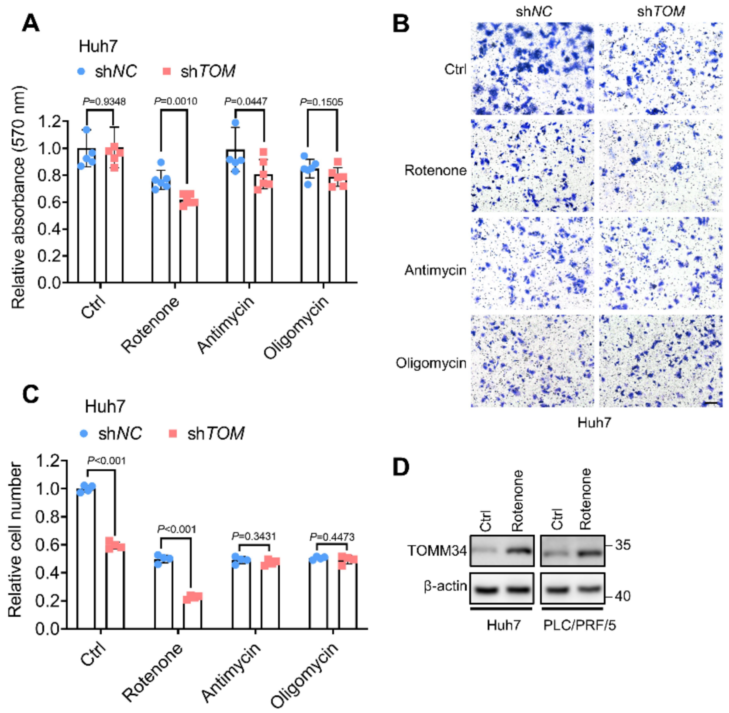

Response Fig. 8. (A) MTT assays showing the proliferation of sh*NC* or sh*TOM* cells treated with indicated mitochondrial inhibitors (0.1 μM, 24 hours). The rotenone, antimycin and oligomycin were used as the inhibitor of mitochondrial complex I, III, V, respectively. (B-C) Transwell assays showing the migration and invasion ability of sh*NC* and sh*TOM* cells treated with indicated mitochondrial complex inhibitors (0.1 μM, 36 hours). Scale bar, 100 μm. (D) Western blot showing the expression of TOMM34 in Huh7 and PLC/PRF/5 cells after 24 hours-removal from 4-days' rotenone treatment.

3. Conversely, would this mechanism apply to other mitochondrial inhibitors, e.g. antimycin (Complex III) or oligomycin (complex V)?

Re: Thank you for your valuable comment. According to your kind suggestion, we evaluated presumptive adaptation of HCC cells to antimycin and oligomycin. As shown in **Response Fig. 8A-C**, knockdown of TOMM34 had no obvious effect on enhancing antimycin or oligomycin-mediated metastatic inhibition. This evidence

suggested that the TOMM34/ATP5B axis may be specifically related to complex I inhibition instead of other mitochondrial complex inhibitors, at least not apply to antimycin (complex III) and oligomycin (complex V).

Response Fig. 8

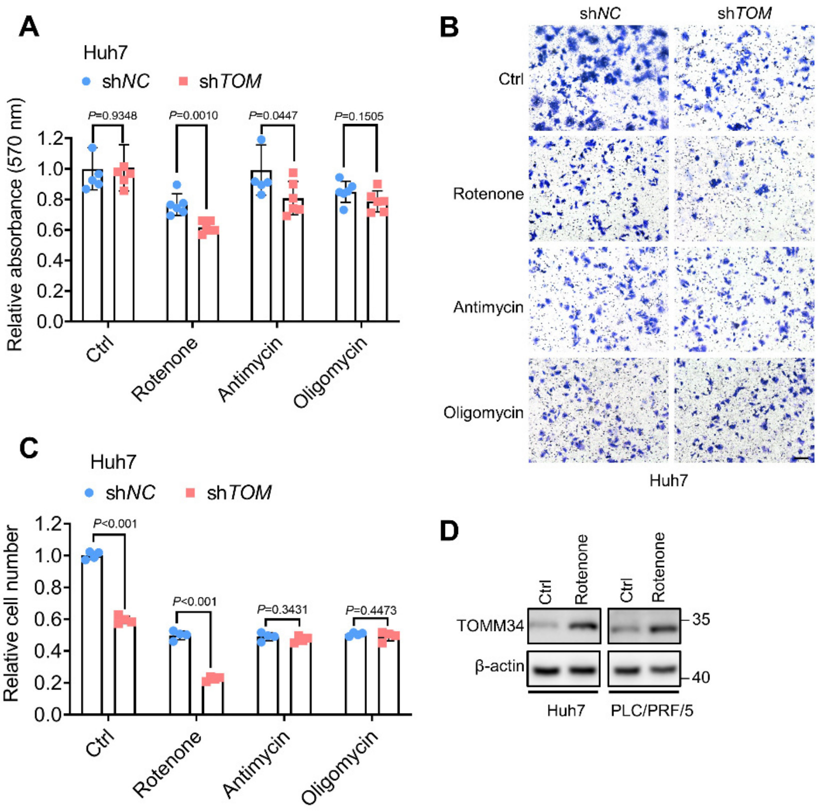

Response Fig. 8. (A) MTT assays showing the proliferation of sh*NC* or sh*TOM* cells treated with indicated mitochondrial inhibitors (0.1 µM, 24 hours). The rotenone, antimycin and oligomycin were used as the inhibitor of mitochondrial complex I, III, V, respectively. (B-C) Transwell assays showing the migration and invasion ability of sh*NC* and sh*TOM* cells treated with indicated mitochondrial complex inhibitors (0.1 µM, 36 hours). Scale bar, 100 µm. (D) Western blot showing the expression of TOMM34 in Huh7 and PLC/PRF/5 cells after 24 hours-removal from 4-days' rotenone treatment.

4. In cells exposed to long term treatment with metformin, you may need to evaluate

any changes in the activity of other mitochondrial complexes, not just Complex I and V (Fig1J and K).

Re: Thank you for your valuable comment. According to your kind suggestion, we tested the activity of other mitochondrial complexes after exposure to long-term treatment of metformin. As shown in **Fig. EV1C-E**, the activity of other mitochondrial complexes (including II, III and IV) in adaptive cells was preserved at a relatively high level compared with those in parental cells. These results were consistent with our findings about complex I and V, indicating that mitochondrial function was enhanced in adaptive cells. We have added the relevant descriptions in the revised manuscript (**line 187-189**).

Fig. EV1

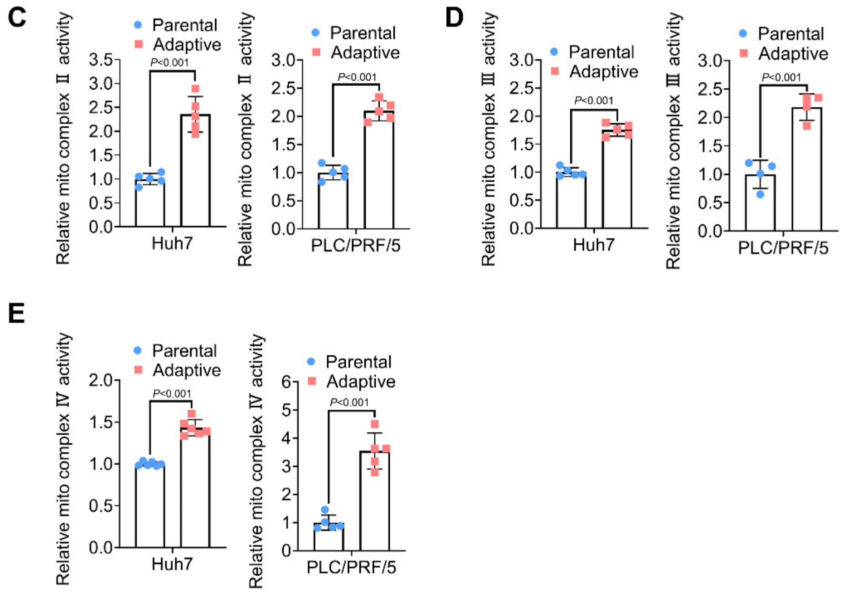

Fig. EV1. (C-E) The activity of mitochondrial complex II, III and IV in parental and adaptive HCC cells was measured.

5. Are there any changes in OCR and ECAR upon short-term (24hr) vs long-term treatment? Are TOMM34 protein levels or its interaction with ATP5B increased upon short term treatment (24hr)?

Re: Thank you for your valuable comment. According to your kind suggestion, we

evaluated the OCR and ECAR upon short-term treatment (24 hours, Parental + met) and long-term treatment (Adaptive + met). As shown in **Response Fig. 9A-B**, OCR and ECAR were decreased in response to both short-term (24 hours, Parental + met) and long-term metformin treatment (Adaptive + met), while the extent of decrease upon long-term treatment (Adaptive + met) was smaller than that upon short-term treatment (24 hours, Parental + met), suggesting a preserved mitochondrial function in adaptive cells. The TOMM34 protein level and its interaction with ATP5B were not obviously changed upon short-term metformin treatment (**Response Fig. 9C**).

Response Fig. 9

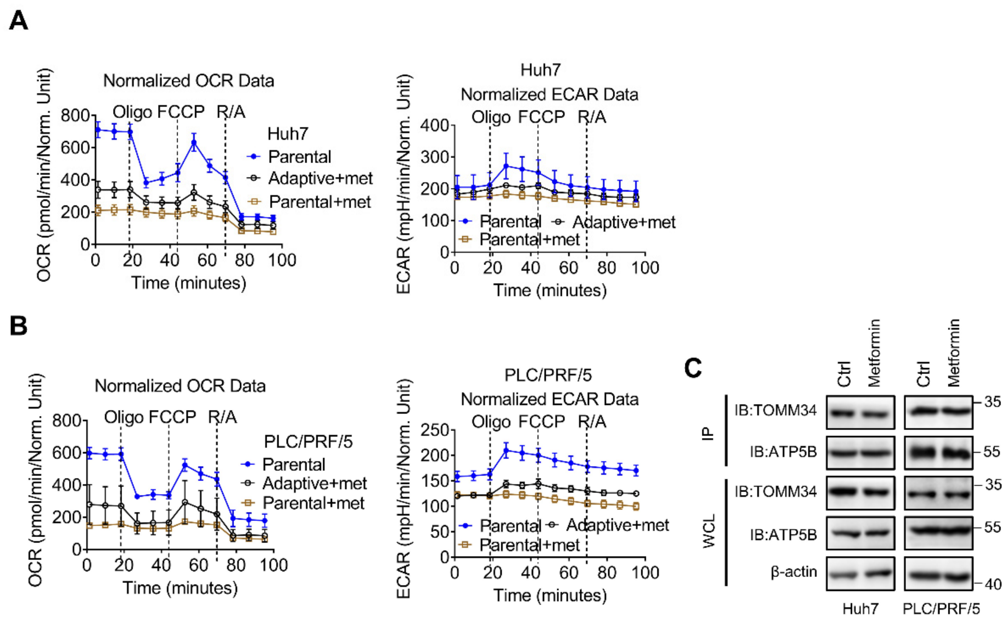

Response Fig. 9. (A-B) OCR and ECAR in indicated cells were measured using a Seahorse XF24 Extracellular Analyzer. (Adaptation + met represents long-term metformin treatment. Parental + met represents short-term metformin treatment (24 hours)). (C) Co-immunoprecipitation assays showing the interaction between TOMM34 and ATP5B in HCC cells treated with or without short-term metformin treatment.

6. Metformin targets multiple signaling pathways including AMPK, mTOR, IGF signaling, and mitochondrial complex I. In your evaluation of MS data with the

MitoCarta 3.0 database, which is specific for mitochondrial related genes, might you have missed on an opportunity to look more broadly at adaptive mechanisms (Fig2A-B)? How should we think about other non-mitochondrial mediated effects of metformin treatment in this context?

Re: Thank you for your valuable comment. When analyzing MS data, we noticed several other signaling pathways (e.g., insulin signaling pathway; proteosome, lysosome associated signaling pathway) are enriched in adaptive HCC cells, suggesting that non-mitochondrial adaptive mechanisms might exist. Although metformin targets other signaling pathways, such as AMPK, mTOR and IGF, it is interesting that these signaling pathways are also linked with mitochondrial function (Bhardwaj *et al*, 2021; Cunningham *et al*, 2007; Herzig & Shaw, 2018). Even if the non-mitochondrial mechanisms exist, these issues are out of the scope of this manuscript and would be investigated in the future studies. Previously published papers showed the potential role of reactivated OXPHOS in regulating metformin adaptation (Lord *et al*, 2018). Therefore, we mainly focused on the mitochondrial proteins in this manuscript. We have also provided all MS data from parental or adaptive cells without using MitoCarta 3.0 dataset as source data for further study.

7. Are there any other metabolic changes in cell exposed to long term metformin treatment versus parental HCC cells? And does TOMM34 overexpression in these cells induce similar changes?

Re：Thank you for your valuable comment. According to your kind suggestion, we explored our proteomics data and found that pyruvate metabolism was another changed pathway in cells exposed to long-term metformin treatment, although it was less significantly enriched compared with mitochondrial metabolic pathways (**Response Fig. 10A**). To verify this, we detected pyruvate level in adaptive and parental HCC cells, where we found a decrease of pyruvate level in adaptive HCC cells (**Response Fig. 10B**), which was consistent with our proteomics data. However, TOMM34 knockdown or overexpression failed to induce similar changes (**Response Fig. 10C)**, suggesting that TOMM34 displayed no obvious effect on pyruvate level

during metformin adaptation.

Response Fig. 10

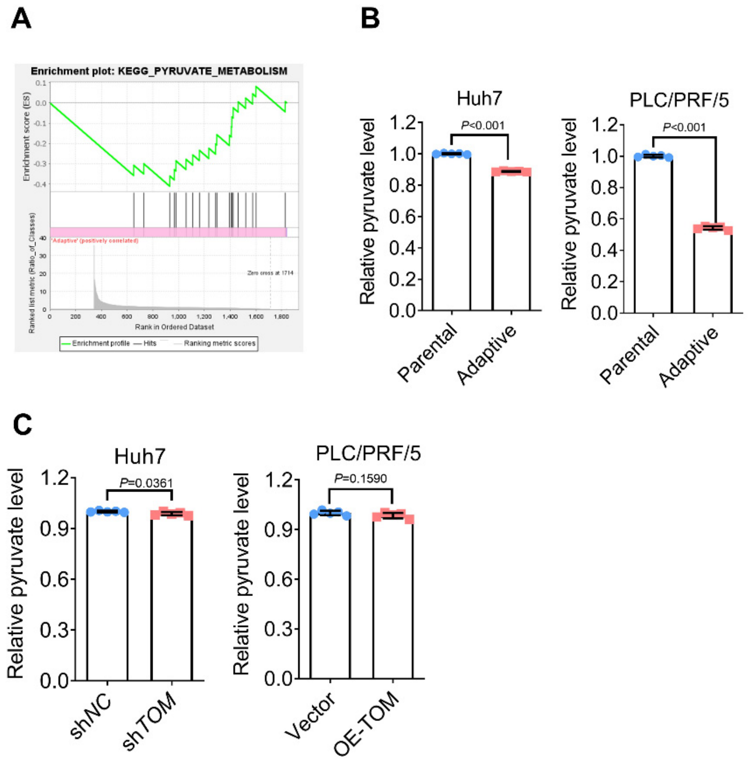

Response Fig. 10. (A) KEGG analysis showing the enrichment of pyruvate metabolism pathway during metformin adaptation. (B) Pyruvate level in parental and adaptive HCC cells was measured. (C) Pyruvate level in adaptive HCC cells with or without the knockdown or overexpression of TOMM34 was measured.

8. In Fig3G, mitochondrial CII and CIII level, rather than CV, were significantly regulated by TOMM34 KD or OE. How should this be interpreted? Mitochondrial biosynthesis related? Are CII and CIII activities increased? Why there is no CI band?

Re： Thank you for your valuable comment. This may be due to unoptimized exposure time. According to your kind suggestion, different subunits of mitochondria were detected solely with different exposure time and the results showed that ATP5A1 and UQCRC2 were both upregulated upon the TOMM34 overexpression, while decreased when TOMM34 was knocked down. Interestingly, we found the activities of CII, CIII

and CIV were all increased upon TOMM34 overexpression while decreased upon TOMM34 knockdown (**Response Fig. 11A-C**), which may attribute to that TOMM34 can affect one or more complexes, or one affected complex may further influence the activities of other complexes, as it has been reported that complex II was activated by complex III deficiency-induced redox unbalance (Tropeano *et al*, 2018). Besides, we also provided additional data to illustrate the expression of CI by immunoblotting NDUFB3 (**Fig. 3G**), and the results suggest that the protein level of NDUFB3 was upregulated upon TOMM34 overexpression, while decreased when TOMM34 was knocked down. These data suggest that TOMM34 may regulate mitochondrial function through modulating the protein level and activities of mitochondrial complexes. We have added the relevant descriptions in the revised manuscript (**line 271**).

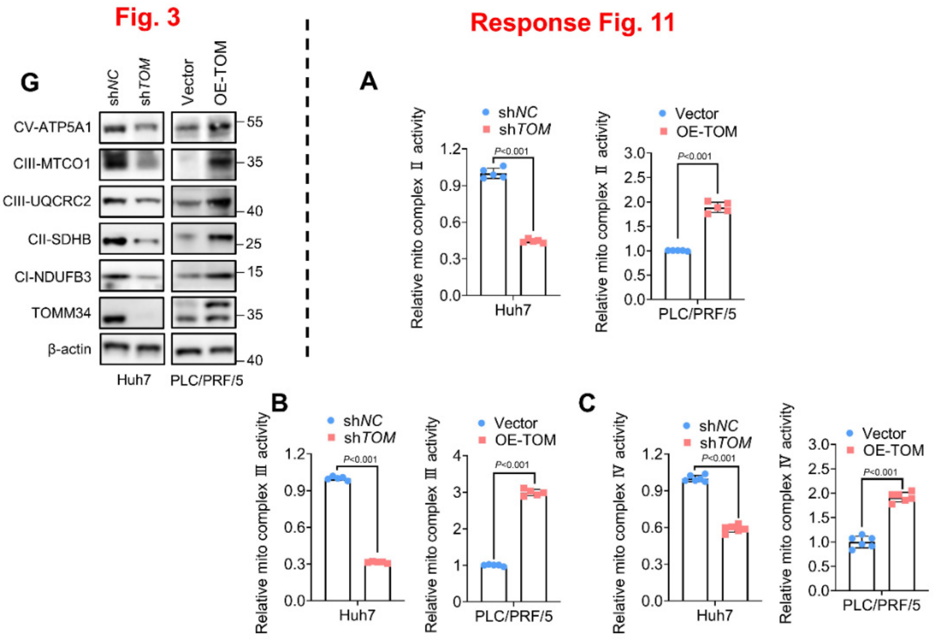

Fig. 3. (G) Western blot showing the protein level of indicated mitochondrial complex genes in cells with or without knockdown or overexpression of TOMM34.

Response Fig. 11. (A-C) The activity of mitochondrial complex II (A), III (B) and IV (C) in cells with or without knockdown or overexpression of TOMM34 was measured.

9. In the heatmap of Fig4E, OE-TOM group showed decreased TOMM34, inconsistent with western blotting data.

Re： Thank you for your valuable comment. This may be due to the unoptimized image processing software. We have exported the results with optimized parameters (**Fig. 4E**).

Fig. 4

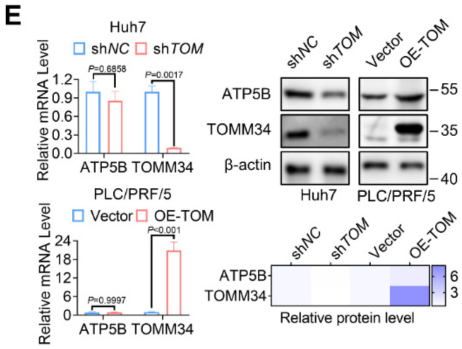

Fig. 4. (E) The mRNA and protein expression of ATP5B in indicated cells were determined by qPCR or immunoblotting.

10. TOMM34 is an outer mitochondrial membrane translocase, while ATP5B is a subunit of ATP synthase which is located in the inner membrane and faces the mitochondrial matrix. How could they interact with each other so tightly (Fig4C)?

Re： Thank you for your valuable comment. During mitochondrial dynamics, proteins located in different parts of mitochondria might alter their location transiently, thus forming protein complex which is vital for their biological functions (Chai *et al*, 2021). Actually, although absent in nucleus, ATP5B has been reported to interact with FUS, a DNA/RNA binding protein that usually locates in the nucleus (Deng *et al*, 2018). These protein translocation events reflect the complexity of biological processes, though this issue is not detailed in our manuscript.

11. To prove the relevance of TOMM34/ATP5B interaction, you may need to utilize the TPR4 region-truncated protein to perform migration and invasion rescue

experiments. (FigS4)

Re：Thank you for your valuable comment. According to your kind suggestion, we utilized the TPR4-deletion Myc-tagged-TOMM34 (Myc-TOM-TPR4 del) protein to perform migration and invasion rescue experiments. As shown in **Fig. EV3C-E**, overexpression of full-length Myc-tagged TOMM34 (Myc-TOM-FL) restored the migration and invasion of endogenous TOMM34 knockdown HCC cells, while the Myc-TOM-TPR4 del failed to do so, indicating that TPR4-mediated TOMM34/ATP5B interaction was required for increased migratory and invasive capacities of metformin-adaptive HCC cells. We have added the relevant descriptions in the revised manuscript (**line 318-326**).

Fig. EV3

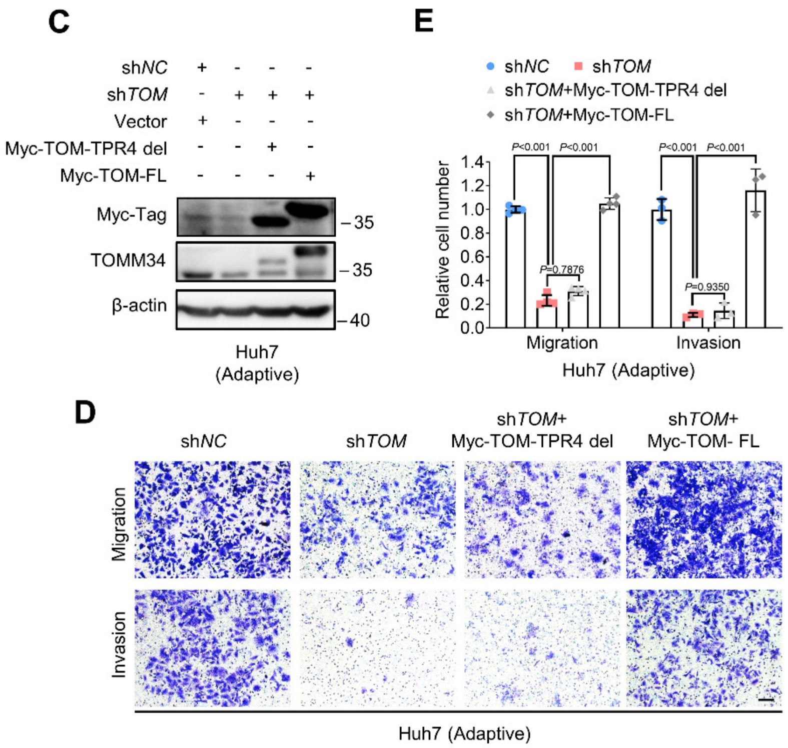

Fig. EV3. (C) Western blot showing the expression of full-length or TPR4-truncated TOMM34 in adaptive Huh7 sh*TOM* cells. (D-E) Transwell assays showing the migration and invasion of HCC cells expressing full-length or TPR4-truncated TOMM34. Scale bar, 100 μm.

12. Would migration or invasion be rescued by ATP5B expression in TOMM34 knockdown HCC cells?

Re：Thank you for your valuable comment. According to your kind suggestion, we overexpressed exogenous ATP5B in TOMM34 knockdown HCC cells, and found the cellular ATP level, expression of mesenchymal markers and their migratory and invasive abilities were significantly restored (**Fig. 5F-H**). This result indicats that the migration and invasion can be partially rescued by ATP5B overexpression in TOMM34 knockdown HCC cells. We have added the descriptions of **Fig. 5F-H** in the revised manuscript (**Line 335-338**).

Fig. 5

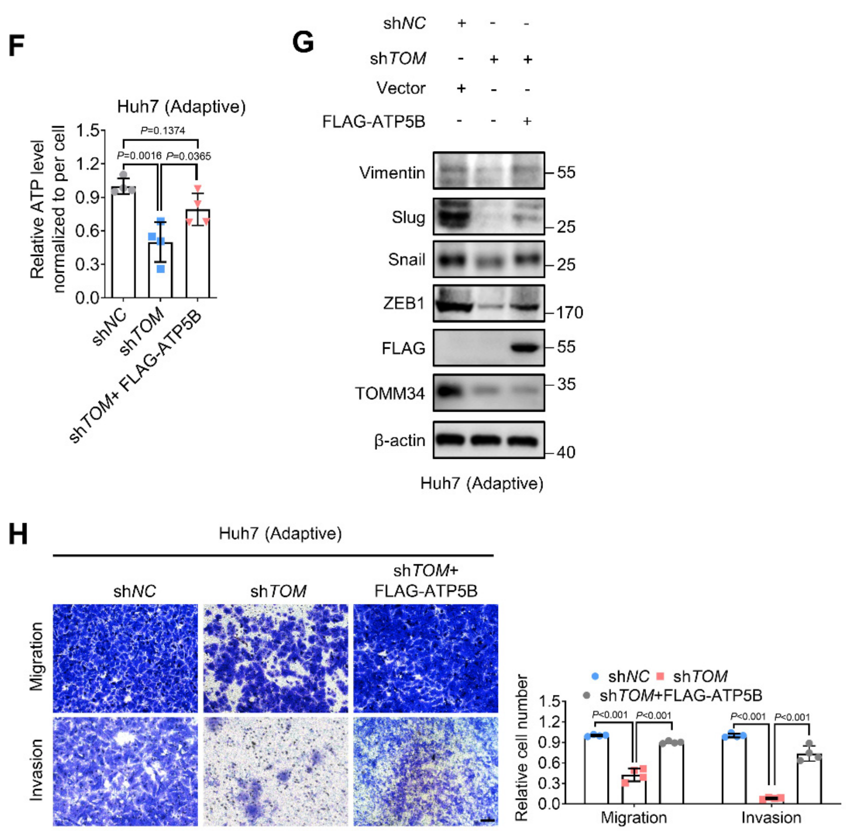

Fig. 5. (F) ATP level of indicated cells was measured by ATP detection kit. (G) Western blot showing the expression of EMT markers in cells with or without knockdown of

TOMM34 or overexpression of ATP5B. (H) Transwell assays showing the migration and invasion of indicated cells. Scale bar, 100 μm.

13. In Fig6I, a Gboxin alone group should be added to prove the advantage of combination effects in tumor suppression.

Re: Thank you for your valuable comment. According to your kind suggestion, we have added a S-Gboxin alone group to prove the advantage of combination effects. BALB/c nude mice were subcutaneously injected with adaptive Huh7 cells and administered with vehicle, S-Gboxin, metformin or metformin in combination with S-Gboxin. As shown in **Fig. 6H**, the S-Gboxin alone group and metformin alone group exhibited no significant antitumor effect, while metformin plus S-Gboxin exhibited robust anticancer efficacy. This result suggests the combinational treatment of S-Gboxin with metformin can be a potential anticancer strategy in adaptive HCC cells. We have added the relevant descriptions in the revised manuscript (**line 382-387**).

Fig. 6

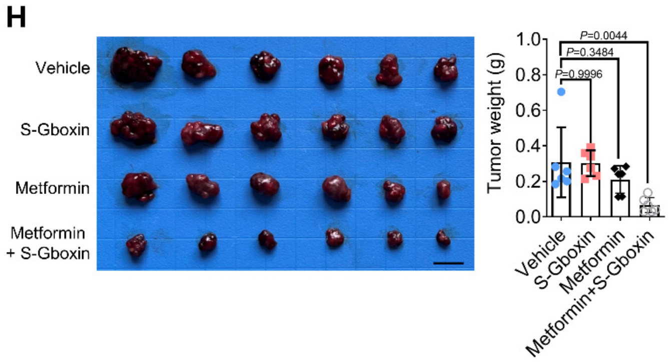

Fig. 6. (H) Adaptive Huh7 cells were subcutaneously injected into mice and then mice were received Gboxin, metformin or combinational treatment. The image and weight of tumor xenografts were shown. Scale bar, 1 cm.

14. Gboxin was found to inhibit OXPHOS inhibitor by inhibition of the F0F1 ATP synthase in GBM. This covalent interactor also binds other components of mitochondrial complexes (Nature volume 567, pages341-346 (2019)). In the context

of that manuscript there seem to be no evidence of interaction with TOMM34/ATP5B. Is it possible therefore that Gboxin directly inhibits ATP production rather than disturbing interaction of TOMM34 and ATP5B? Please verify it.

Re：Thank you for your valuable comment. The Nature paper described that Gboxin could directly bind with ATP5B (Shi *et al*, 2019). In our view, the finding in Nature paper did not preclude the possibility of TOMM34/ATP5B interaction. In fact, it is possible that TOMM34 and Gboxin may competitively bind with ATP5B. In the absence of Gboxin, TOMM34 interacted with ATP5B to enhance mitochondrial function thus playing critical roles in metabolic adaptation and tumor progression. In contrast, in the presence of Gboxin, Gboxin could interact with ATP5B, leading to the dissociation of ATP5B and TOMM34, resulting in the degradation of ATP5B and compromised mitochondrial function. This was consistent with our findings that Gboxin could inhibit the migration and invasion of HCC cells while having no obvious impact on the metastasis of TOMM34-deficient Huh7 cells (**Fig. 6E** and **Appendix Fig. S5C**). In other words, our finding is not conflicting with that in the Nature paper. To further verify this, we determined the ATP level in sh*NC*/sh*TOM* HCC cells with Gboxin treatment. As shown in **Response Fig. 12**, Gboxin decreased the ATP level in sh*NC* cells. However, the ATP production in sh*TOM* cells was not significantly changed, suggesting that Gboxin inhibited ATP production at least partially relying on TOMM34.

Fig. 6

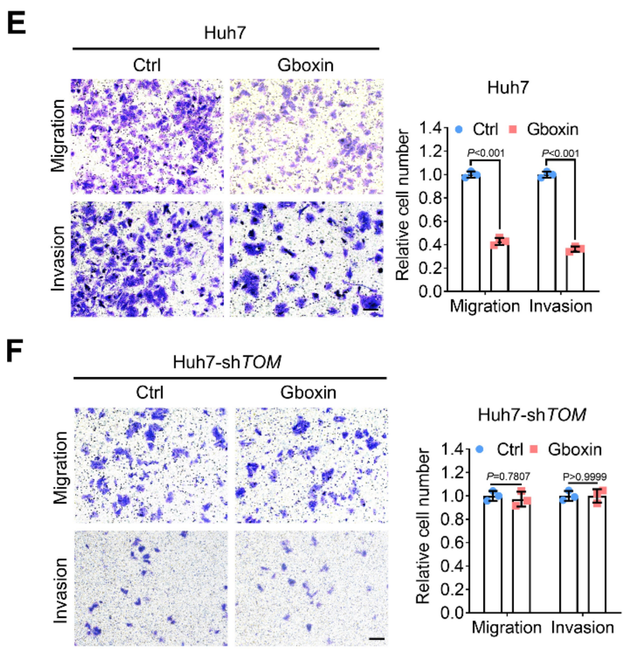

Fig. 6. (E-F) Transwell assay showing the migration of HCC cells treated with or without 200 nM Gboxin for 36 hours. Scale bar, 100 μm.

Appendix Fig. S5

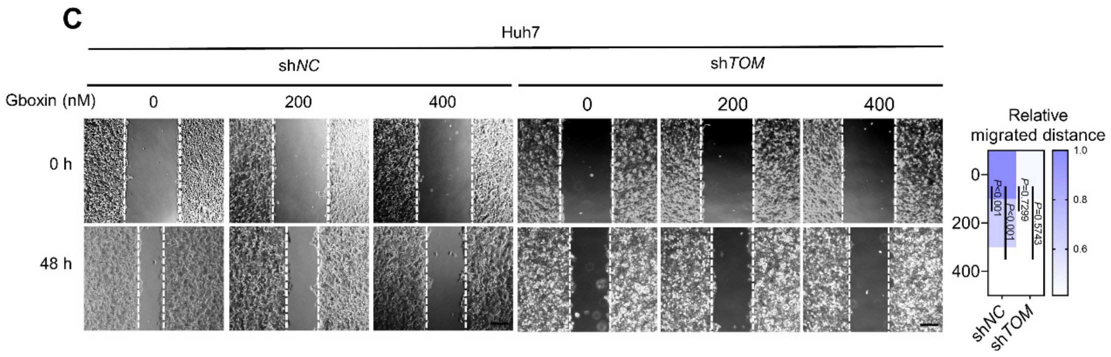

Appendix Fig. S5. (C) Wound healing assay showing the migration of Huh7 sh*NC* and sh*TOM* cells treated with or without Gboxin as indicated. Scale bars, 200 μm.

Response Fig. 12

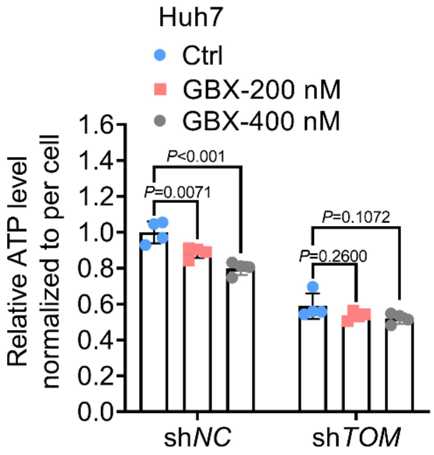

Response Fig. 12. ATP level in sh*NC* and sh*TOM* cells treated with Gboxin (GBX) at indicated concentration was measured.

References

Bhardwaj G, Penniman CM, Jena J, Suarez Beltran PA, Foster C, Poro K, Junck TL, Hinton AO, Souvenir R, Fuqua JD *et al* (2021) Insulin and IGF-1 receptors regulate complex I-dependent mitochondrial bioenergetics and supercomplexes via FoxOs in muscle. *J Clin Invest* 131

Chai P, Cheng Y, Hou C, Yin L, Zhang D, Hu Y, Chen Q, Zheng P, Teng J, Chen J (2021) USP19 promotes hypoxia-induced mitochondrial division via FUNDC1 at ER-mitochondria contact sites. *J Cell Biol* 220

Cunningham JT, Rodgers JT, Arlow DH, Vazquez F, Mootha VK, Puigserver P (2007) mTOR controls mitochondrial oxidative function through a YY1-PGC-1alpha transcriptional complex. *Nature* 450: 736-740

Deng J, Wang P, Chen X, Cheng H, Liu J, Fushimi K, Zhu L, Wu JY (2018) FUS interacts with ATP synthase beta subunit and induces mitochondrial unfolded protein response in cellular and animal models. *Proc Natl Acad Sci U S A* 115: E9678-E9686

Herzig S, Shaw RJ (2018) AMPK: guardian of metabolism and mitochondrial homeostasis. *Nat Rev Mol Cell Biol* 19: 121-135

Huang Z, Xie N, Illes P, Di Virgilio F, Ulrich H, Semyanov A, Verkhratsky A, Sperlagh B, Yu S-G, Huang C *et al* (2021) From purines to purinergic signalling: molecular functions and human diseases. *Signal Transduct Target Ther* 6: 162

LeBleu VS, O'Connell JT, Gonzalez Herrera KN, Wikman H, Pantel K, Haigis MC, de Carvalho FM, Damascena A, Domingos Chinen LT, Rocha RM *et al* (2014) PGC-1α mediates mitochondrial biogenesis and oxidative phosphorylation in cancer cells to promote metastasis. *Nat Cell Biol* 16

Lehuédé C, Dupuy F, Rabinovitch R, Jones RG, Siegel PM (2016) Metabolic Plasticity as a Determinant of Tumor Growth and Metastasis. *Cancer Res* 76: 5201-5208

Lord SR, Cheng W-C, Liu D, Gaude E, Haider S, Metcalf T, Patel N, Teoh EJ, Gleeson F, Bradley K *et al* (2018) Integrated Pharmacodynamic Analysis Identifies Two Metabolic Adaption Pathways to Metformin in Breast Cancer. *Cell Metab* 28

Pitt AS, Buchanan SK (2021) A Biochemical and Structural Understanding of TOM Complex Interactions and Implications for Human Health and Disease. *Cells* 10

Sharma S, Kalra H, Akundi RS (2020) Extracellular ATP Mediates Cancer Cell Migration and Invasion Through Increased Expression of Cyclooxygenase 2. *Front Pharmacol* 11: 617211

Shi Y, Lim SK, Liang Q, Iyer SV, Wang H-Y, Wang Z, Xie X, Sun D, Chen Y-J, Tabar V *et al* (2019) Gboxin is an oxidative phosphorylation inhibitor that targets glioblastoma. *Nature* 567: 341-346

Tropeano CV, Fiori J, Carelli V, Caporali L, Daldal F, Ghelli AM, Rugolo M (2018) Complex II phosphorylation is triggered by unbalanced redox homeostasis in cells lacking complex III. *Biochim Biophys Acta Bioenerg* 1859: 182-190

Zhou B, Zhang J-Y, Liu X-S, Chen H-Z, Ai Y-L, Cheng K, Sun R-Y, Zhou D, Han J, Wu Q (2018) Tom20 senses iron-activated ROS signaling to promote melanoma cell pyroptosis. *Cell Res* 28: 1171-1185

6th Sep 2022

Dear Dr. Huang,

Thank you for the submission of your manuscript to EMBO Molecular Medicine and please accept my apologies for the delay in getting back to you as the reports came back when I was on annual leave.
We have received the enclosed reports from the three referees who re-reviewed your manuscript. As you will see, while referees #2 and #3 are satisfied with the revisions, referee #1 still raises a few concerns that should be addressed before acceptance of your manuscript.
Moreover, kindly address the editorial issues listed below.

1/ Referees:
- Please provide a point-by-point letter addressing the remaining concerns from referee #1.

2/ Main manuscript text:
- Please address the queries from our data editors in track changes mode in the main manuscript file labelled 'Data edited MS file'. Kindly use this file for any further modification, remove the blue text and only keep in track changes mode any new modification.
- Material and methods:
o Human samples: include a statement that informed consent was obtained from all subjects and that the experiments conformed to the principles set out in the WMA Declaration of Helsinki and the Department of Health and Human Services Belmont Report.
o Please indicate the origin of the cells.
- Data availability section: Thank you for depositing your data in public repositories. Please update the link for the SRA database.
- Author contribution: CRediT has replaced the traditional author contributions section because it offers a systematic machine-readable author contributions format that allows for more effective research assessment. Please remove the Authors Contributions from the manuscript and use the free text boxes beneath each contributing author's name in our system to add specific details on the author's contribution. More information is available in our guide to authors.
- Please remove the abbreviation list from the manuscript and instead define them the first time they appear in the text.
- The Appendix figure legends should be removed from the main manuscript file.

3/ Checklist:
We have updated the format of our checklist earlier this year. Could you please fill in and provide this document? (https://www.embopress.org/page/journal/17574684/authorguide)

4/ Source Data: Thank you for providing extensive source data. Please zip the source data for each figure individually (1 file/figure)

5/ Thank you for providing a synopsis. I slightly edited your text, please let me know if you agree with the following, or amend as you see fit.

Adaptation to metformin is frequently observed during long-term tumor treatment. Here, during this adaptation process, TOMM34 was upregulated to enhance OXPHOS by interacting with ATP5B. Disruption of the TOMM34-ATP5B interaction using Gboxin synergized with metformin to suppress HCC.
- HCC cells exhibited an EMT phenotype and a compensatory elevation of respiration rate during metformin adaptation.
- TOMM34 upregulation preserved OXPHOS and ATP production during metformin adaptation.
- TOMM34 interacted with ATP5B to maintain OXPHOS and ATP production.
- Gboxin disrupted TOMM34 / ATP5B interaction and prevented metformin adaptation.

6/ As part of the EMBO Publications transparent editorial process initiative (see our Editorial at http://embomolmed.embopress.org/content/2/9/329), EMBO Molecular Medicine will publish online a Review Process File (RPF) to accompany accepted manuscripts.
This file will be published in conjunction with your paper and will include the anonymous referee reports, your point-by-point response and all pertinent correspondence relating to the manuscript. Let us know whether you agree with the publication of the RPF and as here, if you want to remove or not any figures from it prior to publication.
Please note that the Authors checklist will be published at the end of the RPF.

I look forward to reading a new revised version of your manuscript as soon as possible.

Yours sincerely,

Lise Roth

Lise Roth, PhD
Senior Editor
EMBO Molecular Medicine

***** Reviewer's comments *****

Referee #1 (Remarks for Author):

The revised manuscript is improved. However, several points require further

revision.
1) In the abstract, it is now stated that TOMM34 confers energetic flexibility. However, there is no experiment presented in the manuscript that supports this claim.

2) Metformin impacts the redox state of cells by inhibiting complex I, so MTT assay that relies on reduction of a probe is not appropriate. Cell proliferation/quantification should be done with a cell counter.

3) Quantification of western blots, notably for Fig. 4F,G, should be presented with bar graphs and statistics.

4) Figure 5A: the extracellular lactate quantification should be performed in the presence of metformin.

Referee #2 (Comments on Novelty/Model System for Author):

The authrs have adequately addressed all my concerns.

Referee #2 (Remarks for Author):

The authrs have addressed all my concerns. I thus have no more questions.

Referee #3 (Remarks for Author):

I am satisfied with the authors' effort to address all points raised by the three reviewers.

Point-by-point response for EMM-2022-16082-V2

The new modifications of the manuscript are marked in track changes.

***** Reviewer's comments *****

Referee #1 (Remarks for Author):

The revised manuscript is improved. However, several points require further revision.

Re: We would like to thank the reviewer for their critical and valuable comments which have helped us to improve our manuscript substantially.

1) In the abstract, it is now stated that TOMM34 confers energetic flexibility. However, there is no experiment presented in the manuscript that supports this claim.

Re: We have amended the description as "TOMM34, a translocase of the outer mitochondrial membrane, was upregulated to promote tumor metastasis in response to metformin-induced metabolic stress". (**Line 54-55**).

2) Metformin impacts the redox state of cells by inhibiting complex I, so MTT assay that relies on reduction of a probe is not appropriate. Cell proliferation/quantification should be done with a cell counter.

Re: Cell proliferation/quantification has been evaluated by a cell counter according to your kind suggestion (**Fig. 1E, Fig. 2E**). We have rephrased the relevant descriptions in Data edited MS file (**line 1002-1004, 1034**).

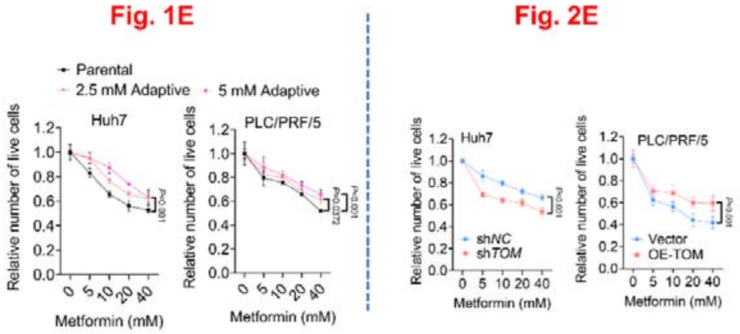

Fig. 1. (E) The relative number of living cells was evaluated after the parental or metformin adaptive HCC cells were treated with indicated concentration of metformin for 24 hours. (n=4, Two-way ANOVA).

Fig. 2. (E) Relative number of living cells showing the effects of TOMM34 on the proliferation of HCC cells treated with the indicated concentration of metformin for 24 hours (sh*TOM*: sh*TOMM34*, TOMM34 knockdown cells; OE-TOM: OE-TOMM34, TOMM34 overexpressed cells). (n=4, Two-way ANOVA).

3) Quantification of western blots, notably for Fig. 4F,G, should be presented with bar graphs and statistics.

Re: We have modified this accordingly in **Fig. 4F** and **4G**.

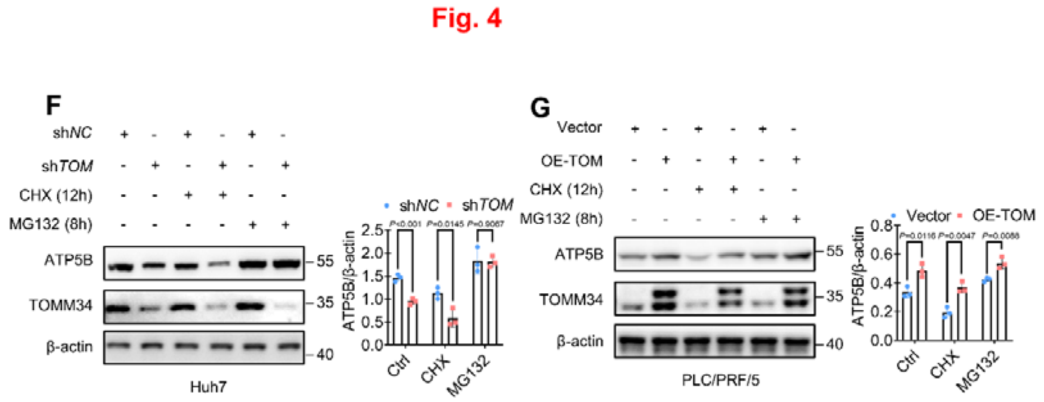

Fig. 4. (F-G) Western blot showing protein levels of ATP5B in Huh7 (F) or PLC/PRF/5 (G) cells treated with or without 10 mg/mL cyclohexane (CHX) or 25 μM MG132 for indicated period.

4) Figure 5A: the extracellular lactate quantification should be performed in the presence of metformin.

We have quantified extracellular lactate in the presence of metformin as showed in **Response Fig. 1A** in this version. In the absence of metformin, the level of extracellular lactate was not obviously changed between parental and adaptive cells. However, in the presence of metformin, the level of extracellular lactate was elevated in both parental and adaptive cells.

Response Fig. 1

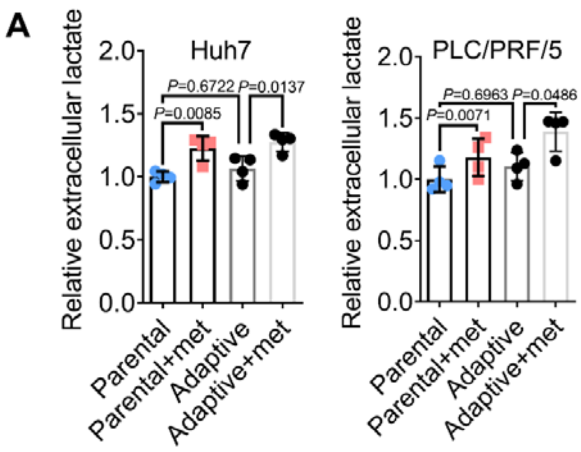

Response Fig. 1. (A) Lactate levels in indicated cell supernatants was measured using a lactate detection kit.

Referee #2 (Comments on Novelty/Model System for Author):

The authors have adequately addressed all my concerns.

Referee #2 (Remarks for Author):

The authors have addressed all my concerns. I thus have no more questions.

Re: We thank the reviewer for their valuable comments for helping us to strengthen our manuscript.

Referee #3 (Remarks for Author):

I am satisfied with the authors' effort to address all points raised by the three reviewers.

Re: We thank Reviewer #3 for the encouraging and constructive comments.

12th Oct 2022

Dear Dr. Huang,

Thank you for sending the data-edited file. I am pleased to inform you that your manuscript is now accepted for publication and will be sent to our publisher to be included in the next available issue of EMBO Molecular Medicine.

Congratulations on your interesting work,

With kind regards,

Lise Roth

Lise Roth, Ph.D
Senior Editor
EMBO Molecular Medicine

***** Reviewer's comments *****

Referee #1:

Remarks for Author:

Is suitable for publication

EMBO Press Author Checklist

Corresponding Author Name: Canhua Huang	
Journal Submitted to: EMBO Molecular Medicine	
Manuscript Number: EMM-2022-16082	

USEFUL LINKS FOR COMPLETING THIS FORM
The EMBO Journal - Author Guidelines
EMBO Reports - Author Guidelines
Molecular Systems Biology - Author Guidelines
EMBO Molecular Medicine - Author Guidelines

Reporting Checklist for Life Science Articles (updated January 2022)
This checklist is adapted from Materials Design Analysis Reporting (MDAR) Checklist for Authors. MDAR establishes a minimum set of requirements in transparent reporting in the life sciences (see Statement of Task: 10.31222/osf.io/9sm4x). Please follow the journal's guidelines in preparing your manuscript.
Please note that a copy of this checklist will be published alongside your article.

Abridged guidelines for figures
1. Data
The data shown in figures should satisfy the following conditions:
- the data were obtained and processed according to the field's best practice and are presented to reflect the results of the experiments in an accurate and unbiased manner.
- ideally, figure panels should include only measurements that are directly comparable to each other and obtained with the same assay.
- plots include clearly labeled error bars for independent experiments and sample sizes. Unless justified, error bars should not be shown for technical replicates.
- if n<5, the individual data points from each experiment should be plotted. Any statistical test employed should be justified.
- Source Data should be included to report the data underlying figures according to the guidelines set out in the authorship guidelines on Data Presentation.

2. Captions
Each figure caption should contain the following information, for each panel where they are relevant:
- a specification of the experimental system investigated (eg cell line, species name).
- the assay(s) and method(s) used to carry out the reported observations and measurements.
- an explicit mention of the biological and chemical entity(ies) that are being measured.
- an explicit mention of the biological and chemical entity(ies) that are altered/varied/perturbed in a controlled manner.
- the exact sample size (n) for each experimental group/condition, given as a number, not a range;
- a description of the sample collection allowing the reader to understand whether the samples represent technical or biological replicates (including how many animals, litters, cultures, etc.).
- a statement of how many times the experiment shown was independently replicated in the laboratory.
- definitions of statistical methods and measures:
  - common tests, such as t-test (please specify whether paired vs. unpaired), simple χ2 tests, Wilcoxon and Mann-Whitney tests, can be unambiguously identified by name only, but more complex techniques should be described in the methods section;
  - are tests one-sided or two-sided?
  - are there adjustments for multiple comparisons?
  - exact statistical test results, e.g., P values = x but not P values < x;
  - definition of 'center values' as median or average;
  - definition of error bars as s.d. or s.e.m.

Please complete ALL of the questions below.
Select "Not Applicable" only when the requested information is not relevant for your study.

Materials

Newly Created Materials	Information included in the manuscript?	In which section is the information available? (Reagents and Tools Table, Materials and Methods, Figures, Data Availability Section)
New materials and reagents need to be available; do any restrictions apply?	Not Applicable	

Antibodies	Information included in the manuscript?	In which section is the information available? (Reagents and Tools Table, Materials and Methods, Figures, Data Availability Section)
For **antibodies** provide the following information:<br>- Commercial antibodies: RRID (if possible) or supplier name, catalogue number and or/clone number<br>- Non-commercial: RRID or citation	Yes	Materials and Methods

DNA and RNA sequences	Information included in the manuscript?	In which section is the information available? (Reagents and Tools Table, Materials and Methods, Figures, Data Availability Section)
**Short novel DNA or RNA including primers, probes**: provide the sequences.	Not Applicable	

Cell materials	Information included in the manuscript?	In which section is the information available? (Reagents and Tools Table, Materials and Methods, Figures, Data Availability Section)
**Cell lines:** Provide species information, strain. Provide accession number in repository **OR** supplier name, catalog number, clone number, and/**OR** RRID.	Yes	Materials and Methods
**Primary cultures:** Provide species, strain, sex of origin, genetic modification status.	Not Applicable	
Report if the cell lines were recently **authenticated** (e.g., by STR profiling) and tested for mycoplasma contamination.	Not Applicable	

Experimental animals	Information included in the manuscript?	In which section is the information available? (Reagents and Tools Table, Materials and Methods, Figures, Data Availability Section)
**Laboratory animals or Model organisms:** Provide species, strain, sex, age, genetic modification status. Provide accession number in repository **OR** supplier name, catalog number, clone number, **OR** RRID.	Yes	Materials and Methods
**Animal observed in or captured from the field:** Provide species, sex, and age where possible.	Not Applicable	
Please detail **housing and husbandry conditions**.	Yes	Materials and Methods

Plants and microbes	Information included in the manuscript?	In which section is the information available? (Reagents and Tools Table, Materials and Methods, Figures, Data Availability Section)
**Plants:** provide species and strain, ecotype and cultivar where relevant, unique accession number if available, and source (including location for collected wild specimens).	Not Applicable	
**Microbes:** provide species and strain, unique accession number if available, and source.	Not Applicable	

Human research participants	Information included in the manuscript?	In which section is the information available? (Reagents and Tools Table, Materials and Methods, Figures, Data Availability Section)
If collected and within the bounds of privacy constraints report on age, sex and gender or ethnicity for all study participants.	Not Applicable	

Core facilities	Information included in the manuscript?	In which section is the information available? (Reagents and Tools Table, Materials and Methods, Figures, Data Availability Section)
If your work benefited from core facilities, was their service mentioned in the acknowledgments section?	Not Applicable	

Design

Study protocol	Information included in the manuscript?	In which section is the information available? (Reagents and Tools Table, Materials and Methods, Figures, Data Availability Section)
If study protocol has been **pre-registered, provide DOI in the manuscript**. For clinical trials, provide the trial registration number **OR** cite DOI.	Not Applicable	
Report the **clinical trial registration number** (at ClinicalTrials.gov or equivalent), where applicable.	Not Applicable	

Laboratory protocol	Information included in the manuscript?	In which section is the information available? (Reagents and Tools Table, Materials and Methods, Figures, Data Availability Section)
Provide DOI OR other citation details if **external detailed step-by-step protocols** are available.	Not Applicable	

Experimental study design and statistics	Information included in the manuscript?	In which section is the information available? (Reagents and Tools Table, Materials and Methods, Figures, Data Availability Section)
Include a statement about **sample size** estimate even if no statistical methods were used.	Yes	Materials and Methods/Figures
Were any steps taken to minimize the effects of subjective bias when allocating animals/samples to treatment (e.g. **randomization procedure**)? If yes, have they been described?	Yes	Materials and Methods
Include a statement about **blinding** even if no blinding was done.	Not Applicable	
Describe **inclusion/exclusion criteria** if samples or animals were excluded from the analysis. Were the criteria pre-established?<br><br>If sample or data points were omitted from analysis, report if this was due to attrition or intentional exclusion and provide justification.	Not Applicable	
For every figure, are **statistical tests** justified as appropriate? Do the data meet the assumptions of the tests (e.g., normal distribution)? Describe any methods used to assess it. Is there an estimate of variation within each group of data? Is the variance similar between the groups that are being statistically compared?	Yes	Materials and Methods/Figures

Sample definition and in-laboratory replication	Information included in the manuscript?	In which section is the information available? (Reagents and Tools Table, Materials and Methods, Figures, Data Availability Section)
In the figure legends: state number of times the experiment was **replicated** in laboratory.	Yes	Figures
In the figure legends: define whether data describe **technical or biological replicates**.	Yes	Figures

Ethics

Ethics	Information included in the manuscript?	In which section is the information available? (Reagents and Tools Table, Materials and Methods, Figures, Data Availability Section)
Studies involving **human participants**: State details of **authority granting ethics approval** (IRB or equivalent committee(s), provide reference number for approval.	Not Applicable	
Studies involving **human participants**: Include a statement confirming that **informed consent** was obtained from all subjects and that the experiments conformed to the principles set out in the WMA Declaration of Helsinki and the Department of Health and Human Services Belmont Report.	Not Applicable	
Studies involving **human participants:** For publication of **patient photos**, include a statement confirming that consent to publish was obtained.	Not Applicable	
Studies involving experimental **animals**: State details of **authority granting ethics approval** (IRB or equivalent committee(s), provide reference number for approval. Include a statement of compliance with ethical regulations.	Yes	Materials and Methods
Studies involving **specimen and field samples:** State if relevant **permits** obtained, provide details of authority approving study; if none were required, explain why.	Yes	Materials and Methods

Dual Use Research of Concern (DURC)	Information included in the manuscript?	In which section is the information available? (Reagents and Tools Table, Materials and Methods, Figures, Data Availability Section)
Could your study fall under dual use research restrictions? Please check biosecurity documents and list of **select agents and toxins** (CDC): https://www.selectagents.gov/sat/list.htm	Not Applicable	
If you used a select agent, is the security level of the lab appropriate and reported in the manuscript?	Not Applicable	
If a study is subject to dual use research of concern regulations, is the name of the **authority granting approval and reference number** for the regulatory approval provided in the manuscript?	Not Applicable	

Reporting

The MDAR framework recommends adoption of discipline-specific guidelines, established and endorsed through community initiatives. Journals have their own policy about requiring specific guidelines and recommendations to complement MDAR.

Adherence to community standards	Information included in the manuscript?	In which section is the information available? (Reagents and Tools Table, Materials and Methods, Figures, Data Availability Section)
State if relevant guidelines or checklists (e.g., **ICMJE, MIBBI, ARRIVE, PRISMA**) have been followed or provided.	Not Applicable	
For **tumor marker prognostic studies,** we recommend that you follow the **REMARK** reporting guidelines (see link list at top right). See author guidelines, under 'Reporting Guidelines'. Please confirm you have followed these guidelines.	Not Applicable	
For **phase II and III randomized controlled trials**, please refer to the **CONSORT** flow diagram (see link list at top right) and submit the CONSORT checklist (see link list at top right) with your submission. See author guidelines, under 'Reporting Guidelines'. Please confirm you have submitted this list.	Not Applicable	

Data Availability

Data availability	Information included in the manuscript?	In which section is the information available? (Reagents and Tools Table, Materials and Methods, Figures, Data Availability Section)
Have **primary datasets** been deposited according to the journal's guidelines (see 'Data Deposition' section) and the respective accession numbers provided in the Data Availability Section?	Yes	Data Availability Section
Were **human clinical and genomic datasets** deposited in a public access-controlled repository in accordance to ethical obligations to the patients and to the applicable consent agreement?	Yes	Figures/Data Availability Section
Are **computational models** that are central and integral to a study available without restrictions in a machine-readable form? Were the relevant accession numbers or links provided?	Not Applicable	
If publicly available data were reused, provide the respective **data citations in the reference list**.	Not Applicable	
